# Metabolic and Glycemic Effects of Orforglipron, a GLP‐1 Receptor Agonist, in Adults With or Without Diabetes: A Network Meta‐Analysis of Randomised Clinical Trials

**DOI:** 10.1002/edm2.70275

**Published:** 2026-07-22

**Authors:** Ahmed W. Hageen, Ahmed Farid Gadelmawla, Ahmad Omar Saleh, Mohamed Reyad Mohamed, Abdallfatah Abdallfatah, Ahmed Elsekhary, Amira Fahmy El‐Nemr, Safir Eladawi, Odai Maihoub, Hind Abdulhay, Mustafa Turkmani, Basel Abdelazeem, Gregg C. Fonarow

**Affiliations:** ^1^ Faculty of Medicine Tanta University Tanta Egypt; ^2^ Faculty of Medicine Menoufia University Menoufia Egypt; ^3^ Medical Research Group of Egypt (MRGE), Negida Academy Arlington Massachusetts USA; ^4^ Faculty of Medicine The University of Jordan Amman Jordan; ^5^ Department of Medicine University of Arizona College of Medicine—Phoenix Phoenix Arizona USA; ^6^ Banner Desert Medical Center Mesa Arizona USA; ^7^ Faculty of Medicine October 6 University Giza Egypt; ^8^ Kasr Alainy School of Medicine Cairo University Cairo Egypt; ^9^ Faculty of Medicine Al‐Azhar University Cairo Egypt; ^10^ Faculty of Medicine Ain Shams University Cairo Egypt; ^11^ Department of Pathology National Hospital Latakia Syria; ^12^ Faculty of Medicine Mansoura University Daqahliyah Egypt; ^13^ Division of Pulmonary and Critical Care University of Toledo Toledo Ohio USA; ^14^ Faculty of Medicine Michigan State University East Lansing Michigan USA; ^15^ Department of Cardiology West Virginia University Morgantown West Virginia USA; ^16^ University of California Los Angeles Los Angeles California USA; ^17^ Ahmanson‐UCLA Cardiomyopathy Center, Ronald Reagan UCLA Medical Center Los Angeles California USA

**Keywords:** body weight, glucagon‐like peptide‐1 receptor agonists, HbA1c, obesity, orforglipron

## Abstract

**Background and Aim:**

Orforglipron (OFG), an oral, non‐peptide glucagon‐like peptide‐1 receptor agonist (GLP‐1 RAs), showed potential benefits for type 2 diabetes mellitus (T2DM) and obesity. Its dose–response effects on body weight‐related parameters and glycemic outcomes remain incompletely analysed. This network meta‐analysis aims to address this gap across multiple doses of OFG (3, 12, 24, 36 and 45 mg) in adults with or without T2DM at 12, 26 and 36 weeks.

**Methods:**

PRISMA guidelines were followed in our study. Embase, PubMed, Web of Science and Scopus were searched for randomised controlled trials. Random‐effects models expressed OFG effects as odds ratios (OR), mean difference (MD) and standardised mean difference (SMD) with 95% confidence intervals (95% CI). RStudio software (version 4.5.1) was used for analysis.

**Results:**

Six RCTs comprising 4878 participants were included. OFG demonstrated reductions in body weight, BMI and waist circumference across all follow‐ups. OFG 45 mg dose produced the greatest effects in body weight (SMD: −1.71 kg at 12 weeks, MD: −8.81 kg at 26 weeks and MD: −12.13 kg at 36 weeks vs. placebo). Categorical weight‐loss analyses showed that individuals receiving 24–45 mg increased the odds to achieve ≥ 5%, ≥ 10% and ≥ 15% weight loss at 26 weeks. Glycemic outcomes improved across all doses, with the greatest HbA1c reduction at 45 mg (−1.65%; 95% CI −1.98 to −1.32) and greatest fasting glucose improvement at 36 mg dose. Treatment‐emergent adverse events increased with dose.

**Conclusion:**

OFG demonstrated improvements in weight‐related outcomes and glycemic outcomes. Adverse events increased with dose, consistent with expected class tolerability.

## Graphical abstract

In a network meta‐analysis of six randomised controlled trials, oral orforglipron demonstrated potential reductions in body weight, BMI, waist circumference and glycemic control versus placebo. Higher doses achieved the greatest efficacy, with an overall safety profile consistent with the GLP‐1 receptor agonist class.

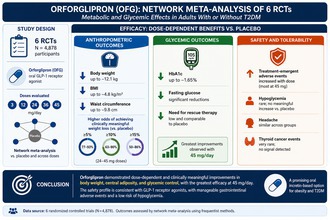

## 1 Introduction

Obesity is a chronic, relapsing disease that increases the risk of type 2 diabetes mellitus (T2DM) and other cardiometabolic complications and contributes to substantial long‐term morbidity [1]. Sustained weight reduction improves glycemic control and metabolic risk factors, but lifestyle intervention alone often does not achieve durable benefit for many patients [1, 2]. Recent reviews and guidelines emphasised that obesity is a chronic, relapsing disease affecting over 500 million adults and requiring early, individualised, and long‐term management beyond BMI‐based assessment [3, 4]. These frameworks highlight the expanding role of pharmacotherapy, particularly GLP‐1–based agents in improving metabolic and glycemic outcomes, supporting the clinical relevance of evaluating novel oral GLP‐1 receptor agonists such as, orforglipron in patients with and without diabetes.

Orforglipron (OFG) is a once daily oral small molecule, nonpeptide GLP‐1 RA developed to simplify administration and potentially broaden acceptability compared with injectable therapies [5, 6]. Randomised trials in adults with obesity without diabetes have shown clinically relevant weight loss and improvements in metabolic measures over medium‐term follow‐up [5, 7]. In adults with T2DM, phase 2 data have similarly demonstrated improvements in glycemic and weight outcomes with a gastrointestinal adverse event profile consistent with the GLP‐1 class [8]. More recently, longer duration randomised evidence in adults with obesity without diabetes has extended the trial base for weight loss and cardiometabolic endpoints and strengthens the rationale for comparative evaluation across available therapies [5].

Despite an expanding incretin‐based treatment landscape, comparative metabolic profiles remain uncertain because head‐to‐head trials are limited, trial populations differ by diabetes status, and background therapies can modify glycemic outcomes. Clinicians often need to weigh weight reduction against broader metabolic effects such as glycated haemoglobin, fasting glucose, lipid fractions and blood pressure when selecting therapy for obese adults with and without T2DM [9, 10]. Current major guidelines recognise GLP‐1‐based therapies as effective options for obesity and diabetes‐related weight management, but they do not resolve comparative questions for newer oral agents across multiple metabolic outcomes [11, 12, 13].

A dose‐level network meta‐analysis (NMA) is crucial because different doses of the same drug can have different effects, which cannot be observed in drug‐level comparisons. Since no direct head‐to‐head trials comparing doses are available, this approach allows indirect comparison of dose‐specific efficacy and safety using all existing evidence. Accordingly, we conducted a NMA to evaluate the metabolic effects of OFG in adults with or without T2DM and to compare outcomes across OFG dose regimens for body weight and key metabolic and glycemic outcomes through advanced statistical approaches.

## 2 Methodology

### 2.1 Protocol Registration

This network meta‐analysis (NMA) was conducted following the Cochrane Handbook for Systematic Reviews of Interventions [14] and adhered to the PRISMA guidelines [15]. The protocol for this study was prospectively registered at PROSPERO with (ID: CRD420251273456). The ethical approval and institutional review board were waived because the investigators relied on the data from published articles.

### 2.2 Data Sources and Search Strategy

**FIGURE 1:**
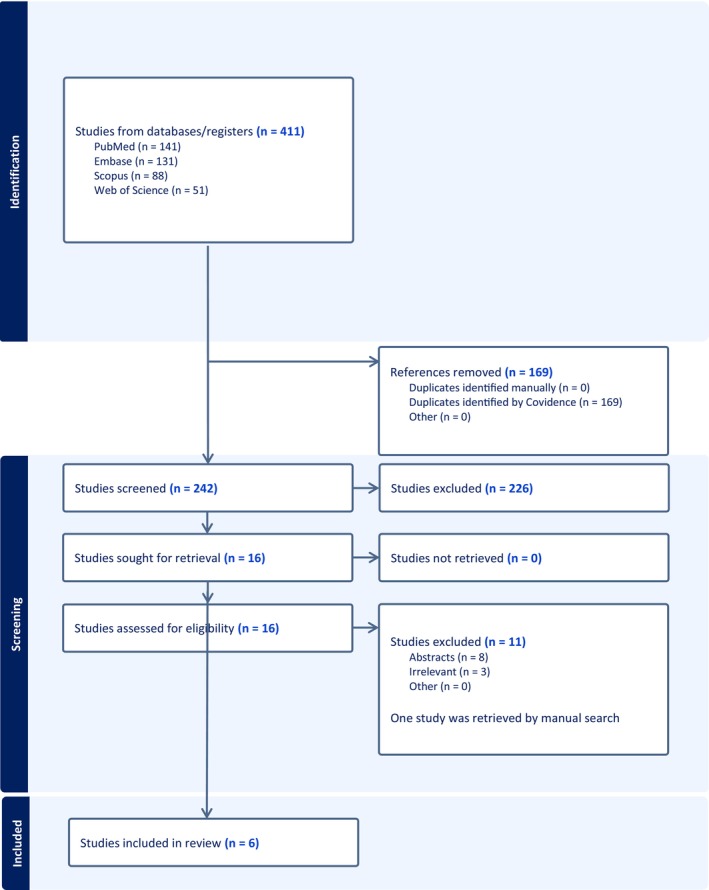
PRISMA flow chart of the screening process.

We systematically searched four electronic databases: PubMed, Embase, Scopus and Web of Science through 21 October 2025 with the following search terms (‘orforglipron’ OR ‘LY3502970’) AND (‘type 2 diabetes’ OR ‘T2DM’ OR ‘diabetes mellitus’ OR ‘diabetes’ OR ‘obesity’ OR ‘obese’ OR ‘overweight’), as shown in Table S1 to retrieve the available RCTs that assess the metabolic and glycemic effects of OFG in adults with or without type 2 diabetes mellitus (T2DM). We did an updated search on 22 November 2025 and found no more trials published. No restrictions were placed on the language, publication date or geographic location. Further manual search was adopted using the search strategy on Google Scholar. Also, it was applied to the references and citations of included studies for any studies that were missed from the primary search. However, the PRISMA flow chart and checklist are summarised in Figure 1 and Table S2.

### 2.3 Eligibility Criteria

Studies were included if they met the following PICOS framework:

- *Population* (P): adults (> 18 years) with or without T2DM.
- *Intervention* (I): oral OFG administration (3, 12, 24, 36 and 45 mg) at 12, 26 and 36 weeks.
- *Comparator* (C): placebo and indirectly, other OFG doses in NMA.
- *Outcomes* (O):
  – Primary outcomes (weight‐related): body mass index (BMI) in kg/m^2^, waist circumference (WC) in cm, total body weight in kg, categorical weight loss thresholds (≥ 5%, ≥ 10%, ≥ 15%).
  – Secondary outcomes (glycemic): haemoglobin A1c (HbA1c) (%), fasting blood glucose (FBG) in mg/dl, need for rescue therapy for persistent hyperglycemia.
  – Safety outcomes: treatment‐emergent adverse events (TEAEs), hypoglycemia, headache, thyroid cancer.
- *Study designs* (S): RCTs only.

The exclusion criteria included animal studies, observational designs (cohort or case–control), incomplete data, conference abstracts, narrative reviews, books, theses, non‐randomised or quasi‐experimental studies, protocols, letters, case reports, case series and duplicate publications or studies with overlapping patient cohorts.

### 2.4 Study Selection

After retrieving the records from databases, two independent authors (A.A. and A.W.H.) screened the records using the Covidence software [16] in two phases: title and abstract, followed by full‐text screening. We included RCTs that evaluate the metabolic and glycemic effects of OFG in individuals with or without T2DM.

### 2.5 Data Extraction

Four independent authors (A.F.E., O.M., A.E. and M.R.M) extracted the data using a standardised Excel spreadsheet. The extracted data included the following:

- Study characteristics: (study ID, trial name, design, phase, blinding, register number, location, number of centres, total patients, study arms, OFG route of administration, eligibility criteria, primary and secondary outcomes, and follow‐up duration).
- Baseline participant variables: number of participants, age in years, sex, weight, race/ethnicity, BMI, WC, prior glucose‐lowering therapy and serum calcitonin.
- Primary and secondary outcomes as mentioned previously.

Any conflicts were resolved through consensus with a third reviewer (A.W.H.) through a discussion.

### 2.6 Assessment of the Quality of Included Studies

**FIGURE 2:**
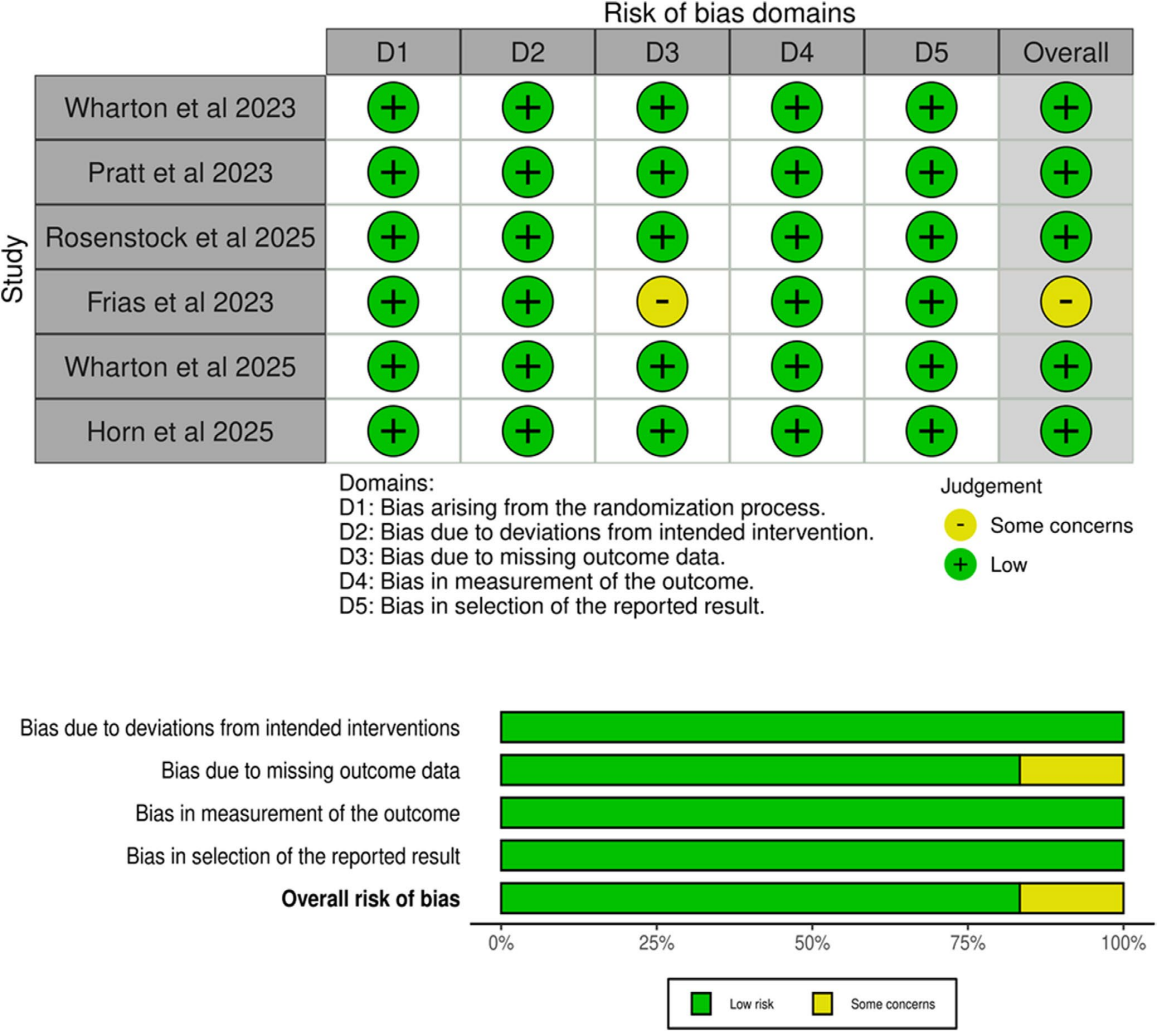
Quality assessment of risk of bias in the included trials. The upper panel presents a schematic representation of risks (low = green and some concerns = yellow) for specific types of biases of each study in the review. The lower panel presents risks (low = green and some concerns = yellow) for the subtypes of biases of the combination of studies included in this review.

Two authors independently (M.R.M. and A.W.H.) assessed the methodological quality of the included RCTs using the Cochrane Risk of Bias 2 (ROB2) tool [17]. This evaluation covered five key domains: randomisation, adherence to the assigned intervention, handling of missing outcome data, measurement of outcomes and selective reporting. Each domain was rated as ‘low risk’ or ‘some concerns’ leading to an overall judgement (Figure 2). Any conflicts were resolved by consulting a third author (A.F.G.). Following the Cochrane recommendations, we did not perform a formal assessment of publication bias because fewer than 10 studies met the inclusion criteria [14].

### 2.7 The Geometry of the Network

We presented a network graph with six nodes representing the doses of OFG. The edges represent available direct comparisons between pairs of routes. The line thickness indicates the number of studies involved in these comparisons. The plotted number represents the number of comparisons between each node.

### 2.8 Data Synthesis and Analysis

The frequentist method [18] was employed to conduct NMA of the included studies, comparing different doses of OFG. Random effects models were used in our research to estimate treatment effects as odds ratios (ORs) and Mean Differences (MDs) or Standardised Mean Differences (SMDs), with corresponding 95% confidence intervals (95% CIs). We calculated the surface under the cumulative ranking curve (SUCRA) for each outcome. The relative ranking of the different doses to determine efficacy and safety was estimated based on the distribution of the SUCRAs. Higher SUCRA values reflected superior performance, as evidenced by a lower incidence of treatment‐emergent adverse events and greater reductions in body weight, BMI, WC and glycemic parameters. We utilised the *I* ^2^ statistic to assess heterogeneity in the network analysis. Subgroup analyses were conducted to explore potential differences in treatment effects according to diabetes status (patients with and without DM). Comparisons were made among various OFG dose groups (3, 12, 24, 36 and 45 mg) and placebo, using random‐effects models. Placebo was used as the reference comparator in the NMA. Inconsistency in the model was evaluated by comparing estimates from direct and indirect comparisons. We compared the distributions of key study characteristics across studies grouped by contrast to evaluate transitivity. Statistical inconsistencies between direct and indirect evidence were examined using a combination of global and local approaches. As a global approach, we applied a design‐by‐treatment interaction model to investigate inconsistencies from all sources in the entire network [19, 20]. Local inconsistency was evaluated based on the node‐splitting method [21]. Statistical analyses were conducted using the ‘netmeta’ package (version 4.5.1) within RStudio software [22, 23]. A *p* value of less than 0.05 was considered statistically significant. Sensitivity analysis was conducted by excluding studies with small sample sizes and by excluding phase I [24] and II studies [7, 8]. For studies that did not report numerical outcome data, values were extracted from published figures using a validated graphical extraction tool (WebPlotDigitizer) [25]. Two independent reviewers performed the digitisation process by calibrating axes and manually extracting data points for weight‐related and glycemic outcomes at each reported follow‐up. When discrepancies occurred, consensus was reached through discussion. Extracted values were then converted into mean and standard deviation, when necessary, using established statistical method (meta accelerator) [26]. Specifically, reported summary statistics were transformed into approximate means and standard deviations to allow for consistent inclusion in the meta‐analysis. These steps ensured comparability of outcome measures across studies and facilitated convergence in the pooled analyses. The CFB in metabolic and glycemic outcomes, including HbA1C (%), WC (cm), FBG (mg/dL), BMI (kg/m^2^) and body weight outcome in Pratt et al. 2023 (kg; Week 12) were reported as absolute CFB. However, the CFB in body weight (kg) in all included studies except Pratt et al. 2023 were reported in %CFB.

## 3 Results

### 3.1 Search Results and Study Selection

An electronic search across four databases yielded 411 references. After automated duplicate removal in Covidence, 169 references were excluded; no duplicate reference was removed manually. From a total of 242 references, 226 records were excluded after title and abstract screening. Ultimately, 16 references were assessed via full‐text review; however, 11 studies were excluded after full‐text screening, and five studies met the eligibility criteria. Additionally, one study was retrieved by manual search. Therefore, six studies included in the final analysis (Figure 1).

### 3.2 Characteristics of Included Studies

Six RCTs comprising 4878 participants were included in the data synthesis [5, 7, 8, 24, 27, 28]. A summary of characteristics of included trials is presented in (Table 1), and baseline characteristics of participants are presented in (Table 2). The OFG group consisted of 3039 patients, while the control group consisted of 1839 patients. The mean age of patients in the OFG group was 55.25 years, ranging from 44.9 to 62.8 years. However, the mean age for each OFG dose (3, 12, 24, 36 and 45 mg) was 56.2, 52.3, 58.8, 54.3 and 58.4 years, respectively. Besides, the placebo group had a mean age of 53.9 years, ranging from 45.1 to 58.3 years.

**TABLE 1:** Summary and characteristics of included studies.

Study ID	Design, centres	Trial registry ID	Region (s)	Control details	Intervention details	Inclusion criteria	Outcomes	Sample size^a^ (OFG/Placebo)	Follow‐up, weeks	Main conclusion
Wharton et al. 2025 (ATTAIN‐1) [5]	RCT, multicentre	NCT05869903	Brazil, China, India, Japan, Korea, Slovakia, Spain, Taiwan, and USA	All participants received a matching placebo once daily to allow a clear assessment of OFG's efficacy and safety against a non‐active treatment for weight loss and health outcomes	Oral OFG once daily at doses of 6, 12 or 36 mg, alongside an intervention emphasising weight control and lifestyle optimisation	Adults aged ≥ 18 years were eligible if they were obese (BMI ≥ 30 kg/m^2^) or overweight (BMI: 27–30 kg/m^2^) with an obesity‐related condition (e.g., hypertension), and had a history of unsuccessful dietary weight‐loss attempts.	The change in body weight from baseline to week 72	(1455/949)	72 weeks of OFG treatment and had an additional 2‐week safety follow‐up (74 weeks)	OFG led to significantly greater weight loss than placebo in adults with obesity, exhibiting a typical GLP‐1RA adverse‐event profile
Rosenstock et al. 2025 (ACHIEVE‐1) [27]	RCT, multicentre	NCT05971940	China, India, Japan, Mexico, and USA	All participants received placebo once daily following the same dose escalation schedule as the active group, and like the OFG group, they had a 2‐week safety follow‐up period with no de‐escalation permitted, though temporary OFG pauses were advised for GI AEs	Oral OFG at 1 mg, with stepwise titration every 4 weeks through 3, 6, 12, 24 and 36 mg until the assigned maintenance dose (3, 12 or 36 mg once daily) was achieved. Temporary dose interruptions were allowed to manage gastrointestinal adverse events without dose reduction, followed by a two‐week post‐treatment safety follow‐up	T2DM participants age ≥ 18 years were required to be on diet/exercise only (no insulin or other glucose‐lowering drugs in 3 months), with HbA1c 7.0%–9.5%, BMI ≥ 23 kg/m^2^ and stable weight (≤ 5% change) for 3 months	The change in the HbA1c level from baseline to week 40	(421/138)	40 weeks of OFG treatment followed by a 2‐week safety follow‐up (42 weeks)	OFG significantly lowered HbA1c over 40 weeks in adults with T2DM
Frias et al. 2023 [8]	RCT, multicentre	NCT05048719	USA, Hungary, Poland, and Slovakia	All participants received placebo once daily and followed the same dose escalation schedule as the OFG groups	Oral OFG (3, 12, 24, 36 or 45 mg maintenance doses), or placebo. The OFG 36 and 45 mg groups were further stratified into subgroups to evaluate varying starting doses and dose‐escalation schedules, with all participants receiving standardised lifestyle guidance and safety education	Participants aged ≥ 18 years with T2DM were included if their HbA1c was 7.0%–10.5%. They were on diet/exercise ± stable metformin (≥ 3 months), and had a BMI ≥ 23 kg/m^2^ and stable weight (≤ 5% change) for 3 months.	The change in HbA1c from baseline to week 26	(278/55)	40‐week treatment period and a 2‐week post‐treatment follow‐up (42 weeks)	OFG significantly reduced HbA1c and weight, with adverse events comparable to other GLP‐1RAs, making it a convenient oral option for T2DM
Wharton et al. 2023 (GZGI) [7]	RCT, multicentre	NCT05051579	Canada, USA, and Hungary.	All participants received placebo once daily for 36 weeks, following the same dose escalation schedule as the OFG groups, with no meal restrictions, and all participants were provided lifestyle education	Oral OFG at doses of 12, 24, 36 or 45 mg, or placebo, with gradual dose titration over up to 16 weeks, beginning at 2 or 3 mg in the 36‐ and 45‐mg groups. Treatment administration was independent of meals, and all participants were provided with standardised lifestyle counselling.	Participants aged ≥ 18 years had no T2DM (HbA1c < 6.5) but were either obese (BMI ≥ 30 kg/m^2^) or overweight (BMI: 27 to < 30 kg/m^2^) with at least one coexisting condition (e.g., hypertension, dyslipidemia), and had maintained stable weight (≤ 5% change) for 3 months	The change from baseline in body weight at week 26	(222/50)	36 weeks of OFG treatment and a 2‐week post‐treatment follow‐up (38 weeks)	OFG achieved weight loss with an adverse‐event profile comparable to injectable GLP‐1RAs
Pratt et al. 2023 [24]	RCT, multicentre	NCT04426474	USA and Germany	All participants received placebo once daily and followed the same dose escalation schedule as the OFG group	Oral OFG once daily, with the first cohort undergoing weekly dose increases from 3 to 21 mg over 4 weeks. Later cohorts likewise initiated treatment at 3 mg with weekly titration, achieving maintenance doses of 9, 15, 27 or 45 mg within 4–6 weeks	T2D participants aged ≥ 18 years were eligible if treated with diet/exercise ± stable metformin, with HbA1c 7.0%–10.5%, BMI: 18.5–45 kg/m^2^, and stable weight for 3 months	The safety and tolerability of multiple oral doses of OFG in participants with T2D	(9/17)	12‐week OFG treatment period and a 1‐ to 2‐week follow‐up period (14 weeks)	OFG reduced HbA1c and weight with typical GLP‐1RA adverse events, offering a safe, once‐daily oral alternative to injectable or restricted oral GLP‐1RAs
Horn et al. 2025 (ATTAIN‐2) [28]	RCT, multicentre	NCT05872620	Argentina, Australia, Brazil, China, Czech Republic, Germany, Greece, India, South Korea, and the USA.	Matching placebo administered once daily alongside identical individualised counselling (dietary and physical activity) with participants randomised in a 1:1:1:2 ratio (6 mg, 12 mg, 36 mg or placebo) to provide a larger safety evaluation group	Oral OFG once daily at doses of 6, 12 or 36 mg; includes a 20‐week escalation starting at 1 mg (increasing every 4 weeks) alongside lifestyle counselling (portion control, protein/fibre intake, and ≥ 150 min/week physical activity) taken without food or water restrictions.	Adults (≥ 18 years) with T2DM, a BMI of ≥ 27 kg/m^2^, HbA1c 7%–10% (53–86 mmol/mol), stable body weight (< 5% change) for at least 3 months, and prior treatment with only diet/exercise or a stable dose of metformin (with or without an SGLT2 inhibitor).	Primary focus on bodyweight change at week 72; secondary measures included weight loss thresholds. HbA1c/glucose reduction and cardiometabolic/safety profiles. Events.	(654/630)	72 weeks of treatment, followed by a 4‐week safety follow‐up period (totalling 76 weeks).	OFG significantly reduced body weight (up to 12.0%) and HbA1c (up to 2.1%) with a safety profile typical of GLP‐1 RAs.

Abbreviations: BMI, body mass index; GI, gastrointestinal; GLP‐1RAs, glucagon‐like peptide‐1 receptor agonists; HbA1c, haemoglobin; OFG, orforglipron; RCT, randomised controlled trial; T2DM, type 2 diabetes mellitus.

^a^ Included doses of OFG: 3, 12, 24, 36 and 45 mg, along with placebo only.

**TABLE 2:** Baseline characteristics of the included studies.^a^

Study ID	Groups	Number of participants	Age —year	Male, *n* (%)	Weight—kg	BMI, kg/m^2^	WC, cm	Prior glucose‐lowering therapy, *n* (%)	Fasting glucose level—mg/dL	HbA1c, %	Serum calcitonin—ng/L	Race/ethnicity, *n* (%)					Duration of T2DM—year
												H/L	White	AI/AN	Asian	B/AA	
Wharton et al. 2025 (ATTAIN‐1) [5]	OFG 12 mg	725	45.4 ± 12.6	258 (35.6)	102.2 ± 21.6	36.7 ± 6.5	112.0 ± 14.2	NA	92.1 ± 10.0	5.6 ± 0.3	1.1 ± 1.08^c^	275 (37.9)	405 (56.6)	NA	201 (28.1)	60 (8.4)	NA
	OFG 36 mg	730	44.9 ± 11.9	265 (36.3)	103.1 ± 23.2	36.9 ± 6.7	112.4 ± 15.3	NA	93.0 ± 10.5	5.6 ± 0.3	1.2 ± 1.08^c^	258 (35.3)	394 (54.4)	NA	214 (29.6)	67 (9.3)	NA
	Placebo	949	45.1 ± 11.9	341 (35.9)	103.9 ± 22.0	37.1 ± 6.3	112.8 ± 14.5	NA	92.4 ± 10.0	5.6 ± 0.3	1.2 ± 1.23^c^	369 (38.9)	539 (57.5)	NA	267 (28.5)	72 (7.7)	NA
Rosenstock et al. 2025 (ACHIEVE‐1) [27]	OFG 3 mg	143	53.3 ± 11.3	80 (56)	90.3 ± 25.7	32.9 ± 8.0	107.0 ± 16.5	55 (38.5)	142.9 ± 38.7	7.9 ± 0.9	1.6 ± 2.39^b^	57 (39.9)	37 (25.9)	35 (24.5)	63 (44.1)	8 (5.6)	4.0 ± 4.8
	OFG 12 mg	137	54.1 ± 11.8	66 (48)	90.6 ± 23.1	33.3 ± 7.8	107.7 ± 16.9	53 (38.7)	155.3 ± 55.1	7.9 ± 0.9	1.4 ± 1.17^b^	55 (40.1)	30 (21.9)	38 (27.7)	59 (43.1)	9 (6.6)	5.1 ± 6.0
	OFG 36 mg	141	52.8 ± 11.8	69 (49)	90.1 ± 22.9	33.1 ± 7.3	107.6 ± 17.0	52 (36.9)	148.8 ± 40.0	8.1 ± 0.9	1.4 ± 1.19^b^	55 (39.0)	40 (28.4)	35 (24.8)	62 (44.0)	4 (2.8)	4.2 ± 5.1
	Placebo	138	53.3 ± 12.5	75 (54)	90.0 ± 20.7	32.9 ± 6.8	106.9 ± 14.1	54 (39.1)	143.3 ± 42.2	7.9 ± 0.9	1.6 ± 1.18^b^	56 (40.6)	38 (27.5)	35 (25.4)	61 (44.2)	3 (2.2)	4.4 ± 5.6
Frias et al. 2023 [8]	OFG 3 mg	51	59.0 ± 9.4	26 (51)	99.3 ± 25.4	35.3 ± 8.2	112.9 ± 18.4	44 (86)	164.0 ± 40.9	8.0 ± 0.8	1.5 ± 1.71	7 (14)	47 (92)	0 (0)	1 (2)	2 (4)	6.6 ± 6.9
	OFG 12 mg	56	57.4 ± 9.2	36 (64)	99.3 ± 18.1	34.8 ± 6.3	113.7 ± 11.8	52 (93)	172.1 ± 42.8	8.2 ± 0.9	1.9 ± 2.17	15 (27)	49 (88)	1 (2)	1 (2)	5 (9)	7.8 ± 6.8
	OFG 24 mg	47	60.5 ± 9.1	30 (64)	98.5 ± 22.9	34.1 ± 7.7	113.2 ± 15.3	46 (98)	171.7 ± 44.4	8.2 ± 0.9	1.9 ± 2.19	5 (11)	43 (91)	1 (2)	1 (2)	2 (4)	6.4 ± 5.1
	OFG 36 mg	61	59.7 ± 9.2	36 (59)	98.9 ± 17.5	34.4 ± 5.4	112.1 ± 12.7	54 (89)	157.9 ± 28.7	8.0 ± 0.7	1.9 ± 2.19	13 (21)	58 (95)	0 (0)	1 (2)	0 (0)	6.1 ± 4.7
	OFG 45 mg	63	58.5 ± 9.4	40 (63)	104.6 ± 25.1	35.4 ± 8.0	116.0 ± 16.6	56 (89)	166.4 ± 35.0	8.1 ± 0.9	2.2 ± 2.54	13 (21)	57 (90)	1 (2)	0 (0)	5 (8)	6.8 ± 5.8
	Placebo	55	58.3 ± 9.5	28 (51)	102.0 ± 18.8	35.8 ± 6.2	115.0 ± 12.4	51 (93)	172.0 ± 42.9	8.1 ± 0.9	1.5 ± 1.78	14 (25)	50 (91)	1 (2)	0 (0)	4 (7)	8.1 ± 6.5
Wharton et al. 2023 (GZGI) [7]	OFG 12 mg	50	49.8 ± 10.5	19 (38)	107.5 ± 25.3	37.7 ± 7.7	114.4 ± 16.5	39 (78.0)	94.4 ± 9.8	5.5 ± 0.4	1.2 ± 1.41^b^	NA	47 (94)	0 (0)	0 (0)	3 (6)	NA
	OFG 24 mg	53	57.0 ± 9.1	23 (43)	112.1 ± 30.2	38.1 ± 7.7	120.1 ± 19.1	42 (79.2)	97.5 ± 12.0	5.7 ± 0.3	1.5 ± 1.55^b^	NA	46 (87)	1 (2)	0 (0)	6 (11)	NA
	OFG 36 mg	58	55.9 ± 11.3	22 (38)	108.3 ± 25.45	38.0 ± 6.3	117.3 ± 15.37	43 (74.1)	96.9 ± 13.3	5.7 ± 0.4	1.05 ± 1.08^b^	NA	25 (86)	0 (0)	0 (0)	8 (14)	NA
	OFG 45 mg	61	53.8 ± 11.91	26 (43)	108 ± 24.5	37.7 ± 6.6	116.9 ± 13.73	43 (70.5)	95.2 ± 9.7	5.7 ± 0.4	1.3 ± 1.64^b^	NA	59 (97)	0 (0)	0 (0)	1 (1.64)	NA
	Placebo	50	54.0 ± 8.8	21 (42)	107.6 ± 25.2	37.8 ± 6.5	115.5 ± 15.4	41 (82.0)	97.2 ± 10.2	5.6 ± 0.4	1.0 ± 1.41^b^	NA	45 (90)	0 (0)	2 (4)	1 (2)	NA
Pratt et al. 2023 [24]	OFG 45 mg	9	62.8 ± 4.4	4 (44.4)	81.5 ± 10.24	29.8 ± 2.8	NA	9 (100.0)	NA	7.9 ± 0.8	NA	NA	NA	NA	NA	N/A	10.4 ± 4.8
	Placebo	17	56.0 ± 6.0	10 (58.8)	90.3 ± 20.04	31.3 ± 4.9	NA	15 (88.2)	NA	8.1 ± 0.8	NA	NA	NA	NA	NA	N/A	8.6 ± 4.9
Horn et al. 2025 (ATTAIN‐2) [28]	OFG 12 mg	332	56.2 ± 10.5	177 (53.3)	102.7 ± 21.3	36.1 ± 6.3	116.2 ± 13.4	NA	155.1 ± 43.0	8.08 ± 0.76	NA	NA	235 (70.8)	2 (0.6)	55 (16.6)	19 (5.7)	6.4 (3.5–10.1)^d^
	OFG 36 mg	322	58.1 ± 10.8	168 (52.2)	99.8 ± 23.0	35.1 ± 6.5	114.7 ± 15.1	NA	154.7 ± 40.1	8.05 ± 0.73	NA	NA	228 (70.8)	1 (0.3)	54 (16.8)	28 (8.7)	7.0 (4.0–13.1)^d^
	Placebo	630	56.5 ± 10.9	332 (52.7)	101.2 ± 22.6	35.5 ± 6.5	115.0 ± 14.6	NA	151.5 ± 39.5	8.03 ± 0.75	NA	NA	442 (70.2)	2 (0.3)	112 (17.8)	37 (5.9)	6.8 (3.5–10.9)^d^

Abbreviations: AI/AN, American Indian or Alaska Native; B/AA, Black or African American; BMI, body mass index; H/L, Hispanic or Latino; HbA1c, haemoglobin; NA, not applicable; OFG, orforglipron; T2DM, type 2 diabetes mellitus; WC, waist circumference.

^a^ Plus–minus values are means ±SD.

^b^ LSM (SD); Least square mean (standard deviation).

^c^ MBE (SD); Model‐Based Estimates (standard deviation).

^d^ Median (IQR).

### 3.3 Risk of Bias Assessment and Certainty of Evidence

Overall, five RCTs were rated as having low risk of bias, while one RCT was rated as having some concerns (Figure 2). All RCTs were assessed depending on Cochrane ROB‐2 domains, including bias arising from the randomisation process, bias due to deviations from intended interventions, bias due to missing outcome data, bias in measurement of the outcome and bias in selection of the reported result. However, the source of bias is mainly related to the distribution of patient loss, which was uneven in three groups (Domain 3: bias due to missing outcome data). All included studies demonstrated a low risk of bias with respect to sequence generation and baseline comparability, reflecting adequate randomisation procedures and balanced baseline characteristics across study arms. Certainty assessment of efficacy and safety outcomes of OFG versus placebo were evaluated using a methodological framework to evaluate confidence in the results from network meta‐analyses, Confidence in Network Meta‐Analysis (CINeMA) [29]. CINeMA assessment is illustrated in Table 3.

**TABLE 3:** Certainty of evidence for efficacy and safety outcomes of OFG versus placebo using CINeMA framework.

⊕⊕⊕⊕High certainty	⊕⊕⊕⊝ Moderate certainty	⊕⊕⊝⊝ Low certainty	⊕⊝⊝⊝ Very low certainty	N/A—Not assessed

OFG doses vs placebo	Efficacy outcomes						Safety outcomes			
	Body wt 12 wk (kg)	Body wt 36 wk (kg)	Waist circ. 12 wk (cm)	Waist circ. 36 wk (cm)	HbA1c 12 wk (%)	Fasting glucose 12 wk	Any TEAE	Hypo‐glycemia	Headache	Thyroid cancer
OFG 3 mg	⊕⊕⊕⊝ Moderate	N/A	N/A	N/A	⊕⊕⊝⊝ Low	⊕⊕⊝⊝ Low	⊕⊕⊕⊝ Moderate	⊕⊕⊝⊝ Low	⊕⊕⊝⊝ Low	⊕⊕⊝⊝ Low
OFG 12 mg	⊕⊕⊕⊕ High	⊕⊕⊝⊝ Low	⊕⊕⊝⊝ Low	⊕⊕⊕⊝ Moderate	⊕⊕⊕⊝ Moderate	⊕⊝⊝⊝ Very low	⊕⊕⊝⊝ Low	⊕⊕⊕⊝ Moderate	⊕⊕⊕⊝ Moderate	⊕⊕⊕⊝ Moderate
OFG 24 mg	⊕⊕⊝⊝ Low	⊕⊕⊕⊕ High	⊕⊕⊕⊝ Moderate	⊕⊕⊕⊕ High	⊕⊕⊝⊝ Low	⊕⊕⊝⊝ Low	⊕⊕⊝⊝ Low	⊕⊕⊝⊝ Low	⊕⊕⊕⊝ Moderate	⊕⊕⊝⊝ Low
OFG 36 mg	⊕⊕⊕⊕ High	⊕⊕⊝⊝ Low	⊕⊕⊝⊝ Low	⊕⊕⊕⊝ Moderate	⊕⊕⊕⊝ Moderate	⊕⊝⊝⊝ Very low	⊕⊕⊝⊝ Low	⊕⊕⊕⊝ Moderate	⊕⊕⊕⊝ Moderate	⊕⊕⊕⊝ Moderate
OFG 45 mg	⊕⊕⊕⊝ Moderate	⊕⊕⊕⊕ High	⊕⊕⊕⊝ Moderate	⊕⊕⊕⊕ High	⊕⊕⊕⊝ Moderate	⊕⊕⊕⊝ Moderate	⊕⊕⊕⊕ High	⊕⊕⊕⊝ Moderate	⊕⊕⊝⊝ Low	⊕⊕⊝⊝ Low

Reasons for downgrading certainty of evidence.		
OFG dose	Outcome (certainty rating)	Domain(s) responsible for downgrading
OFG 3 mg	⊕⊕⊕⊝ Moderate—Body Weight 12 wk	Heterogeneity
	⊕⊕⊝⊝ Low—HbA1c 12 wk	Heterogeneity
	⊕⊕⊝⊝ Low—Fasting Glucose 12 wk	Within‐study bias, Heterogeneity
	⊕⊕⊕⊝ Moderate—Any TEAE	Imprecision
	⊕⊕⊝⊝ Low—Hypoglycemia	Imprecision
	⊕⊕⊝⊝ Low—Headache	Imprecision
	⊕⊕⊝⊝ Low—Thyroid Cancer	Imprecision
OFG 12 mg	⊕⊕⊝⊝ Low—Body Weight 36 wk	Incoherence
	⊕⊕⊝⊝ Low—Waist Circumference 12 wk	Incoherence
	⊕⊕⊕⊝ Moderate—Waist Circumference 36 wk	Heterogeneity
	⊕⊕⊕⊝ Moderate—HbA1c 12 wk	Heterogeneity
	⊕⊝⊝⊝ Very low—Fasting Glucose 12 wk	Within‐study bias, Heterogeneity, Incoherence
	⊕⊕⊝⊝ Low—Any TEAE	Heterogeneity
	⊕⊕⊕⊝ Moderate—Hypoglycemia	Imprecision, Heterogeneity
	⊕⊕⊕⊝ Moderate—Headache	Imprecision
	⊕⊕⊕⊝ Moderate—Thyroid Cancer	Imprecision
OFG 24 mg	⊕⊕⊝⊝ Low—Body Weight 12 wk	Incoherence
	⊕⊕⊕⊝ Moderate—Waist Circumference 12 wk	Heterogeneity, Incoherence
	⊕⊕⊝⊝ Low—HbA1c 12 wk	Within‐study bias, Heterogeneity
	⊕⊕⊝⊝ Low—Fasting Glucose 12 wk	Within‐study bias, Heterogeneity
	⊕⊕⊝⊝ Low—Any TEAE	Imprecision
	⊕⊕⊝⊝ Low—Hypoglycemia	Imprecision
	⊕⊕⊕⊝ Moderate—Headache	Imprecision, Heterogeneity
	⊕⊕⊝⊝ Low—Thyroid Cancer	Within‐study bias, Imprecision
OFG 36 mg	⊕⊕⊝⊝ Low—Body Weight 36 wk	Incoherence
	⊕⊕⊝⊝ Low—Waist Circumference 12 wk	Incoherence
	⊕⊕⊕⊝ Moderate—Waist Circumference 36 wk	Heterogeneity
	⊕⊕⊕⊝ Moderate—HbA1c 12 wk	Heterogeneity
	⊕⊝⊝⊝ Very low—Fasting Glucose 12 wk	Within‐study bias, Heterogeneity, Incoherence
	⊕⊕⊝⊝ Low—Any TEAE	Heterogeneity
	⊕⊕⊕⊝ Moderate—Hypoglycemia	Heterogeneity
	⊕⊕⊕⊝ Moderate—Headache	Heterogeneity
	⊕⊕⊕⊝ Moderate—Thyroid Cancer	Imprecision
OFG 45 mg	⊕⊕⊕⊝ Moderate—Body Weight 12 wk	Incoherence
	⊕⊕⊕⊝ Moderate—Waist Circumference 12 wk	Heterogeneity, Incoherence
	⊕⊕⊕⊝ Moderate—HbA1c 12 wk	Within‐study bias, Heterogeneity
	⊕⊕⊕⊝ Moderate—Fasting Glucose 12 wk	Within‐study bias, Heterogeneity
	⊕⊕⊕⊝ Moderate—Hypoglycemia	Imprecision, Heterogeneity
	⊕⊕⊝⊝ Low—Headache	Imprecision
	⊕⊕⊝⊝ Low—Thyroid Cancer	Within‐study bias, Imprecision

Abbreviations: CINeMA domains assessed, within‐study bias, reporting bias, indirectness, imprecision, heterogeneity and incoherence; HbA1c, Glycated Haemoglobin; Hypoglycemia, Hypoglycaemia with plasma glucose < 54 mg/dL; N/A, outcome not reported for this dose group; OFG, Orforglipron; TEAE, Treatment‐Emergent Adverse Events; Waist Circ., Waist Circumference; wk, weeks.

### 3.4 Outcomes

#### 3.4.1 Weight‐Related Outcomes

##### 3.4.1.1 Total Body Weight (kg)

The NMA evaluated the effects OFG on body weight changes across three follow‐up periods. Overall, OFG was consistently superior to placebo in reducing total body weight. This outcome was assessed in six studies over 12 weeks (4615 patients; five doses), two studies over 26 weeks (520 patients; five doses) and three studies over 36 weeks (3331 patients; four doses).

All OFG doses were associated with a significant reduction in total body weight at weeks 12, 26 and 36.

**FIGURE 3:**
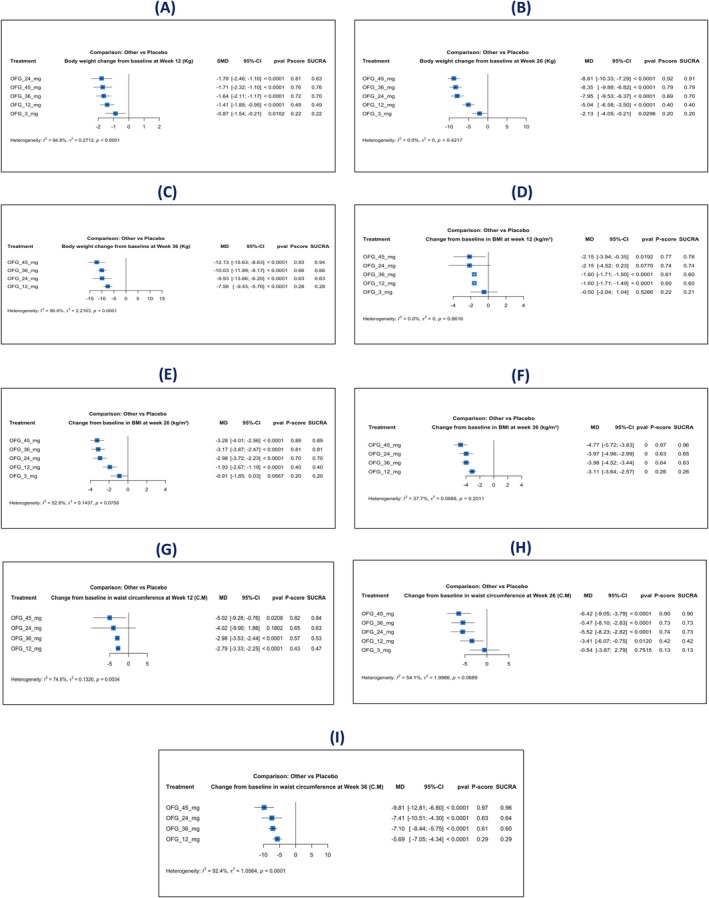
Forest plots for change from baseline in weight‐related outcomes at different follow‐ups: (A) body weight change from baseline at week 12, (B) body weight change from baseline at week 26, (C) body weight change from baseline at week 36, (D) change from baseline in BMI at week 12, (E) change from baseline in BMI at week 26, (F) change from baseline in BMI at week 36, (G) change from baseline in waist circumference at week 12, (H) change from baseline in waist circumference at week 26, (I) change from baseline in waist circumference at week 36. BMI, body mass index; cm, centimetres; kg, kilograms; kg/m^2^, kilograms per square metre.

**FIGURE 4:**
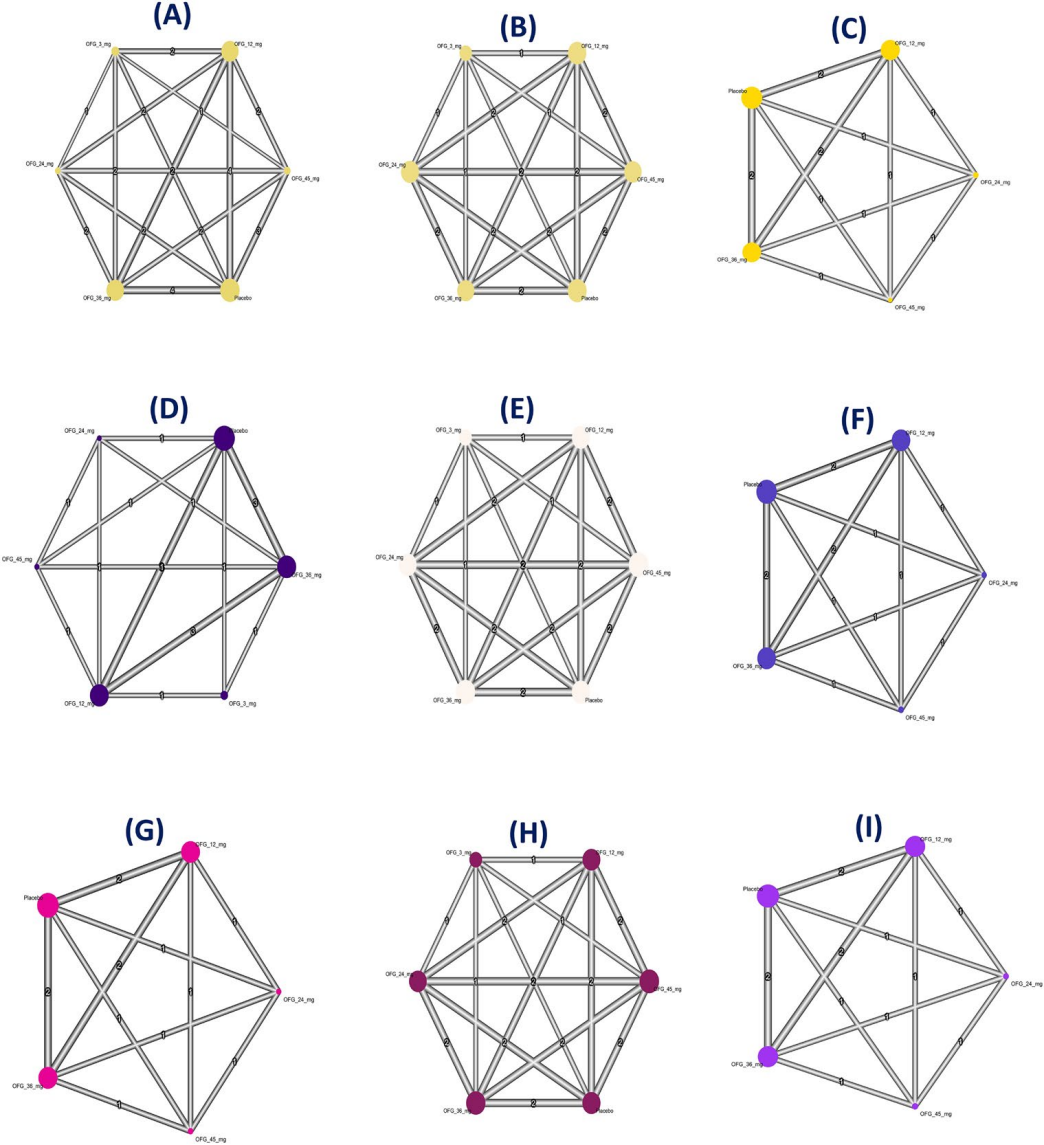
Network graphs for change from baseline in hemodynamic parameters at different follow‐ups: (A) body weight change from baseline at week 12, (B) body weight change from baseline at week 26, (C) body weight change from baseline at week 36, (D) change from baseline in BMI at week 12, (E) change from baseline in BMI at week 26, (F) change from baseline in BMI at week 36, (G) change from baseline in waist circumference at week 12, (H) change from baseline in waist circumference at week 26, (I) change from baseline in waist circumference at week 36. BMI, body mass index; cm, centimetres; kg, kilograms; kg/m^2^, kilograms per square metre.

At week 12, the 24‐mg dose (SMD: −1.78; 95% CI −2.46 to −1.10; *p* < 0.0001; P‐score = 0.81; SUCRA: 0.83) and 45‐mg dose (SMD: −1.71; 95% CI −2.32 to −1.10; *p* < 0.0001; P‐score = 0.76; SUCRA: 0.76) produced the greatest reduction compared with placebo. Heterogeneity was high (*I* ^2^ = 94.8%; *p* < 0.0001) (Figure 3A). At 26 weeks, the 45 mg dose again produced the largest effect (MD: −8.81; 95% CI: [−10.33 to −7.29], *p* < 0.0001; P‐score = 0.92; SUCRA: 0.91), followed by the 36 mg dose (MD:8.35; 95% CI: [−9.88; −6.82], *p* < 0.0001; P‐score = 0.79; SUCRA: 0.79). No Heterogeneity was detected (*I* ^2^ = 0%, *p* = 0.42) (Figure 3B). At 36 weeks, compared with placebo, the 45 mg dose produced the greatest reduction in body weight (MD: −12.13; 95% CI: [−15.63 to −8.63]; *p* < 0.0001; P‐score = 0.93; SUCRA: 0.94), followed by the 36 mg dose (MD: −10.03; 95% CI −11.89 to −8.17; *p* < 0.0001; P‐score = 0.66; SUCRA: 0.66). Heterogeneity was significant (*I* ^2^ = 96.8%, *p* < 0.0001) (Figure 3C). A network graph summarising all direct comparisons between OFG doses in our study regarding total body weight changes at three different follow‐ups (12, 26 and 36 weeks) is illustrated in (Figure 4A–C).

###### 3.4.1.1.1 Subgroup Analysis by Diabetes Status—Body Weight (kg)

Subgroup analyses compared the treatment effects of OFG dose groups (12, 24, 36 and 45 mg) versus placebo according to diabetes status (participants with and without diabetes). At 12 and 26 weeks, no statistically significant differences were observed between participants with and without diabetes across all dose comparisons (all *p* > 0.05) (Figures S6 and S7). At 36 weeks, significant subgroup differences emerged. Greater reductions in total body weight were observed among participants without diabetes for both the 12‐mg dose versus placebo (*Q* = 22.54, *p* < 0.0001) and the 36‐mg dose versus placebo (*Q* = 23.33; *p* < 0.0001) (Figure S8).

##### 3.4.1.2 Body Mass Index (kg/m^2^)

The network meta‐analysis evaluated the effects of OFG on BMI across three follow‐up periods. This outcome was assessed in three studies over 12 weeks (3068 patients) and in two studies each at 26 weeks (589 participants) and 36 weeks (2263 participants). At 12 weeks, compared with placebo, the 45, 36 and 12 mg doses produced significant reductions in BMI. The greatest effect was observed with the 45‐mg dose (MD −2.15; 95% CI −3.94 to −0.35; *p* = 0.01; P‐score = 0.77; SUCRA = 0.78). The 24 mg dose and 3 mg dose demonstrated a nonsignificant trend toward BMI reduction (*p* = 0.05). No heterogeneity was detected (*I* ^2^ = 0.0%, *p* = 0.86) (Figure 3D). At 26 weeks, OFG demonstrated a dose‐responsive trend, with all doses from 12 to 45 mg producing significant reductions in BMI relative to placebo. The largest decrease occurred with the 45‐mg dose (MD −3.28; 95% CI −4.01 to −2.56; *p* < 0.0001; P‐score = 0.88; SUCRA = 0.89), followed by the 36‐mg dose (MD −3.17; 95% CI −3.87 to −2.47; *p* < 0.0001; P‐score = 0.81; SUCRA = 0.81). Moderate heterogeneity was observed (*I* ^2^ = 52.8%, *p* = 0.07) (Figure 3E). Similarly, at 36 weeks, OFG showed the dose response relationship with statistically significant effect for the 45–12 mg doses, with higher effect for the 45 mg dose (MD −4.77; 95% CI: −5.72 to −3.83; *p* < 0.0001; P‐score = 0.97; SUCRA = 0.96) (Figure 3F). A network graph summarising all direct comparisons between OFG doses in our study regarding BMI changes at three different follow‐ups (12, 26 and 36 weeks) is illustrated in (Figure 4D–F).

###### 3.4.1.2.1 Subgroup Analysis by Diabetes Status—BMI (kg/m^2^)

Subgroup analyses comparing treatment effects of OFG dose groups (12, 36 and 45 mg) and placebo according to the diabetes status (patients with or without diabetes) at both 12 and 26 weeks. BMI reductions were similar between participants regardless of diabetes status, with no significant subgroup interactions for most OFG doses; all dose‐to‐dose and most dose‐to‐placebo comparisons showed consistent effects across diabetes subgroups (Figures S9 and S10).

##### 3.4.1.3 Waist Circumference (cm)

The network meta‐analysis evaluated the effects of OFG on WC across three follow‐up periods. This outcome was assessed in three studies at 12 weeks (3756 patients; four doses), two studies at 26 weeks (589 patients; five doses) and three studies at 36 weeks (3372 patients; four doses).

At 12 weeks, compared with placebo, the 45, 36 and 12 mg doses produced statistically significant reductions in WC, with the greatest effect observed at 45 mg (MD −5.02; 95% CI −9.28 to −0.76; *p* = 0.02; P‐score = 0.82; SUCRA = 0.84). The 24 mg dose showed a nonsignificant trend toward improvement (MD −4.02; *p* = 0.18). A significant heterogeneity was detected (*I* ^2^ = 74.6%, *p* = 0.003) (Figure 3G). At 26 weeks, all OFG doses, except the 3 mg, produced a significant reduction in WC. The largest effect was observed with 45 (MD −6.42; 95% CI −9.05 to −3.97; *p* < 0.0001; P‐score = 0.90; SUCRA = 0.90), followed by 36 mg (MD −5.47), 24 mg (MD −5.52) and 12 mg (MD −3.41). Moderate heterogeneity was observed (*I* ^2^ = 54.1%, *p* = 0.06) (Figure 3H). At 36 weeks, all OFG doses demonstrated statistically significant reductions in WC. The magnitude of reduction was greatest at 45 mg (MD −9.81; 95% CI −12.81 to −6.80; *p* < 0.0001; P‐score = 0.97; SUCRA = 0.96), followed by 24 mg (MD −7.41), 36 mg (MD −7.10) and 12 mg (MD −5.69); all *p* < 0.0001. A significant heterogeneity was observed (*I* ^2^ = 92.4%, *p* < 0.0001) (Figure 3I). A network graph summarising all direct comparisons between OFG doses in our study regarding WC changes at three different follow‐ups (12, 26 and 36 weeks) is illustrated in (Figure 4G–I).

###### 3.4.1.3.1 Subgroup Analysis by Diabetes Status—Waist Circumference (cm)

Subgroup analyses comparing treatment effects of OFG dose groups (12 mg, 36 mg and 45 mg) and placebo according to the diabetes status (patients with or without diabetes) (Figures S11–S13). At 12 and 36 weeks, significant subgroup differences emerged. Greater reductions in waist circumference were observed among participants without diabetes for both the 36‐mg dose versus placebo (at 12 weeks: *Q* = 4.97; *p* = 0.0258; at 36 weeks: *Q* = 35.52; *p* < 0.0001) and the 12‐mg dose versus placebo (at 12 weeks: *Q* = 15.13; *p* = 0.0001; *Q* = 31.52; *p* < 0.0001).

##### 3.4.1.4 Categorical Weight Loss Threshold

The network meta‐analysis evaluated the effects of OFG on categorical weight‐loss outcomes, defined as achieving > 5%, > 10%, and > 15% total weight loss at 26 weeks. This analysis included two studies, involving 1389 participants.

At week 26, participants receiving OFG 24–45 mg were 18–20 times more likely to achieve > 5% weight loss compared with placebo with largest reduction observed at 36 mg (OR 20.21, 95% CI 9.24 to 44.20, *p* < 0.0001, P‐score = 0.83; SURCA = 0.83), followed by 24 mg (OR 19.29) and 45 mg (OR 18.01) with no observed heterogeneity (*I* ^2^ = 0.0%, *p* = 0.57) (Figure S1A). Similar improvements were observed for > 10% weight loss threshold (ORs 12.1–16.0) with the highest probabilities associated with 45 mg (OR 15.98, 95% CI 4.30–59.33, *p* < 0.0001, P‐score = 0.84; SUCRA = 0.84). Moderate heterogeneity was observed (*I* ^2^ = 54.1%, *p* = 0.06) (Figure S1B). Additionally, OFG significantly increased the likelihood of achieving > 15% weight loss, with ORs ranging from 8.6 to 11.2. The greatest effect occurred with the 45‐mg dose (OR 11.15; 95% CI 2.06–60.32; *p* = 0.005; P‐score = 0.84; SUCRA = 0.83). Moderate heterogeneity was observed (*I* ^2^ = 40.4, *p* = 0.15) (Figure S1C). A network graph summarising all direct comparisons between OFG doses in our study regarding > 5%, > 10% and > 15% weight loss threshold at 26 weeks is illustrated in Figure S2.

###### 3.4.1.4.1 Subgroup Analysis by Diabetes Status—Categorical Weight Loss

Subgroup analyses comparing treatment effects of OFG dose groups (12, 36 and 45 mg) and placebo according to the diabetes status (patients with or without diabetes) at 26 weeks showed consistent treatment effects across participants with and without diabetes. All OFG doses produced higher odds of achieving ≥ 5%, ≥ 10% and ≥ 15% weight loss compared with placebo in both subgroups, with absolute effect sizes generally larger among non‐diabetic participants. However, tests for interaction showed no significant subgroup heterogeneity for nearly all dose‐to‐dose or dose‐to‐placebo comparisons (all *p* > 0.05). The only exception was the comparison of OFG 12 mg versus OFG 24 mg for the ≥ 15% weight‐loss outcome (*Q* = 4.33, *p* = 0.037), although this difference is unlikely to be clinically meaningful. Overall, these findings support comparable relative efficacy of OFG across diabetes subgroups (Figures S14–S16).

#### 3.4.2 Glycemic Outcome

The network meta‐analysis assessed the effect of OFG on glycemia control at 12 weeks, including five dosages. Four studies, including 2016 patients, reported changes in HbA1C (%), while three studies assessed FBG.

**FIGURE 5:**
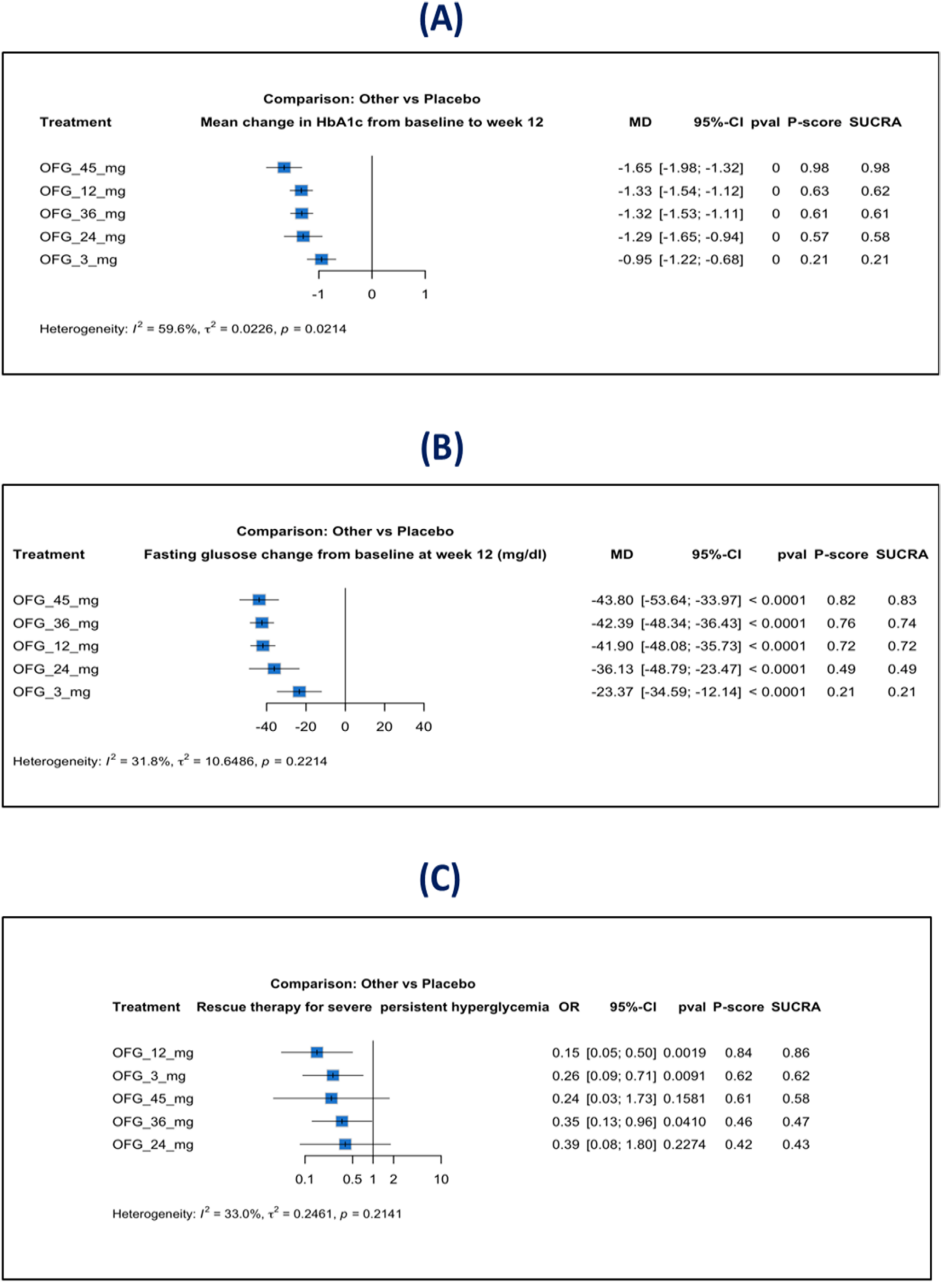
Forest plots for change from baseline in glycemic‐related outcomes and additional related outcomes: (A) mean change in HbA1c (%) from baseline to week 12, (B) fasting glucose change from baseline at week 12, (C) treatment rescue therapy for severe persistent hyperglycemia.

All OFG doses were associated with improvements in glycemic parameters relative to placebo. Reductions in HbA1c were observed across all dose levels by week 12, with the greatest decrease seen at 45 mg (MD −1.65; 95% CI −1.98 to −1.32; *p* < 0.0001; P‐score = 0.98; SUCRA = 0.98). This was followed by the 12 mg (MD −1.33), 36 mg (MD −1.32) and 24 mg doses (MD −1.29). Heterogeneity was detected (*I* ^2^ = 59.6%, *p* = 0.02) (Figure 5A). Changes in FBG (mg/dL) followed a similar pattern. The largest improvement occurred at the 45‐mg dose (MD −43.80, 95% CI −53.64 to −33.97, *p* < 0.0001, P‐score = 0.82; SUCRA: 0.83), followed closely by the 36‐mg dose (MD −42.39, 95% CI: −48.34; −36.43, *p* < 0.0001, P‐score = 0.76; SUCRA: 0.74). Heterogeneity was not significant (*I* ^2^ = 31.8%, *p* = 0.2214) (Figure 5B).

Rescue therapy for persistent hyperglycemia was infrequent. The lowest likelihood of requiring rescue therapy was observed with the 12 mg dose (OR 0.15; 95% CI 0.05–0.50; *p* = 0.001; P‐score = 0.84; SUCRA = 0.86), followed by the 3 and 36‐mg doses. Heterogeneity for this outcome was not significant (*I* ^2^ = 33%, *p* = 0.21) (Figure 5C). A network graph summarising all direct comparisons between OFG doses in our study, change in HbA1C (%), fasting blood glucose (mg/dl) and rescue therapy for persistent hyperglycemia at 12 weeks is illustrated in Figure S3.

#### 3.4.3 Safety Outcomes

##### 3.4.3.1 Treatment Emergent Adverse Effect

The NMA evaluated the incidence of treatment‐emergent adverse events across six studies involving 4883 patients. However, the 45‐mg dose demonstrated the highest likelihood of TEAEs compared with placebo (OR 2.45; 95% CI 1.37–4.38; *p* = 0.002; P‐score = 0.05; SUCRA = 0.04), followed by the 12 mg dose (OR 1.48; *p* < 0.0026) and the 36 mg dose (OR 1.37; *p* = 0.0142). The 24 mg dose exhibited a trend toward increased TEAEs (OR 1.69; 95% CI 0.93–3.09) but did not reach statistical significance (*p* = 0.085). No significant heterogeneity was observed across studies (*I* ^2^ = 18.8%, *p* = 0.25) (Figure S4A).

Subgroup analysis based on diabetes status demonstrated no statistically significant differences in TEAE incidence between participants with and without diabetes (all *p* > 0.05) (Figure S17).

##### 3.4.3.2 Hypoglycemia

The NMA evaluated the incidence of hypoglycemia across three studies, including 852 patients. Hypoglycemia was rare and showed no significant difference compared to placebo across all OFG doses. However, the hypoglycemia was significant at the 36 mg dose (OR: 3.90, 95% CI: 1.22 to 12.47, *p* = 0.0217, P‐score = 0.34; SUCRA: 0.34). Heterogeneity was absent (*I* ^2^ = 0.0%, *p* = 0.51) (Figure S4B).

##### 3.4.3.3 Other Adverse Events

Headache incidence was assessed across three studies, including 3231 patients, with 274 events recorded. Headaches occurred at similar frequencies across all treatment doses compared to the placebo. However, the headaches were significantly associated with the 36 mg dose (OR: 1.33, 95% CI: 1.01 to 1.75, *p* = 0.0426, P‐score = 0.45; SUCRA: 0.45) (Figure S4C). Subgroup analysis by diabetes status for headache was demonstrated in Figure S18. The incidence of thyroid cancer was very low across all included studies, with no dose group demonstrating an increased risk (all *p* > 0.05) (Figure S4D). Subgroup analysis by diabetes status for thyroid cancer is shown in Figure S19. However, network graphs for all safety outcomes are illustrated in Figure S5.

### 3.5 Inconsistency

A design‐by‐treatment analysis did not reveal any significant global inconsistency (all *p* > 0.05) for body weight change from baseline at week 26 and 36 (Figures S21 and S22), change from baseline in BMI at week 12, 26 and 36 (Figures S23–S25), change from baseline in WC at week 12, 26 and 36 (Figures S26–S28), participants achieving ≥ 5%, ≥ 10% and ≥ 15% weight loss at week 26 (Figures S29–S31), any TEAE (Figure S32), hypoglycemia with plasma glucose < 54 mg/dL (Figure S33), headache (Figure S34), thyroid cancer (Figure S35) and fasting glucose change from baseline at week 12 (Figure S36). However, global inconsistency was significant for body weight change from baseline at week 12 (*p* < 0.0001) (Figure S20), and mean change in HbA1c from baseline to week 12 (Figure S37). The node‐splitting method revealed no significant inconsistencies across most outcomes (*p* > 0.05), including rescue therapy for severe, persistent hyperglycemia (Figure S38). However, significant inconsistency was observed for body weight change from baseline at week 12 in most of the comparisons (*p* < 0.05). Additionally, the mean change in HbA1c from baseline to week 12 showed significant inconsistency in comparison of OFG 3 mg vs. OFG 45 mg (*p* = 0.0208). Similarly, fasting glucose change from baseline at week 12 demonstrated significant inconsistency for OFG 12 mg vs. placebo (*p* = 0.0473), OFG 24 mg vs. OFG 45 mg (*p* = 0.0481), OFG 3 mg vs. OFG 45 mg (*p* = 0.0481) and OFG 36 mg vs. placebo (*p* = 0.0486).

### 3.6 Sensitivity Analysis

For body weight change from baseline at week 12, the findings remained consistent with the primary analysis after excluding small‐sample studies (Figure S39) and phase I and II studies (Figure S40). The results for BMI change from baseline at week 12 were similarly robust after excluding phase I and II studies (Figure S41). For mean HbA1c change from baseline to week 12, the results remained stable after exclusion of a small‐sample study (Figure S42). Regarding any TEAE, the findings were consistent with the primary analysis after excluding small‐sample studies (Figure S43) and after excluding phase I and II studies (Figure S44). The results for thyroid cancer and headache outcomes also remained stable after exclusion of phase I and II studies (Figures S45 and S46, respectively).

## 4 Discussion

In adults with or without T2DM, the clinical aim is to achieve weight reduction that is large enough to improve cardiometabolic risk while remaining tolerable for sustained use. OFG is an oral GLP‐1 RA evaluated across several dose levels in RCTs. In this NMA of six RCTs, OFG demonstrated consistent benefits versus placebo across weight‐related and glycemic outcomes, and effects generally increased with dose [5, 7, 8, 24, 27, 28].

Total body weight reduction was the most consistent signal and provides the clearest clinical anchor. Across follow‐up periods, higher dose regimens, particularly 24 mg and above, produced the largest reductions, while lower doses produced smaller effects and showed more uncertainty at later follow‐up. This gradient is clinically relevant because weight loss benefits are typically proportional to the amount lost [30]. A reduction of at least 5% is often associated with improvements in blood pressure, glycemic measures and lipid parameters, while larger reductions are more likely to improve fatty liver disease, sleep apnea severity, mobility and health‐related quality of life [31, 32, 33]. Accordingly, the efficacy of OFG doses has direct implications for counselling on expected benefit and for selecting a dose that aligns with patient priorities and comorbidity burden.

BMI and WC changes were directionally concordant with total body weight, but their time course was less consistent. BMI reductions were numerically favourable at 12 weeks and became more clearly separated from placebo at 26 and 36 weeks for doses of 12 mg and above. The WC showed limited early separation and then improved later, primarily at higher doses. This pattern is biologically plausible because central adiposity reduction may lag behind early weight change, and because waist measurement is more sensitive to technique, body shape and short‐term variability in abdominal contents [34, 35]. However, later separation is meaningful from a clinical perspective because WC is a proxy for central adiposity, which correlates with insulin resistance and cardiometabolic risk, so sustained reductions support a plausible pathway to longer‐term risk improvement [36, 37].

Categorical weight loss thresholds offered a clinically interpretable complement to continuous outcomes. The strong dose response for achieving more than 5%, more than 10% and more than 15% weight loss matters because these thresholds are widely used to communicate expected benefit and to guide shared decision making. Moving from modest to larger weight loss can shift the clinical trajectory of obesity‐related disease, including improvement in glycemic control, hepatic steatosis, symptoms and severity of obstructive sleep apnea, and functional limitations [38, 39]. The very large odds ratios observed for higher thresholds at later follow‐up also highlight that, for patients who can tolerate higher doses, the probability of achieving clinically transformative weight loss may be substantially higher than with placebo. Compared with metabolic and bariatric surgery, which typically produces substantially larger and durable weight loss (around 30% total weight loss at 1 year in registry data), OFG achieved more modest mean reductions despite clear dose‐responsive efficacy [40].

Subgroup analyses by diabetes status suggested similar relative weight‐loss efficacy in participants with and without diabetes, although several contrasts indicated numerically larger effects among participants without diabetes, and confidence intervals were wide. This pattern is consistent with the broader incretin literature, where absolute weight loss can be attenuated in T2DM [41]. Plausible contributors include longer disease duration, higher baseline insulin resistance and the metabolic effects of concomitant glucose‐lowering therapies, which may influence energy balance and weight trajectories [42, 43]. From a clinical perspective, the key implication is not that therapy should differ categorically by diabetes status, but that expectations for absolute weight loss may need tailoring and that complementary management of diabetes medications remains important to reduce competing weight promoting effects.

Glycemic outcomes improved across doses and complemented the anthropometric findings. Haemoglobin A1c reductions favoured OFG at all dose levels and tended to be larger at higher doses, while fasting glucose showed the same direction with more variable early effects. Clinically, these outcomes matter in both diabetes and non‐diabetes populations. In established T2DM, sustained haemoglobin A1c improvement reduces microvascular risk and may allow simplification or deintensification of concomitant therapies in selected patients, particularly those associated with hypoglycemia or weight gain [44, 45]. In obesity without diabetes, modest improvements in glycemic measures may reduce progression risk in those with prediabetes, and may offer short‐term metabolic benefits even when baseline glycemia is near normal [46].

Safety and tolerability should be interpreted as part of the dose selection problem rather than as an isolated endpoint. Treatment‐emergent adverse events increased with dose, with the clearest increase at the highest dose and more modest or uncertain increases at intermediate doses. This pattern is clinically expected for GLP‐1 receptor agonism and is likely driven by gastrointestinal symptoms that are most frequent during initiation and escalation [47, 48]. In real‐world settings, early discontinuation due to intolerance can negate anticipated benefit, so the most clinically effective dose may be the highest dose a patient can persist with over time [49].

Hypoglycemia was rare and did not increase meaningfully with OFG at any dose. This is clinically reassuring and biologically plausible, as GLP‐1 receptor agonism enhances insulin secretion in a glucose‐dependent manner and does not typically lower glucose below physiologic thresholds in the absence of other hypoglycemia‐inducing therapies [50, 51, 52]. Headache occurred at similar frequencies across groups. Thyroid cancer events were very rare with no apparent signal, but limited duration and sample size mean that rare outcomes cannot be excluded. Clinically, the appropriate interpretation is that short‐term randomised evidence supports a reassuring profile for hypoglycemia and common non‐gastrointestinal events, while long‐term pharmacovigilance and larger outcome studies remain necessary for rare harms.

When placed in the context of the wider literature, our findings are directionally consistent with broader evidence by Ismaiel et al. and Wong et al. that GLP‐1 RAs improve weight and glycemic outcomes in obesity and diabetes, with GI adverse effects as the dominant tolerability limitation [53, 54]. Evidence mapping and umbrella syntheses across multiple outcomes report substantial benefits for weight and glycemic measures alongside predictable class‐related harms [55]. OFG adds a potential oral option within this therapeutic class, which may be clinically relevant for patients who decline injections or for whom injection‐related barriers reduce uptake. However, oral administration alone does not guarantee better persistence, and the tolerability profile suggests that the same implementation principles used for other GLP‐1‐based therapies will remain important.

Guideline context helps frame clinical implications. Current guidance emphasises lifestyle intervention as foundational and recommends anti‐obesity pharmacotherapy for adults who meet BMI thresholds or have weight‐related complications when lifestyle efforts alone are insufficient [13, 56]. Diabetes standards similarly recommend choosing glucose‐lowering therapies that promote weight loss in patients with overweight or obesity, and they increasingly prioritise incretin‐based therapies when appropriate [42]. Within this context, our findings support considering OFG as a dose titratable option with potentially large weight and glycemic benefits, while acknowledging that positioning within treatment algorithms will depend on longer duration evidence, comparative effectiveness against established agents and real‐world persistence.

The current findings reveal that OFG elicits potential reductions in body weight, BMI, WC and glycemic indices, accompanied by a good safety profile. These findings align with the effects of established incretin‐based therapies, like injectable GLP‐1 receptor agonists, such as semaglutide [57, 58, 59] and dual incretin therapy, such as tirzepatide [60, 61], both of which are actively recommended in the current management of obesity and T2DM. Notably, OFG has become the first FDA‐approved oral GLP‐1 RAs (April 2026), which may overcome adherence issues with injectable therapy but without a significant difference in the metabolic effect. In this context, the magnitude of weight loss (up to ~13 kg at 36 weeks) as well as HbA1c reduction (~1.6%) were observed, which can indicate its possible role in the treatment algorithms as an early oral incretin‐based therapy. However, these results can be used in clinical decision‐making to make the case that OFG is a convenient, effective and scalable alternative to the GLP‐1 class, which has implications in efficacy‐driven selection, enhanced tolerability‐driven adherence and increased access to the real‐world management of obesity and diabetes.

### 4.1 Strengths and Limitations

Several strengths support confidence in the synthesis. We restricted evidence to RCTs, evaluated multiple clinically relevant outcomes and used a network framework to compare several dose levels when direct head‐to‐head dose comparisons were limited. Besides, the use of the CINeMA framework to systematically assess the certainty of evidence across all outcomes. This approach enhances transparency and provides a structured evaluation of confidence in the NMA estimates. Nonetheless, limitations should temper inference. The network included only six trials and was largely anchored on placebo, which constrains comparative statements versus other active agents. Heterogeneity for several outcomes was moderate to high; although the pooled sample was sizable, the disproportionately large Wharton et al. 2025 (ATTAIN‐1 trial, *n* = 2404 participants) [5] may have amplified this and influenced several network estimates, including wide CIs. Moreover, all included trials were sponsored by the manufacturer of OFG, which may introduce publication and reporting bias. We attempted to mitigate this by exhaustive searching, including trial registries and conference abstracts, but we cannot exclude the possibility of unpublished negative studies. This analysis relied partly on reconstructed individual data using a meta‐accelerator approach. In addition, follow‐up duration was relatively short, limiting assessment of long‐term cancer outcomes. As a result, future work should extend follow‐up to evaluate the durability of weight loss, weight regain after discontinuation and long‐term safety. Trials should also report patient‐centred outcomes, including quality of life and treatment satisfaction, because these factors often drive persistence and therefore realised clinical benefit. Some of the data were digitised from a graphical representation of the data using WebPlotDigitizer. There is a possibility of minor differences between the digitised and original values, although these are minimised by independent extraction and verification. Meta‐regression could not be conducted because the number of included studies was insufficient to support reliable analyses. Furthermore, a formal assessment of publication bias was not performed due to the limited number of included RCTs (< 10), which does not allow reliable use of funnel plots or statistical tests; therefore, the possibility of small‐study effects or selective publication cannot be fully excluded and should be considered when interpreting the results.

### 4.2 Clinical Implications and Future Directions

Clinically, our results support the potential of OFG as a potent oral incretin‐based therapeutic agent for obesity and T2DM with clinically relevant changes in body weight, central adiposity and glycemic control that are generally comparable to those of established GLP‐1‐based therapeutic agents. These findings imply that larger doses can be used in patients who need significant weight loss or tighter glycemic control, whereas smaller doses can be more suitable in patients where tolerability is paramount. Importantly, the oral mode of delivery may improve treatment uptake and access compared to injectable agents, which could expand the use of incretin therapy at an early stage of obesity and diabetes treatment or for patients that do not like injections. However, the observed increase in gastrointestinal side effects highlights the need for a dose individualised, dose escalation schedule and dose‐long adherence plans to reach a long‐term benefit in clinical practice. Future research should take into account long‐term cardiovascular and metabolic effects and head‐to‐head comparisons with already approved GLP‐1 RAs and dual incretin therapy, as well as real‐world studies regarding persistence, quality of life and cost‐effectiveness. Moreover, trials with longer follow‐up are required to determine weight maintenance following discontinuation and enhance a more accurate definition of the long‐term safety profile, especially rare or delayed adverse events. Importantly, these findings could be used in future clinical guidelines, supporting the use of oral incretins as therapy options for obesity and T2DM, including OFG as a therapeutic approach.

## 5 Conclusion

In this NMA of six RCTs in adults with or without T2DM, OFG was consistently superior to placebo for reducing body weight, especially with higher doses. BMI and WC reductions aligned with weight loss and became more evident at later follow‐up. Higher doses increased the probability of achieving clinically meaningful categorical weight loss thresholds. Glycemic outcomes improved across doses, and the need for rescue therapy was uncommon, supporting a consistent metabolic effect. Treatment‐emergent adverse events increased with dose, consistent with expected class tolerability, while hypoglycemia and other non‐GI adverse events were uncommon in the available evidence. Longer trials remain necessary to address our findings at long‐term endpoints.

## Author Contributions

**Ahmed W. Hageen:** conceptualization, data curation, formal analysis, investigation, methodology, project administration, resources, software, supervision, idea validation, visualization, writing – original draft, writing – review and editing. **Ahmed Farid Gadelmawla:** writing – original draft, writing – review and editing. **Ahmad Omar Saleh:** formal analysis and interpreted the results. **Abdallfatah Abdallfatah, and Ahmed W. Hageen:** searched the databases and screened the retrieved records, writing – review and editing. **Mohamed Reyad Mohamed, Amira Fahmy El‐Nemr, Odai Maihoub, and Ahmed Elsekhary:** extracted relevant data. **Mohamed Reyad Mohamed and Hind Abdulhay:** assessed the quality of evidence, and **Ahmed W. Hageen:** resolved the conflicts. **Safir Eladawi:** designed the research workflow, writing – review and editing. **Mustafa Turkmani:** project revision. **Basel Abdelazeem and Gregg C. Fonarow:** supervised the project. All authors have read and agreed to the final version of the manuscript.

## Funding

The authors have nothing to report.

## Ethics Statement

The authors have nothing to report.

## Consent

The authors have nothing to report.

## Conflicts of Interest

Dr. Fonarow reports consulting for Abbott, Amgen, AstraZeneca, Bayer, Boehringer Ingelheim, Cytokinetics, Eli Lilly, Johnson & Johnson, Medtronic, Merck, Novartis and Pfizer. The other authors declare no conflicts of interest.

## Supporting information


**Table S1:** Detailed search strategy for each database.
**Table S2:** Preferred Reporting Items for Systematic Reviews and Meta‐Analysis (PRISMA) checklist.
**Figure S1:** Forest plots of categorical weight‐loss thresholds at week 26: (A) participants achieving ≥ 5% weight loss; (B) participants achieving ≥ 10% weight loss; (C) participants achieving ≥ 15% weight loss. Odds ratios (ORs) with 95% confidence intervals (CIs) are shown.
**Figure S2:** Network graphs for categorical weight‐loss thresholds at week 26: (A) participants achieving ≥ 5% weight loss; (B) participants achieving ≥ 10% weight loss; (C) participants achieving ≥ 15% weight loss.
**Figure S3:** Network graphs for glycemic outcomes: (A) mean change in HbA1c from baseline to week 12; (B) change in fasting glucose from baseline at week 12 (mg/dL); (C) rescue therapy for severe, persistent hyperglycemia.
**Figure S4:** Forest plot for safety outcomes: (A) any TEAE; (B) hypoglycaemia with plasma glucose < 54 mg/dL; (C) headache; (D) thyroid cancer. Odds ratios (ORs) with 95% confidence intervals (CIs) are shown.
**Figure S5:** Network graphs for safety outcomes: (A) any TEAE; (B) hypoglycaemia with plasma glucose < 54 mg/dL; (C) headache; (D) thyroid cancer.
**Figure S6:** Subgroup analysis by diabetes status for total body weight change from baseline at week 12.
**Figure S7:** Subgroup analysis by diabetes status for total body weight change from baseline at week 26.
**Figure S8:** Subgroup analysis by diabetes status for total body weight change from baseline at week 36.
**Figure S9:** Subgroup analysis by diabetes status for change from baseline in body mass index (kg/m^2^) at week 12.
**Figure S10:** Subgroup analysis by diabetes status for change from baseline in body mass index (kg/m^2^) at week 26.
**Figure S11:** Subgroup analysis by diabetes status for change from baseline in waist circumference (cm) at week 12.
**Figure S12:** Subgroup analysis by diabetes status for change from baseline in waist circumference (cm) at week 26.
**Figure S13:** Subgroup analysis by diabetes status for change from baseline in waist circumference (cm) at week 36.
**Figure S14:** Subgroup analysis by diabetes status for categorical weight loss at week 26: participants achieving ≥ 5% weight loss.
**Figure S15:** Subgroup analysis by diabetes status for categorical weight loss at week 26: participants achieving ≥ 10% weight loss.
**Figure S16:** Subgroup analysis by diabetes status for categorical weight loss at week 26: participants achieving ≥ 15% weight loss.
**Figure S17:** Subgroup analysis by diabetes status for any treatment‐emergent adverse event (TEAE).
**Figure S18:** Subgroup analysis by diabetes status for headache.
**Figure S19:** Subgroup analysis by diabetes status for thyroid cancer.
**Figure S20:** Side‐splitting analysis for body weight change from baseline at week 12.
**Figure S21:** Side‐splitting analysis for body weight change from baseline at week 26.
**Figure S22:** Side‐splitting analysis for body weight change from baseline at week 36.
**Figure S23:** Side‐splitting analysis for change from baseline in body mass index (kg/m^2^) at week 12.
**Figure S24:** Side‐splitting analysis for change from baseline in body mass index (kg/m^2^) at week 26.
**Figure S25:** Side‐splitting analysis for change from baseline in body mass index (kg/m^2^) at week 36.
**Figure S26:** Side‐splitting analysis for change from baseline in waist circumference (cm) at week 12.
**Figure S27:** Side‐splitting analysis for change from baseline in waist circumference (cm) at week 26.
**Figure S28:** Side‐splitting analysis for change from baseline in waist circumference (cm) at week 36.
**Figure S29:** Side‐splitting analysis for participants achieving ≥ 5% weight loss.
**Figure S30:** Side‐splitting analysis for participants achieving ≥ 10% weight loss.
**Figure S31:** Side‐splitting analysis for participants achieving ≥ 15% weight loss.
**Figure S32:** Side‐splitting analysis for any TEAE.
**Figure S33:** Side‐splitting analysis for hypoglycaemia with plasma glucose < 54 mg/dL.
**Figure S34:** Side‐splitting analysis for headache.
**Figure S35:** Side‐splitting analysis for thyroid cancer.
**Figure S36:** Side‐splitting analysis for change in fasting glucose (mg/dL) from baseline at week 12.
**Figure S37:** Side‐splitting analysis for mean change in HbA1c from baseline to week 12.
**Figure S38:** Side‐splitting analysis for rescue therapy for severe, persistent hyperglycemia.
**Figure S39:** Sensitivity analysis for body weight (kg) change from baseline at week 12 after excluding small‐sample studies.
**Figure S40:** Sensitivity analysis for body weight (kg) change from baseline at week 12 after excluding phase I and II studies.
**Figure S41:** Sensitivity analysis for BMI change from baseline at week 12 after excluding phase I and II studies.
**Figure S42:** Sensitivity analysis for mean HbA1c (%) change from baseline to week 12 after exclusion of a small‐sample study.
**Figure S43:** Sensitivity analysis for any TEAE, the findings after excluding small‐sample studies.
**Figure S44:** Sensitivity analysis for any TEAE after excluding phase I and II studies.
**Figure S45:** Sensitivity analysis for thyroid cancer after exclusion of phase I and II studies.
**Figure S46:** Sensitivity analysis for headache outcomes after exclusion of phase I and II studies.

## Data Availability

Data are available from the corresponding author upon reasonable request.
