# Supplementary material for: Metabolic and Glycemic Effects of Orforglipron, a GLP‐1 Receptor Agonist, in Adults With or Without Diabetes: A Network Meta‐Analysis of Randomised Clinical Trials
Source: Endocrinol Diabetes Metab. 2026 Jul 22;9(4):e70275. doi: 10.1002/edm2.70275 (PMC13392217; doi:10.1002/edm2.70275)

Supplementary Appendix

**Metabolic and Glycemic Effects of Orforglipron, a GLP-1 Receptor Agonist, in Adults with or without Diabetes: A Network Meta-analysis of Randomized Clinical Trials.**

**Authors:**

Ahmed W. Hageen, MBBCh^1^; Ahmed Farid Gadelmawla, MD*^2,3^; Ahmad Omar Saleh, MD*^4^; Mohamed Reyad Mohamed, MD^5,6^; Abdallfatah Abdallfatah, MBBCh^7^; Ahmed Elsekhary, MBBCh^8^; Amira Fahmy El-Nemr, MBBCh^9^, Safir Eladawi, MBBCh^10^; Odai Maihoub, MD^11^; Hind Abdulhay, MD^12^; Mustafa Turkmani, MD^13,14^; Basel Abdelazeem, MD^15^; Gregg C. Fonarow, MD, FACC^16,17^.

**Affiliations:**

1. Faculty of Medicine, Tanta University, Tanta, Egypt
2. Faculty of Medicine, Menoufia University, Menoufia, Egypt.
3. Medical Research Group of Egypt (MRGE), Negida Academy, Arlington, MA, USA.
4. Faculty of medicine, The university of Jordan, Amman, Jordan.
5. Department of Medicine, University of Arizona College of Medicine - Phoenix, Phoenix, AZ, USA.
6. Banner Desert Medical Center, Mesa, Arizona, USA.
7. Faculty of Medicine, October 6 University, Giza, Egypt.
8. Kasr Alainy School of Medicine, Cairo University, Cairo, Egypt.
9. Faculty of Medicine, Al-Azhar University, Cairo, Egypt.
10. Faculty of Medicine, Ain Shams University, Cairo, Egypt.
11. Department of Pathology, National Hospital, Latakia, Syria.
12. Faculty of Medicine, Mansoura University, Daqahliyah, Egypt.
13. Faculty of Medicine, Michigan State University, East Lansing, Michigan, USA.
14. Division of Pulmonary and Critical Care, University of Toledo, Toledo, Ohio, USA.
15. Department of Cardiology, West Virginia University, Morgantown, WV, USA.
16. University of California Los Angeles, Los Angeles, California, USA.
17. Ahmanson-UCLA Cardiomyopathy Center, Ronald Reagan UCLA Medical Center, Los Angeles, California.

| **APPENDIX Page** | |
| --- | --- |
| **TABLES** | |
| **Table S1.** Detailed search strategy for each database. | 6 |
| **Table S2.** Preferred Reporting Items for Systematic Reviews and Meta-Analysis (PRISMA) checklist. | 7 |
| **FIGURES** | |
| **Figure S1.** Forest plots of categorical weight-loss thresholds at week 26: **(A)** participants achieving ≥5% weight loss; **(B)** participants achieving ≥10% weight loss; **(C)** participants achieving ≥15% weight loss. Odds ratios (ORs) with 95% confidence intervals (CIs) are shown. | 11 |
| **Figure S2.** Network graphs for categorical weight-loss thresholds at week 26: **(A)** participants achieving ≥5% weight loss; **(B)** participants achieving ≥10% weight loss; **(C)** participants achieving ≥15% weight loss. | 12 |
| **Figure S3.** Network graphs for glycemic outcomes: **(A)** mean change in HbA1c from baseline to week 12; **(B)** change in fasting glucose from baseline at week 12 (mg/dL); **(C)** rescue therapy for severe, persistent hyperglycemia. | 13 |
| **Figure S4.** Forest plot for safety outcomes: **(A)** any TEAE; **(B)** hypoglycaemia with plasma glucose <54 mg/dL; **(C)** headache; **(D)** thyroid cancer. Odds ratios (ORs) with 95% confidence intervals (CIs) are shown. | 14 |
| **Figure S5**. Network graphs for safety outcomes: **(A)** any TEAE; **(B)** hypoglycaemia with plasma glucose <54 mg/dL; **(C)** headache; **(D)** thyroid cancer. | 15 |
| **Figure S6**. Subgroup analysis by diabetes status for total body weight change from baseline at week 12. | 16 |
| **Figure S7**. Subgroup analysis by diabetes status for total body weight change from baseline at week 26. | 17 |
| **Figure S8**. Subgroup analysis by diabetes status for total body weight change from baseline at week 36. | 18 |
| **Figure S9**. Subgroup analysis by diabetes status for change from baseline in body mass index (kg/m²) at week 12. | 18 |
| **Figure S10.** Subgroup analysis by diabetes status for change from baseline in body mass index (kg/m²) at week 26. | 19 |
| **Figure S11**. Subgroup analysis by diabetes status for change from baseline in waist circumference (cm) at week 12. | 20 |
| **Figure S12**. Subgroup analysis by diabetes status for change from baseline in waist circumference (cm) at week 26. | 20 |
| **Figure S13**. Subgroup analysis by diabetes status for change from baseline in waist circumference (cm) at week 36. | 21 |
| **Figure S14**. Subgroup analysis by diabetes status for categorical weight loss at week 26: participants achieving ≥5% weight loss. | 21 |
| **Figure S15**. Subgroup analysis by diabetes status for categorical weight loss at week 26: participants achieving ≥10% weight loss. | 22 |
| **Figure S16**. Subgroup analysis by diabetes status for categorical weight loss at week 26: participants achieving ≥15% weight loss. | 23 |
| **Figure S17**. Subgroup analysis by diabetes status for any treatment-emergent adverse event (TEAE). | 24 |
| **Figure S18**. Subgroup analysis by diabetes status for headache. | 25 |
| **Figure S19**. Subgroup analysis by diabetes status for thyroid cancer. | 25 |
| **Figure S20**. Side-splitting analysis for body weight change from baseline at week 12. | 26 |

| **Figure S21**. Side-splitting analysis for body weight change from baseline at week 26. | 27 |
| --- | --- |
| **Figure S22**. Side-splitting analysis for body weight change from baseline at week 36. | 28 |
| **Figure S23**. Side-splitting analysis for change from baseline in body mass index (kg/m²) at week 12. | 29 |
| **Figure S24**. Side-splitting analysis for change from baseline in body mass index (kg/m²) at week 26. | 30 |
| **Figure S25**. Side-splitting analysis for change from baseline in body mass index (kg/m²) at week 36. | 31 |
| **Figure S26**. Side-splitting analysis for change from baseline in waist circumference (cm) at week 12. | 32 |
| **Figure S27**. Side-splitting analysis for change from baseline in waist circumference (cm) at week 26. | 33 |
| **Figure S28**. Side-splitting analysis for change from baseline in waist circumference (cm) at week 36. | 34 |
| **Figure S29**. Side-splitting analysis for participants achieving ≥5% weight loss. | 35 |
| **Figure S30**. Side-splitting analysis for participants achieving ≥10% weight loss. | 36 |
| **Figure S31**. Side-splitting analysis for participants achieving ≥15% weight loss. | 37 |
| **Figure S32**. Side-splitting analysis for any TEAE. | 38 |
| **Figure S33**. Side-splitting analysis for hypoglycaemia with plasma glucose <54 mg/dL. | 39 |
| **Figure S34**. Side-splitting analysis for headache. | 40 |
| **Figure S35**. Side-splitting analysis for thyroid cancer. | 41 |
| **Figure S36**. Side-splitting analysis for change in fasting glucose (mg/dL) from baseline at week 12. | 42 |
| **Figure S37**. Side-splitting analysis for mean change in HbA1c from baseline to week 12. | 43 |
| **Figure S38**. Side-splitting analysis for rescue therapy for severe, persistent hyperglycemia. | 44 |
| **Figure S39**. Sensitivity analysis for body weight (kg) change from baseline at week 12 after excluding small-sample studies. | 45 |
| **Figure S40**. Sensitivity analysis for body weight (kg) change from baseline at week 12 after excluding phase I and II studies. | 45 |
| **Figure S41**. Sensitivity analysis for BMI change from baseline at week 12 after excluding phase I and II studies. | 46 |
| **Figure S42**. Sensitivity analysis for mean HbA1c (%) change from baseline to week 12 after exclusion of a small-sample study. | 46 |
| **Figure S43**. Sensitivity analysis for any TEAE, the findings after excluding small-sample studies. | 46 |
| **Figure S44**. Sensitivity analysis for any TEAE after excluding phase I and II studies. | 47 |
| **Figure S45**. Sensitivity analysis for thyroid cancer after exclusion of phase I and II studies. | 47 |
| **Figure S46**. Sensitivity analysis for headache outcomes after exclusion of phase I and II studies. | 47 |

**Table S1:** Detailed search strategy for each database.

| **Database** | **Search Terms** | **Search Field** | | **Results** |
| --- | --- | --- | --- | --- |
| **PubMed** | ("orforglipron" OR "LY3502970" OR "small-molecule oral GLP-1RAs" OR "small-molecule oral GLP-1 receptor agonists" OR "small GLP-1RAs" OR "oral non-peptide glucagon-like peptide-1 receptor agonist" OR "oral active nonpeptide agonist") AND ("type 2 diabetes" OR "T2DM" OR "diabetes mellitus" OR "diabetes" OR "obesity" OR "obese" OR "weight" OR "overweight") | All Fields | 141 | |
| **WOS** | ("orforglipron" OR "LY3502970" OR "small-molecule oral GLP-1RAs" OR "small-molecule oral GLP-1 receptor agonists" OR "small GLP-1RAs" OR "oral non-peptide glucagon-like peptide-1 receptor agonist" OR "oral active nonpeptide agonist") AND ("type 2 diabetes" OR "T2DM" OR "diabetes mellitus" OR "diabetes" OR "obesity" OR "obese" OR "weight" OR "overweight") | All Fields | 51 | |
| **Scopus** | ("orforglipron" OR "LY3502970" OR "small-molecule oral GLP-1RAs" OR "small-molecule oral GLP-1 receptor agonists" OR "small GLP-1RAs" OR "oral non-peptide glucagon-like peptide-1 receptor agonist" OR "oral active nonpeptide agonist") AND ("type 2 diabetes" OR "T2DM" OR "diabetes mellitus" OR "diabetes" OR "obesity" OR "obese" OR "weight" OR "overweight") | Title, Abstract, Keywords | 88 | |
| **EMBASE** | ("orforglipron" OR "LY3502970" OR "small-molecule oral GLP-1RAs" OR "small-molecule oral GLP-1 receptor agonists" OR "small GLP-1RAs" OR "oral non-peptide glucagon-like peptide-1 receptor agonist" OR "oral active nonpeptide agonist") AND ("type 2 diabetes" OR "T2DM" OR "diabetes mellitus" OR "diabetes" OR "obesity" OR "obese" OR "weight" OR "overweight") | All Fields | 131 | |

***Note: one study was retrieved by manual search.***

**Table S2.** Preferred Reporting Items for Systematic Reviews and Meta-Analysis (PRISMA) checklist.

| **Section and Topic** | **Item #** | **Checklist item** | **Page where item is reported** |
| --- | --- | --- | --- |
| **TITLE** | | |  |
| Title | 1 | Identify the report as a systematic review. | 1 |
| **ABSTRACT** | | |  |
| Abstract | 2 | See the PRISMA 2020 for Abstracts checklist. | 4 & 6 |
| **INTRODUCTION** | | |  |
| Rationale | 3 | Describe the rationale for the review in the context of existing knowledge. | 6 |
| Objectives | 4 | Provide an explicit statement of the objective(s) or question(s) the review addresses. | 7-8 |
| **METHODS** | | |  |
| Eligibility criteria | 5 | Specify the inclusion and exclusion criteria for the review and how studies were grouped for the syntheses. | 8 |
| Information sources | 6 | Specify all databases, registers, websites, organisations, reference lists and other sources searched or consulted to identify studies. Specify the date when each source was last searched or consulted. | 7 |
| Search strategy | 7 | Present the full search strategies for all databases, registers and websites, including any filters and limits used. | 7 |
| Selection process | 8 | Specify the methods used to decide whether a study met the inclusion criteria of the review, including how many reviewers screened each record and each report retrieved, whether they worked independently, and if applicable, details of automation tools used in the process. | 7&8 |
| Data collection process | 9 | Specify the methods used to collect data from reports, including how many reviewers collected data from each report, whether they worked independently, any processes for obtaining or confirming data from study investigators, and if applicable, details of automation tools used in the process. | 8&9 |
| Data items | 10a | List and define all outcomes for which data were sought. Specify whether all results that were  compatible with each outcome domain in each study were sought (e.g. for all measures, time points, analyses), and if not, the methods used to decide which results to collect. | 8&9 |

| **Section and Topic** | **Item #** | **Checklist item** | **Page where item is**  **reported** |
| --- | --- | --- | --- |
|  | 10b | List and define all other variables for which data were sought (e.g. participant and intervention characteristics, funding sources). Describe any assumptions made about any missing or unclear information. | 8&9 |
| Study risk of bias assessment | 11 | Specify the methods used to assess risk of bias in the included studies, including details of the tool(s) used, how many reviewers assessed each study and whether they worked independently, and if applicable, details of automation tools used in the process. | 9 |
| Effect  measures | 12 | Specify for each outcome the effect measure(s) (e.g. risk ratio, mean difference) used in the synthesis or presentation of results. | 9&10 |
| Synthesis methods | 13a | Describe the processes used to decide which studies were eligible for each synthesis (e.g. tabulating the study intervention characteristics and comparing against the planned groups for each synthesis (item #5)). | 8 |
|  | 13b | Describe any methods required to prepare the data for presentation or synthesis, such as handling of missing summary statistics, or data conversions. | 9&10 |
|  | 13c | Describe any methods used to tabulate or visually display results of individual studies and syntheses. | 9&10 |
|  | 13d | Describe any methods used to synthesize results and provide a rationale for the choice(s). If  meta-analysis was performed, describe the model(s), method(s) to identify the presence and extent of statistical heterogeneity, and software package(s) used. | 9&10 |
|  | 13e | Describe any methods used to explore possible causes of heterogeneity among study results (e.g. subgroup analysis, meta-regression). | 9&11 |
|  | 13f | Describe any sensitivity analyses conducted to assess robustness of the synthesized results. | 11 |
| Reporting bias assessment | 14 | Describe any methods used to assess risk of bias due to missing results in a synthesis (arising from reporting biases). | 9 &11 |
| Certainty assessment | 15 | Describe any methods used to assess certainty (or confidence) in the body of evidence for an outcome. | NA |
| **RESULTS** | | |  |

| **Section and Topic** | **Item #** | **Checklist item** | **Page where item is**  **reported** |
| --- | --- | --- | --- |
| Study selection | 16a | Describe the results of the search and selection process, from the number of records identified in the search to the number of studies included in the review, ideally using a flow diagram. | 11 |
|  | 16b | Cite studies that might appear to meet the inclusion criteria, but which were excluded, and explain why they were excluded. | 10 &11 |
| Study  characteristics | 17 | Cite each included study and present its characteristics. | 10 &11 |
| Risk of bias in studies | 18 | Present assessments of risk of bias for each included study. | 12 |
| Results of individual studies | 19 | For all outcomes, present, for each study: (a) summary statistics for each group (where appropriate) and (b) an effect estimate and its precision (e.g. confidence/credible interval), ideally using structured tables or plots. | 11 |
| Results of syntheses | 20a | For each synthesis, briefly summarise the characteristics and risk of bias among contributing studies. | 10&11 |
|  | 20b | Present results of all statistical syntheses conducted. If meta-analysis was done, present for each the summary estimate and its precision (e.g. confidence/credible interval) and measures of statistical heterogeneity. If comparing groups, describe the direction of the effect. | 12-21 |
|  | 20c | Present results of all investigations of possible causes of heterogeneity among study results. | 19&20 |
|  | 20d | Present results of all sensitivity analyses conducted to assess the robustness of the synthesized results. | NA |
| Reporting biases | 21 | Present assessments of risk of bias due to missing results (arising from reporting biases) for each synthesis assessed. | 12 |
| Certainty of evidence | 22 | Present assessments of certainty (or confidence) in the body of evidence for each outcome assessed. | NA |
| **DISCUSSION** | | |  |
| Discussion | 23a | Provide a general interpretation of the results in the context of other evidence. | 22 |
|  | 23b | Discuss any limitations of the evidence included in the review. | 26 |

| **Section and Topic** | **Item #** | **Checklist item** | **Page where item is**  **reported** |
| --- | --- | --- | --- |
|  | 23c | Discuss any limitations of the review processes used. | 26 |
|  | 23d | Discuss implications of the results for practice, policy, and future research. | 27 |
| **OTHER INFORMATION** | | |  |
| Registration and protocol | 24a | Provide registration information for the review, including register name and registration number, or state that the review was not registered. | 7 |
|  | 24b | Indicate where the review protocol can be accessed, or state that a protocol was not prepared. | 7 |
|  | 24c | Describe and explain any amendments to information provided at registration or in the protocol. | 7 |
| Support | 25 | Describe sources of financial or non-financial support for the review, and the role of the funders or sponsors in the review. | 29 |
| Competing interests | 26 | Declare any competing interests of review authors. | 29 |
| Availability of data, code and other materials | 27 | Report which of the following are publicly available and where they can be found: template data collection forms; data extracted from included studies; data used for all analyses; analytic code; any other materials used in the review. | 30 |

**Reference:** Page MJ, McKenzie JE, Bossuyt PM, Boutron I, Hoffmann TC, Mulrow CD, et al. The PRISMA 2020 statement: an updated guideline for reporting systematic reviews. BMJ 2021;372:n71. doi: 10.1136/bmj.n71

**Figure S1.** Forest plots of categorical weight-loss (kg) thresholds at week 26: **(A)** participants achieving ≥5% weight loss; **(B)** participants achieving ≥10% weight loss; **(C)** participants achieving ≥15% weight loss. Odds ratios (ORs) with 95% confidence intervals (CIs) are shown.


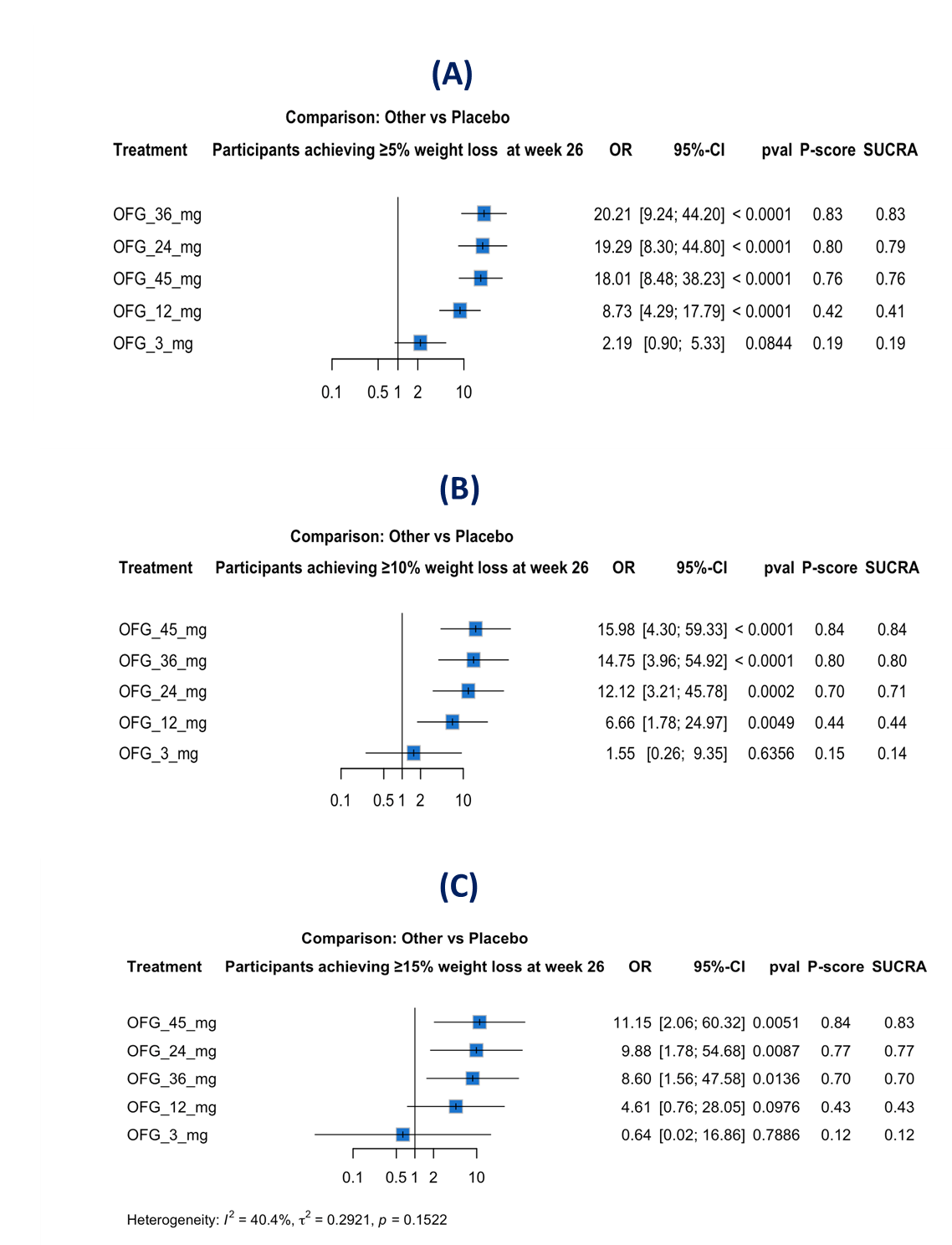


**Figure S2:** Network graphs for categorical weight-loss (kg) thresholds at week 26: **(A)** participants achieving ≥5% weight loss; **(B)** participants achieving ≥10% weight loss; **(C)** participants achieving ≥15% weight loss.


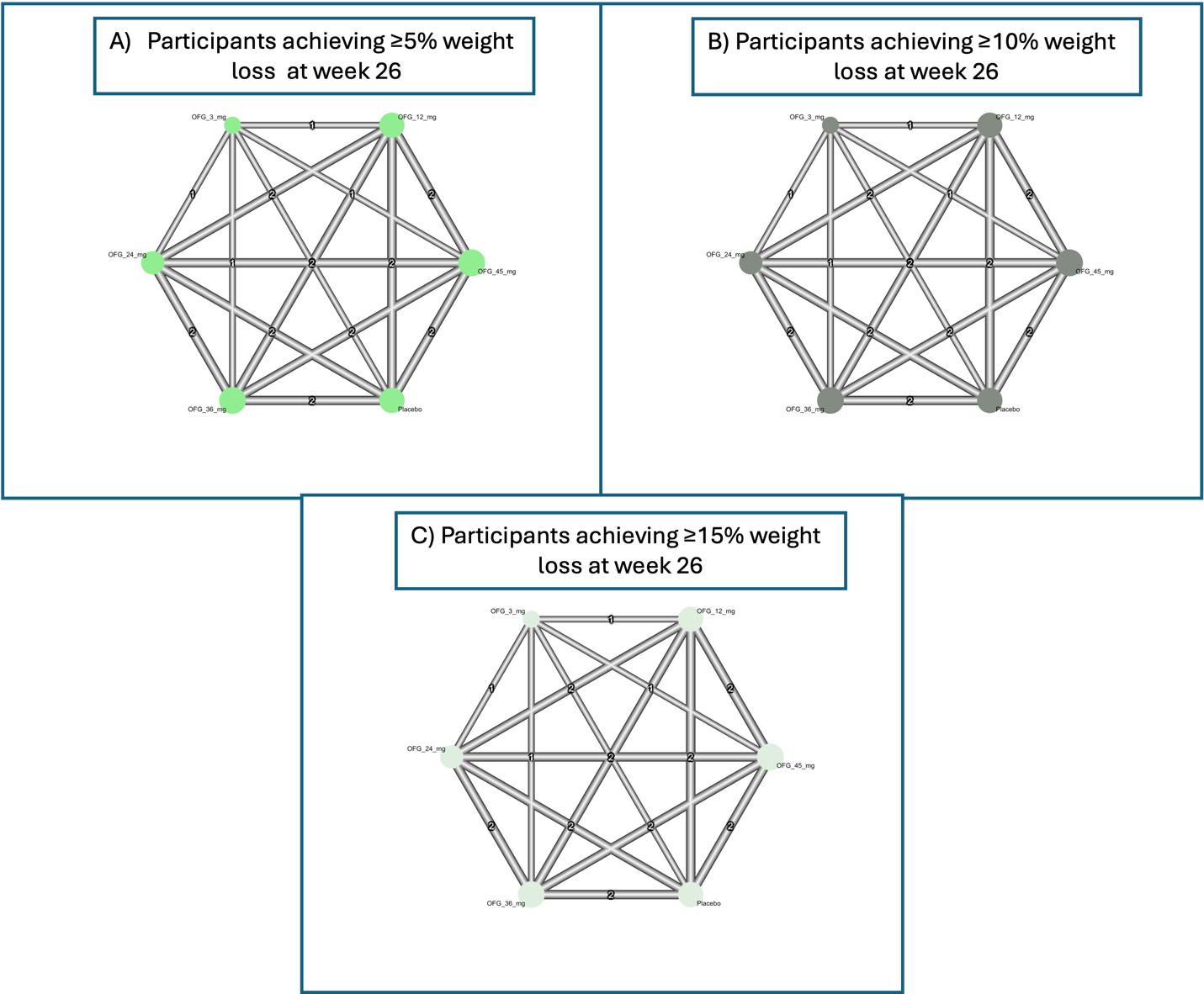


**Figure S3.** Network graphs for glycemic outcomes: **(A)** mean change in HbA1c (%) from baseline to week 12; **(B)** change in fasting glucose from baseline at week 12 (mg/dL); **(C)** rescue therapy for severe, persistent hyperglycemia.


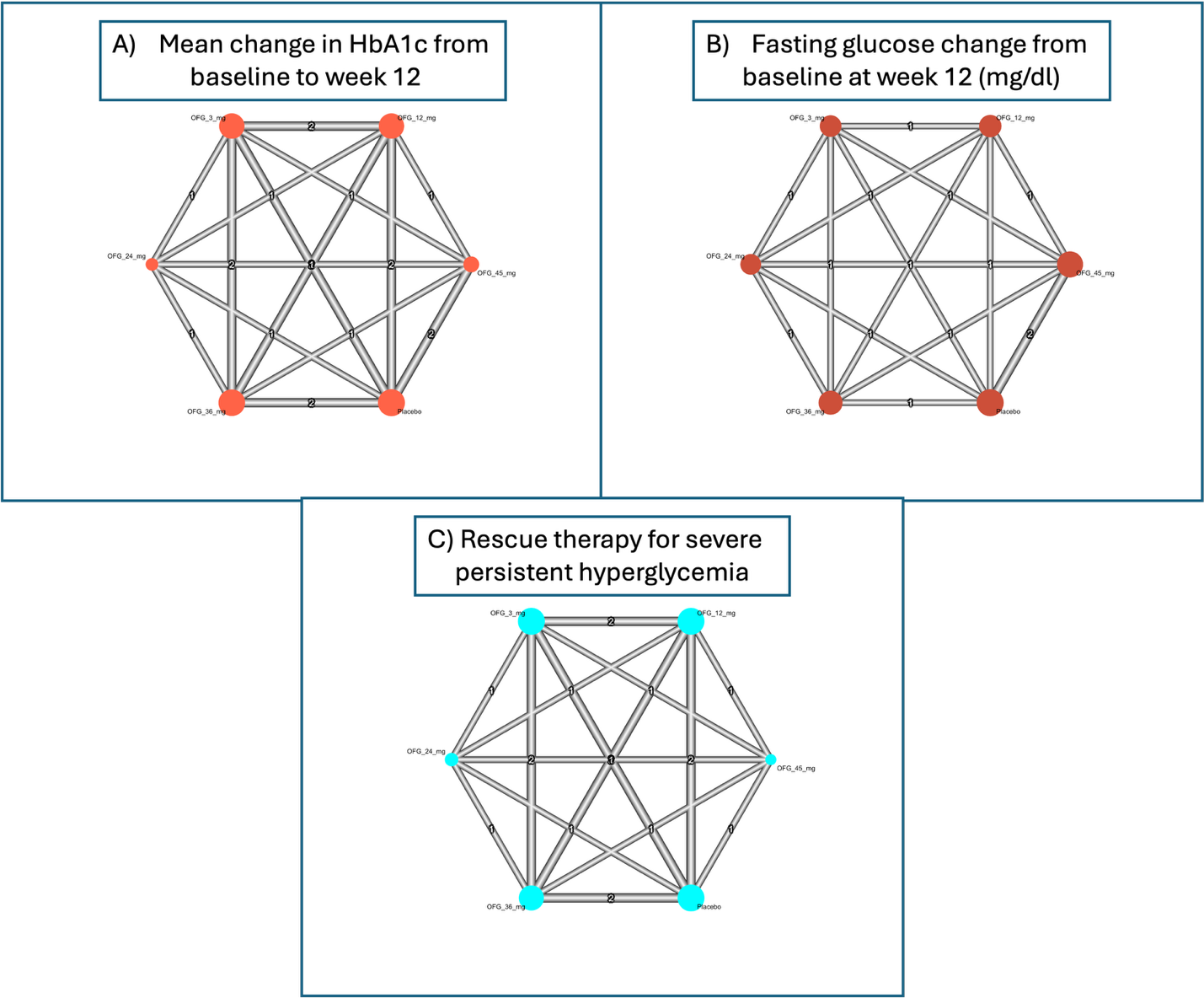


**Figure S4:** Forest plot for safety outcomes: **(A)** any TEAE; **(B)** hypoglycaemia with plasma glucose <54 mg/dL; **(C)** headache; **(D)** thyroid cancer. Odds ratios (ORs) with 95% confidence intervals (CIs) are shown.


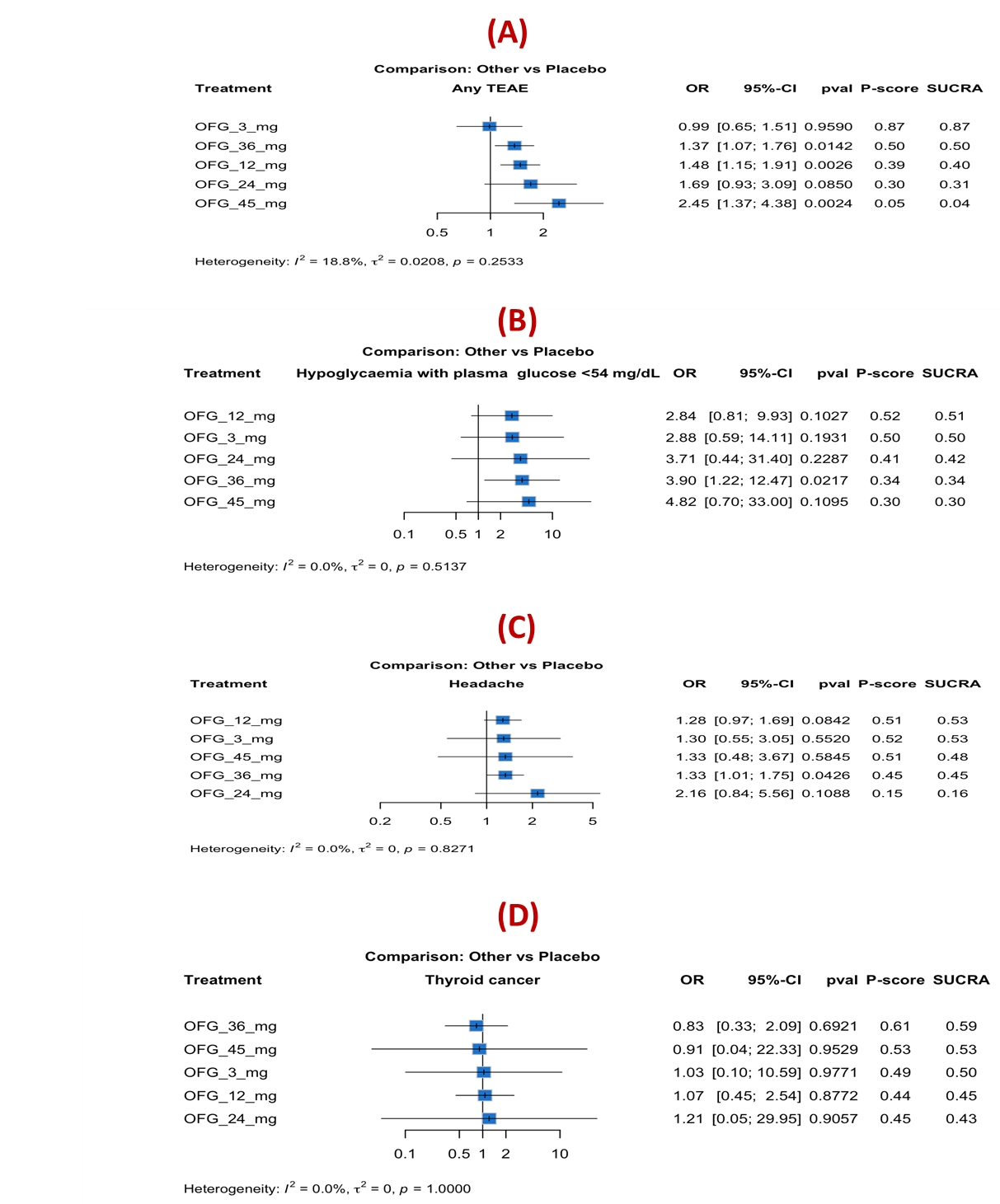


**Figure S5:** Network graphs for safety outcomes: **(A)** any TEAE; **(B)** hypoglycaemia with plasma glucose <54 mg/dL; **(C)** headache; **(D)** thyroid cancer.


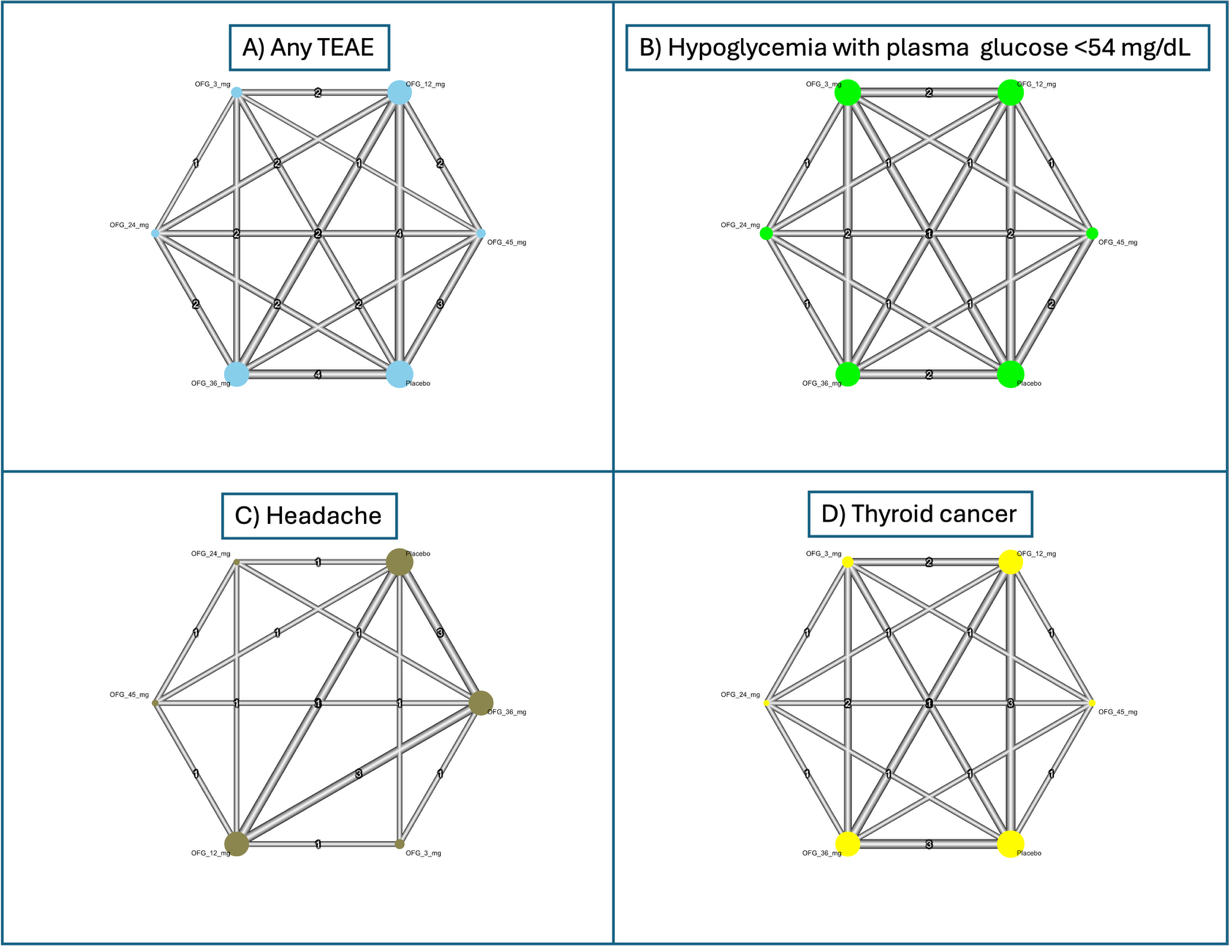


**Figure S6:** Subgroup analysis by diabetes status for total body weight change from baseline at week 12.


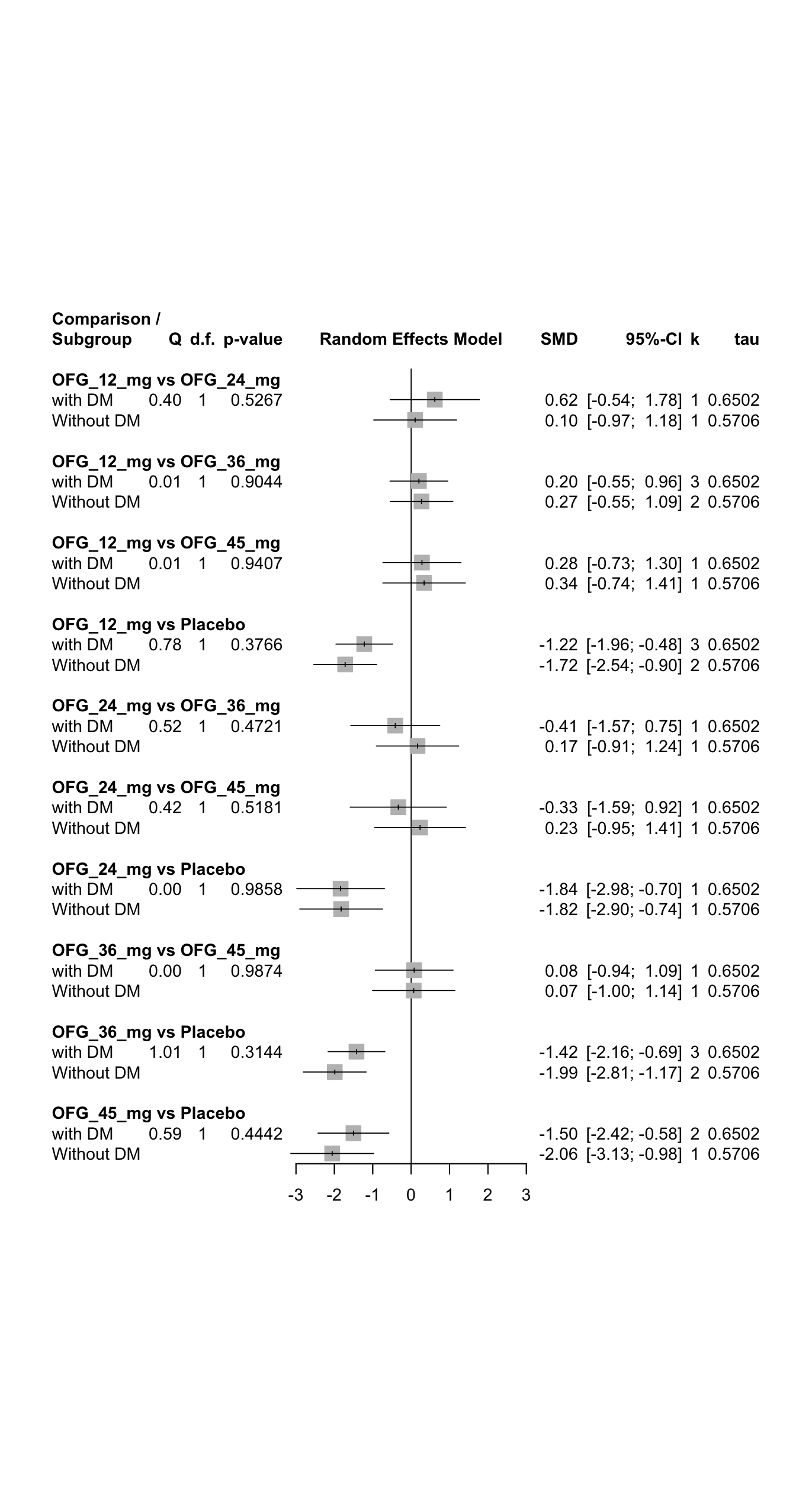


**Figure S7:** Subgroup analysis by diabetes status for total body weight change from baseline at week 26.


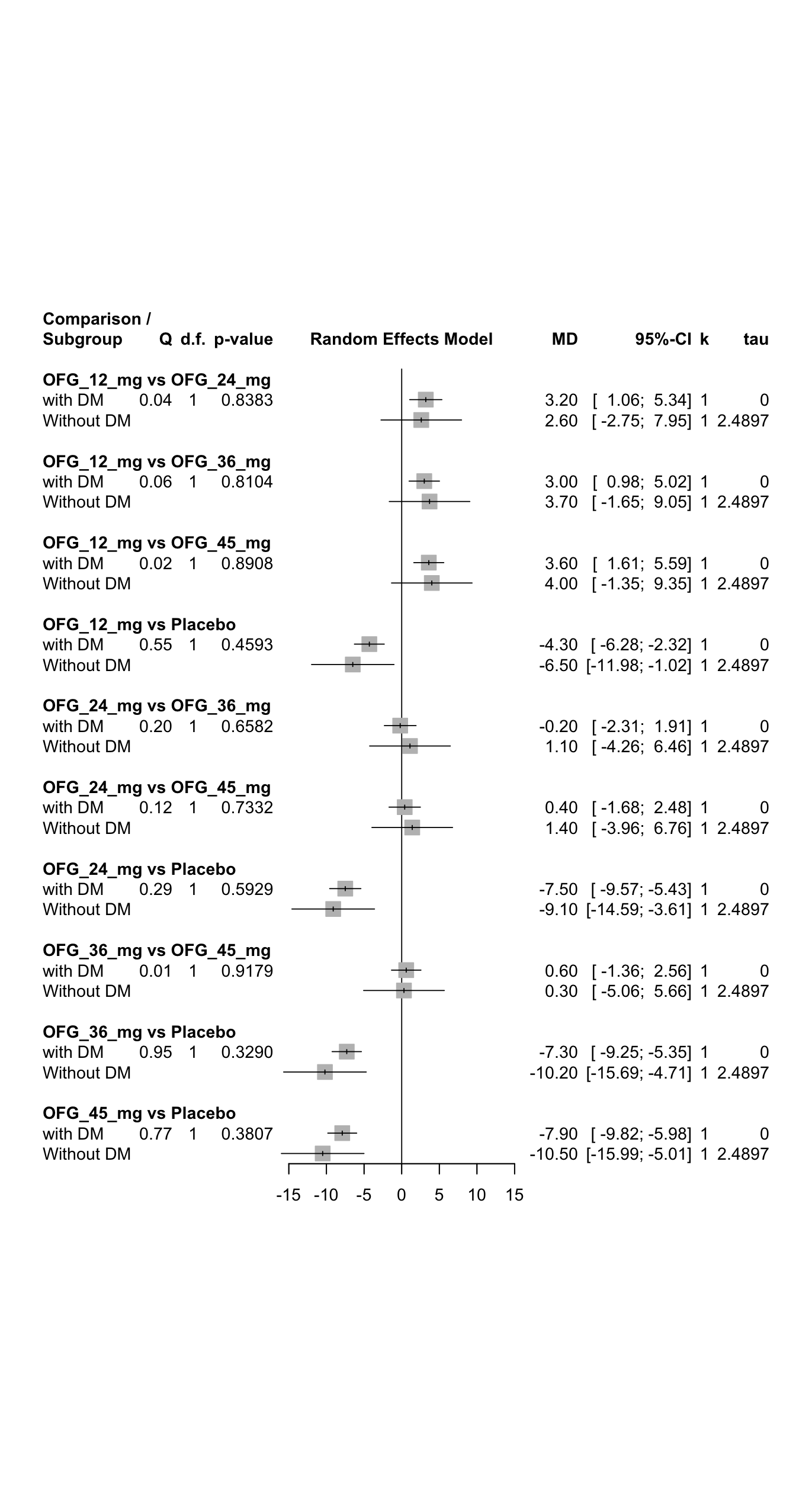


**Figure S8**. Subgroup analysis by diabetes status for total body weight change from baseline at week 36

**
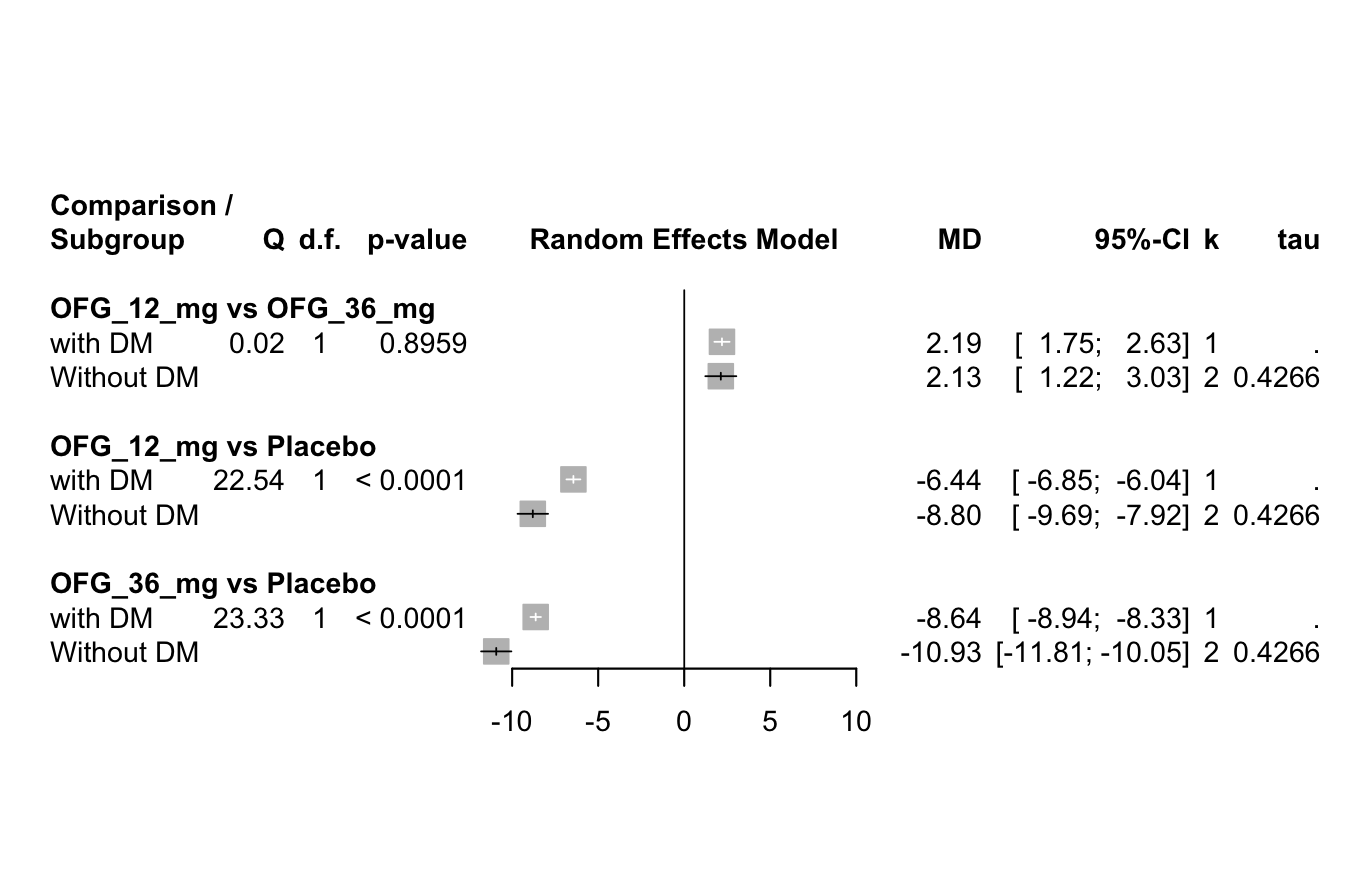
**

**Figure S9:** Subgroup analysis by diabetes status for change from baseline in body mass index (kg/m²) at week 12.


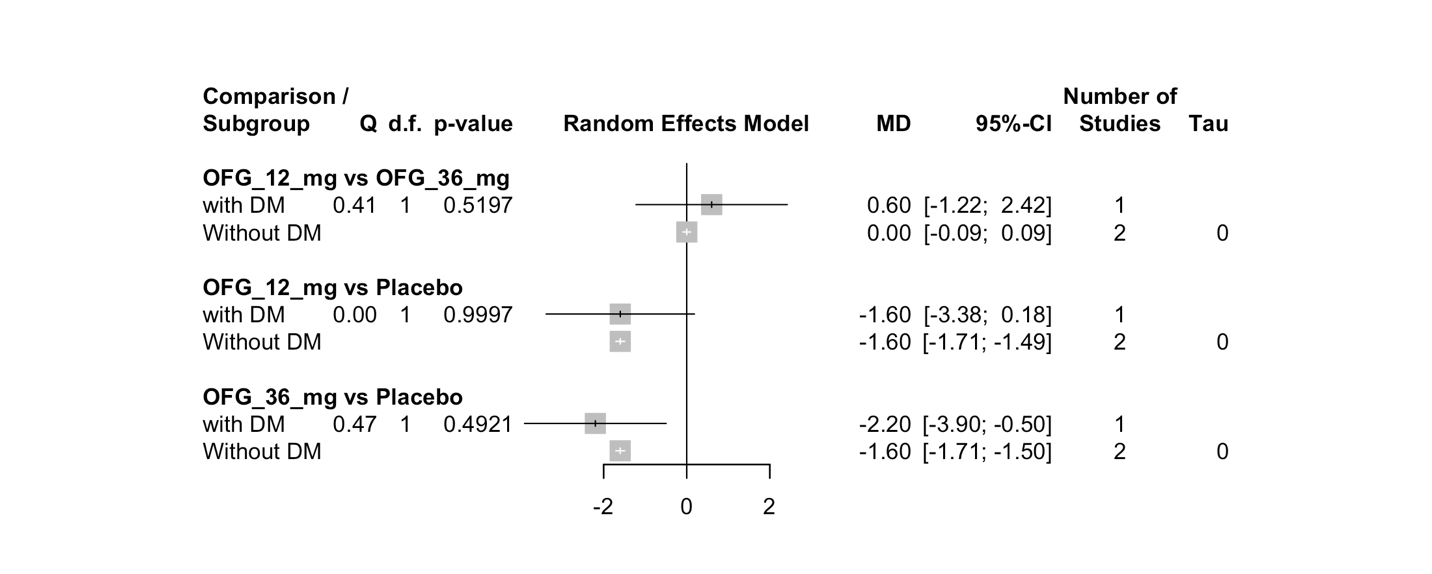


**Figure S10:** Subgroup analysis by diabetes status for change from baseline in body mass index (kg/m²) at week 26.


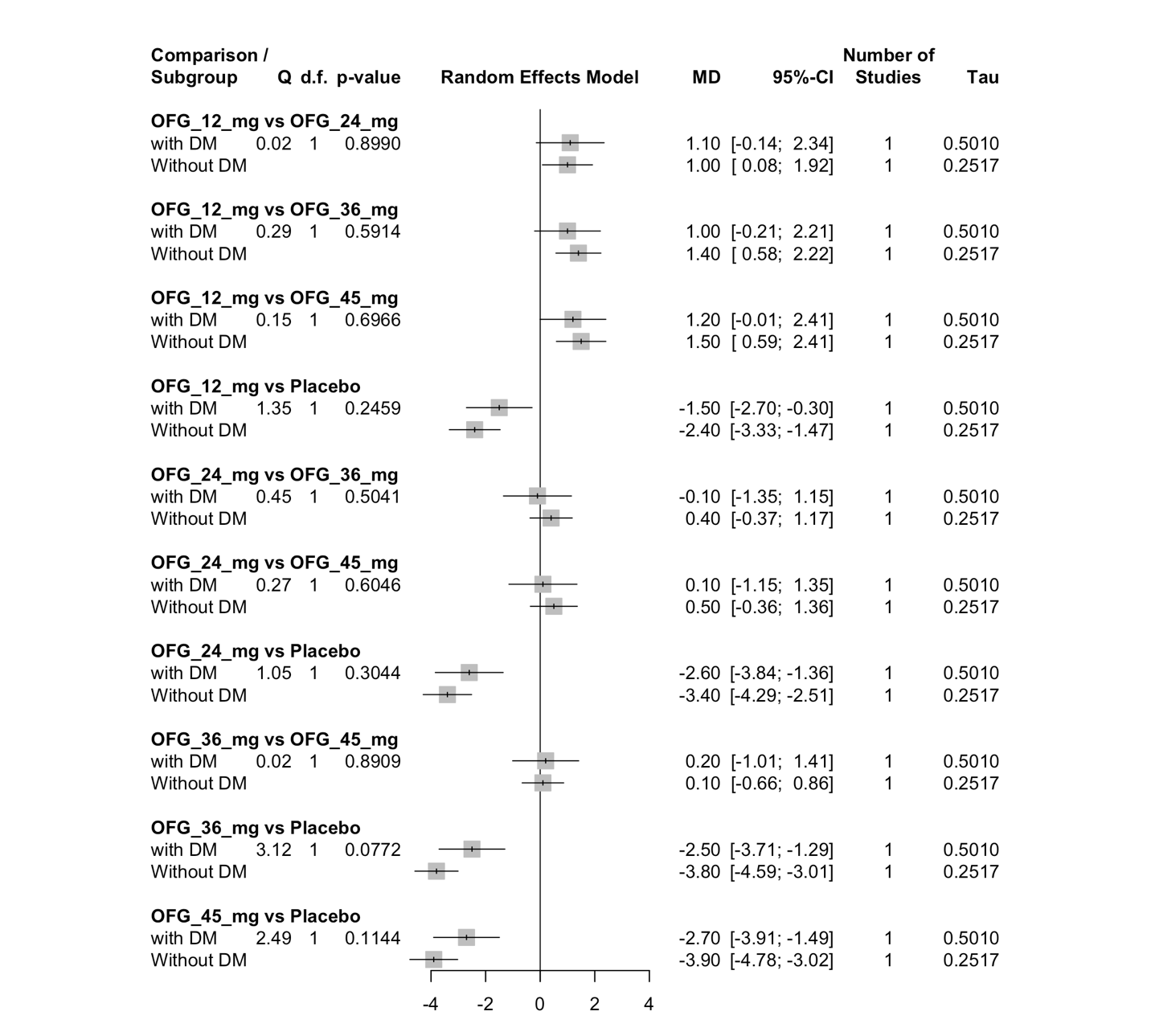


**Figure S11.** Subgroup analysis by diabetes status for change from baseline in waist circumference (cm) at week 12.

**Figure S12:** Subgroup analysis by diabetes status for change from baseline in waist circumference (c.m.) at week 26


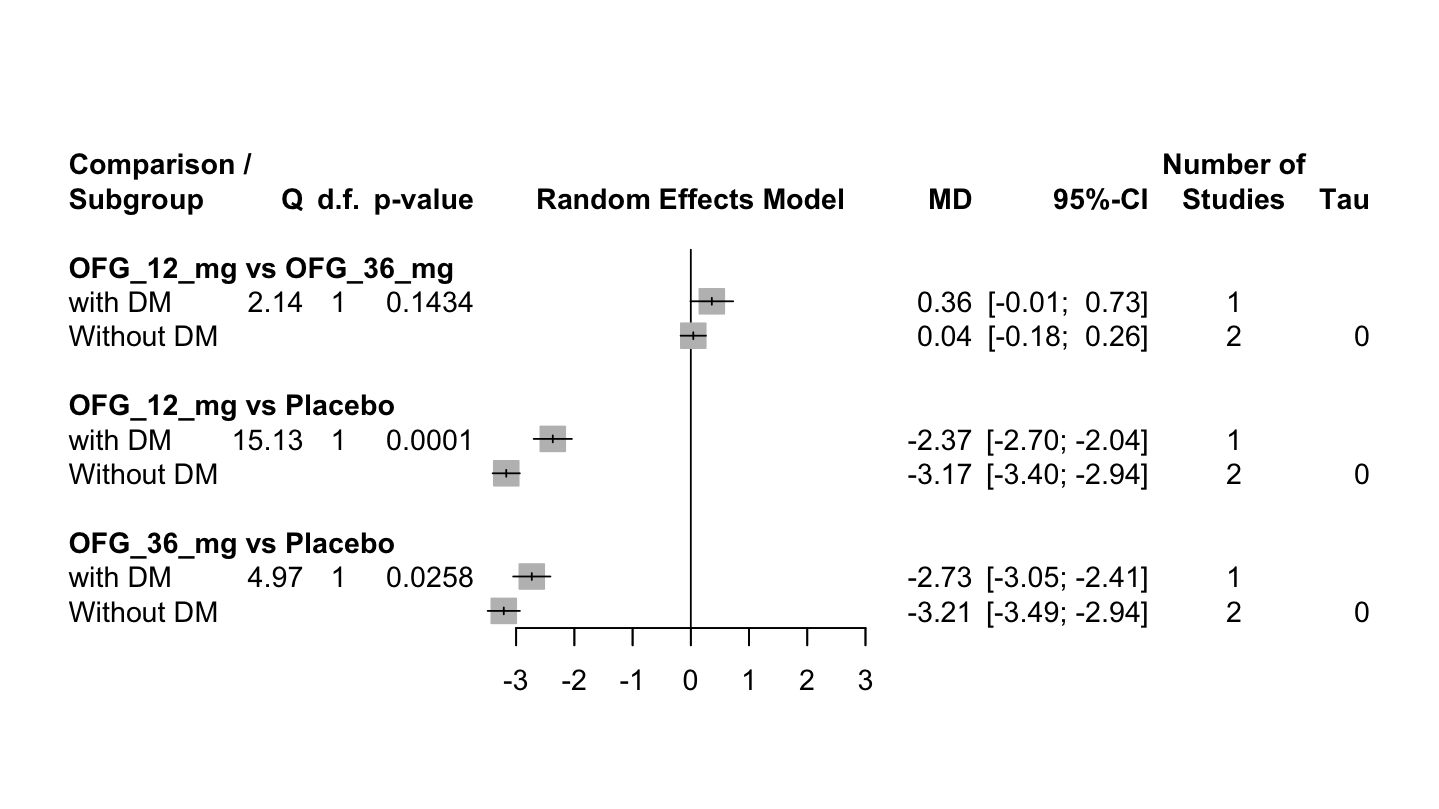


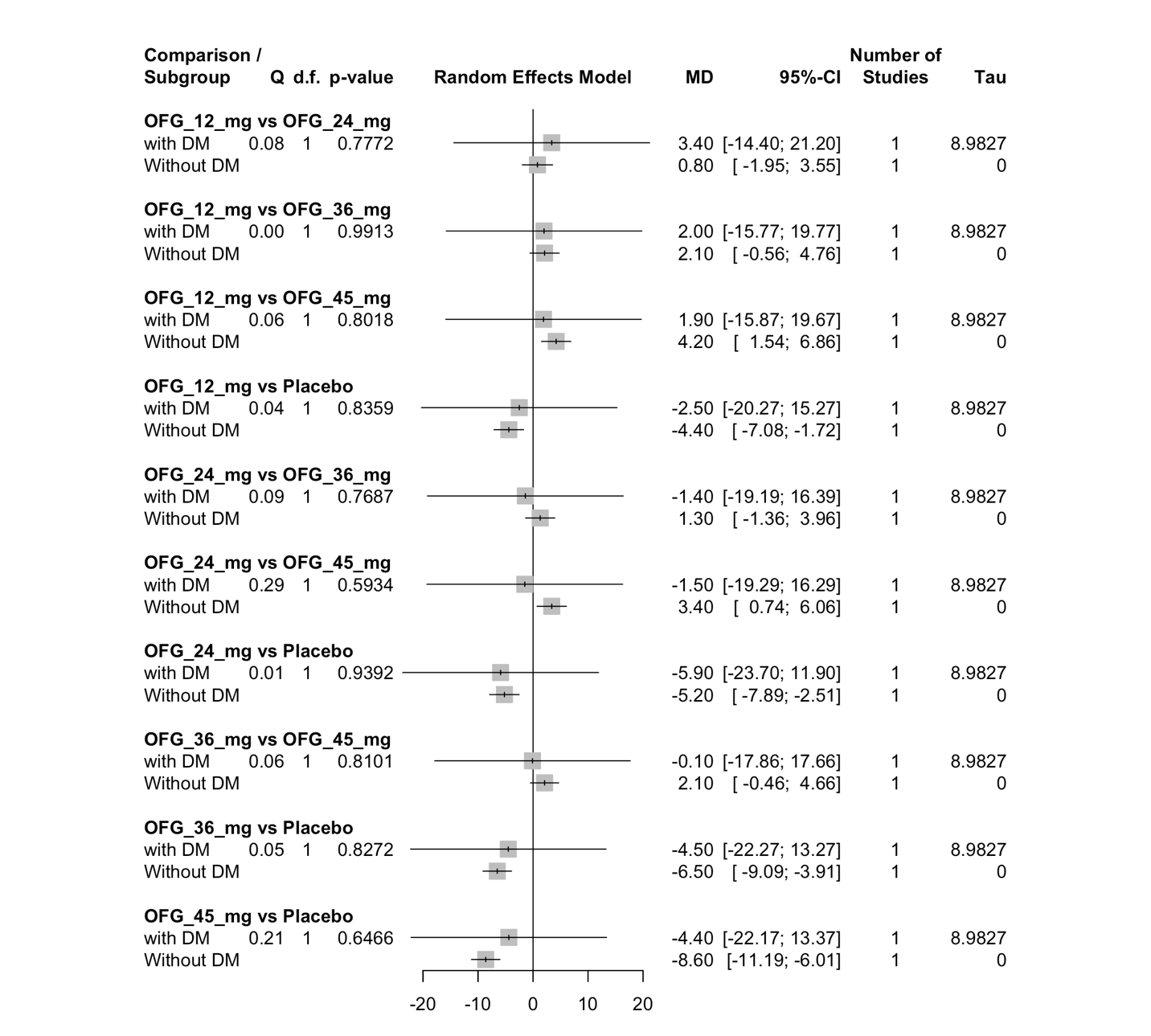


**Figure S13. Subgroup analysis by diabetes status for change from baseline in waist circumference (cm) at week 36.**


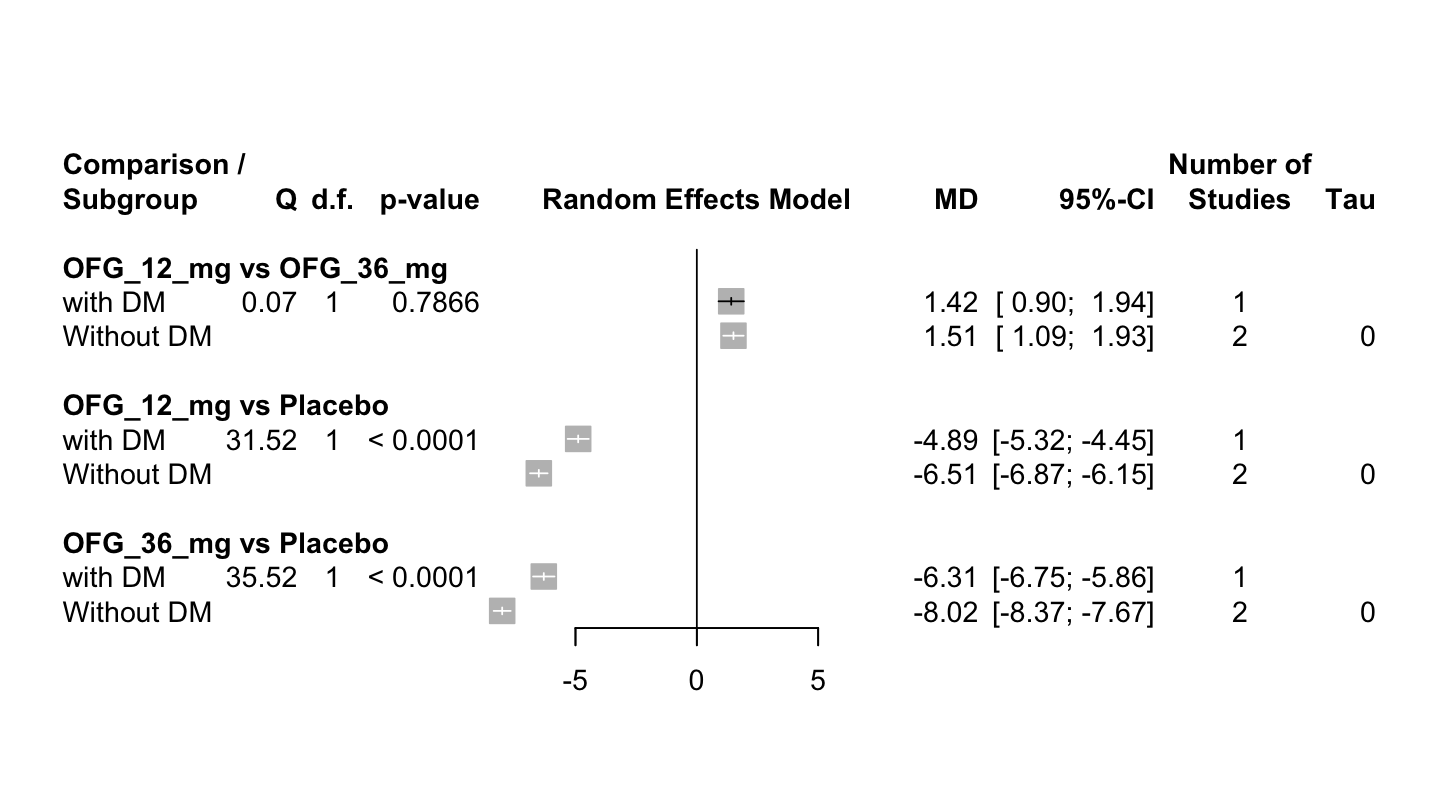


**Figure S14:** Subgroup analysis by diabetes status for categorical weight loss at week 26: participants achieving ≥5% weight loss.


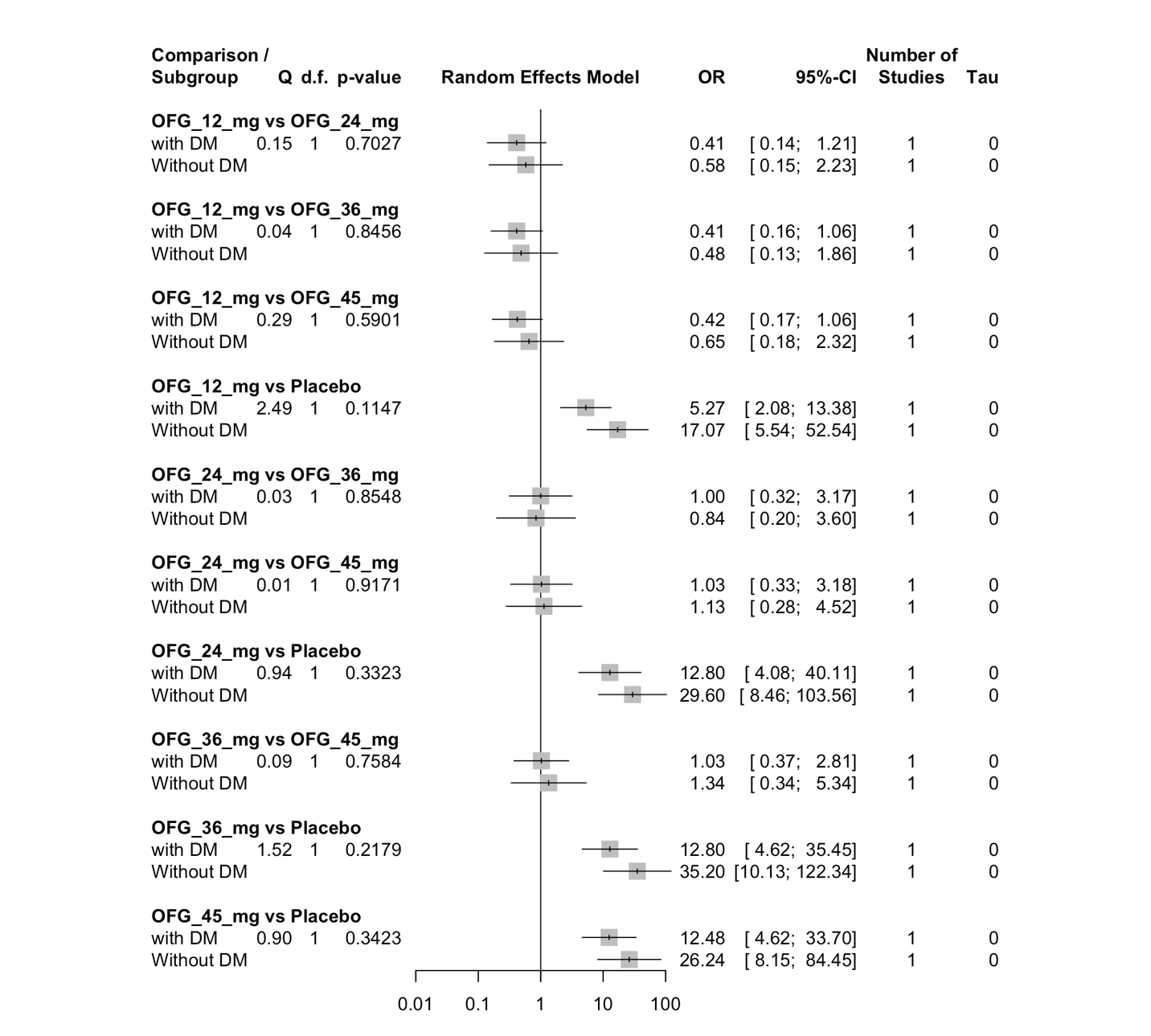


**Figure S15:** Subgroup analysis by diabetes status for categorical weight loss at week 26: participants achieving ≥10% weight loss.


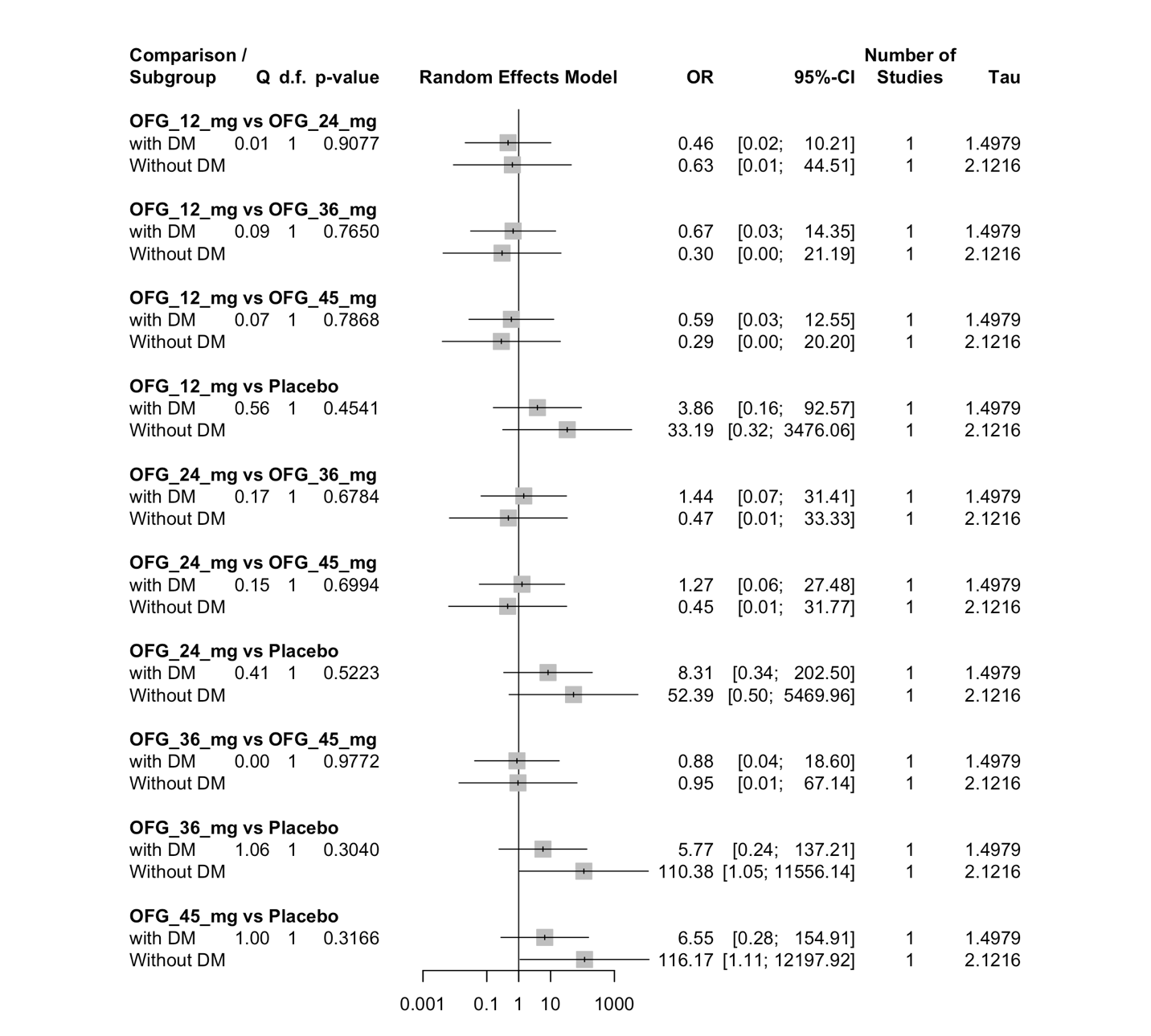


**Figure S16:** Subgroup analysis by diabetes status for categorical weight loss at week 26: participants achieving ≥15% weight loss.


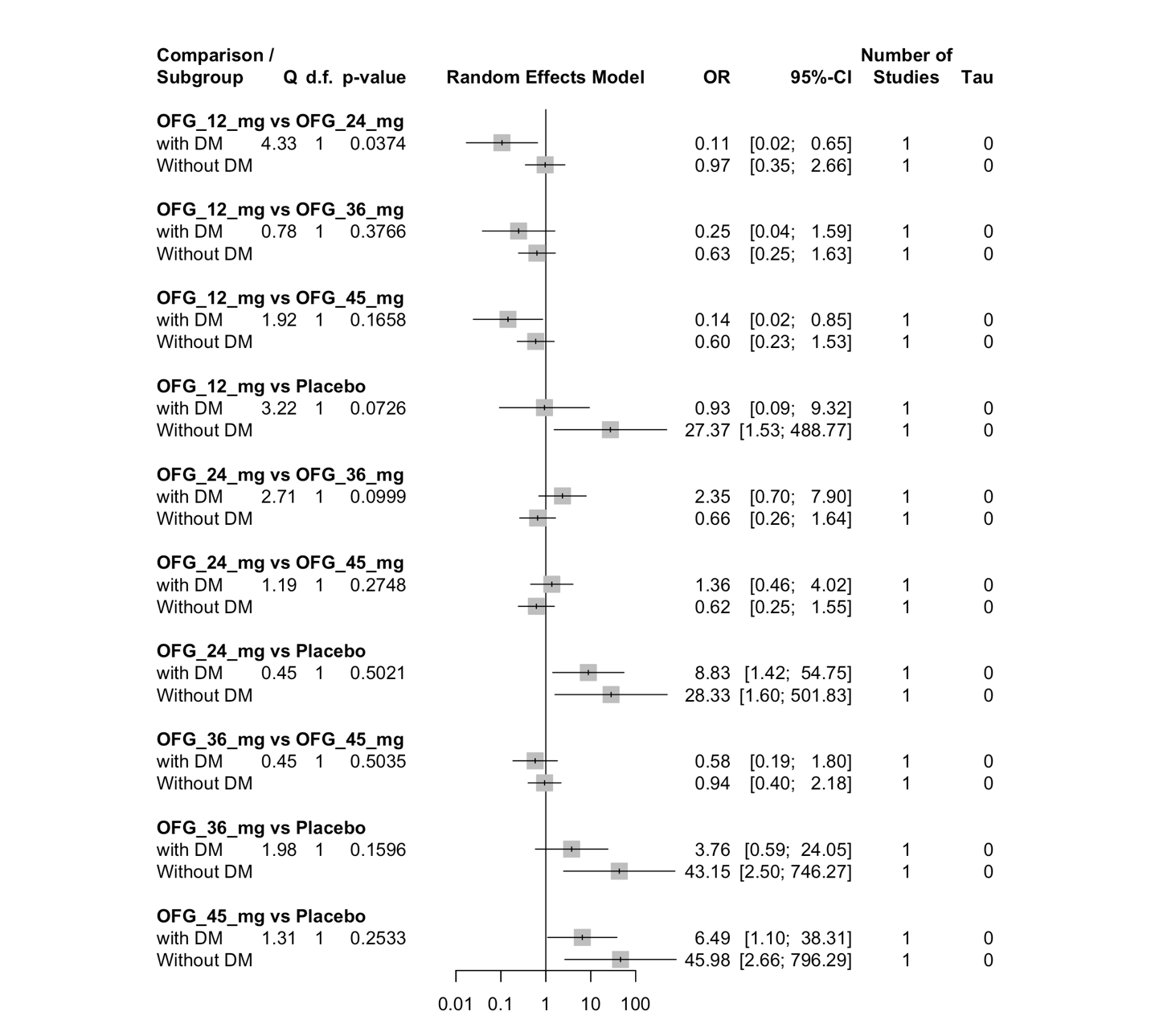


**Figure S17:** Subgroup analysis by diabetes status for any treatment-emergent adverse event (TEAE).


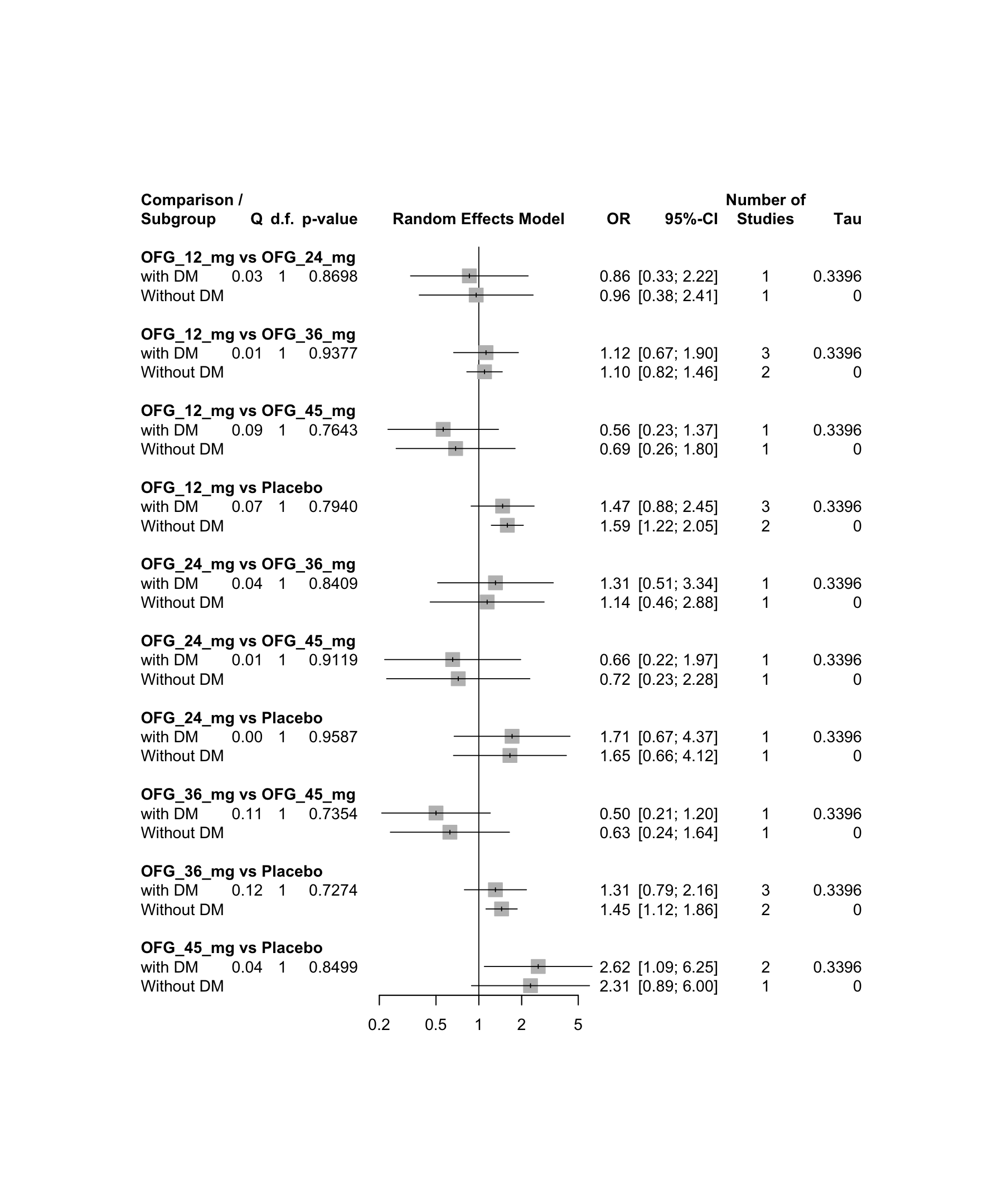


**Figure S18:** Subgroup analysis by diabetes status for headache.


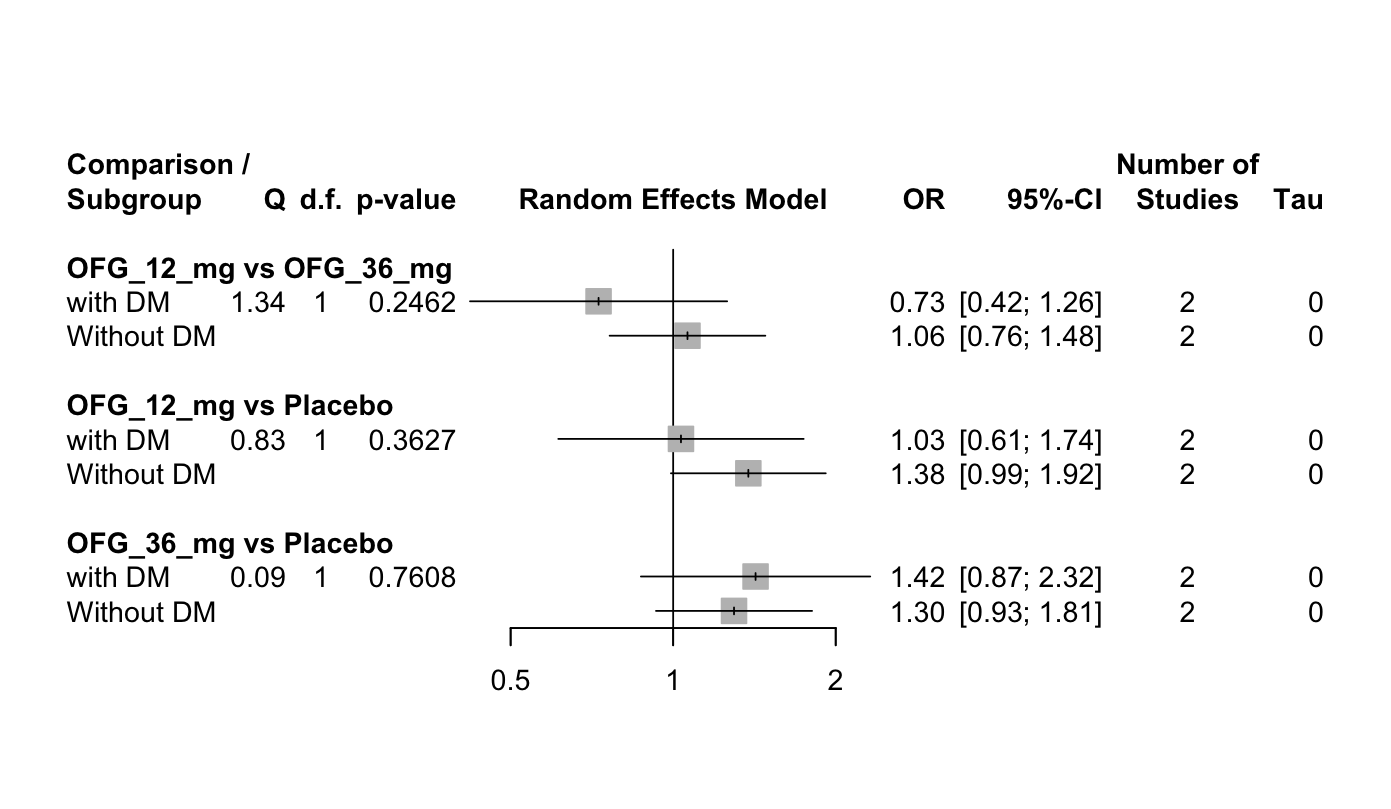


**Figure S19:** Subgroup analysis by diabetes status for thyroid cancer.


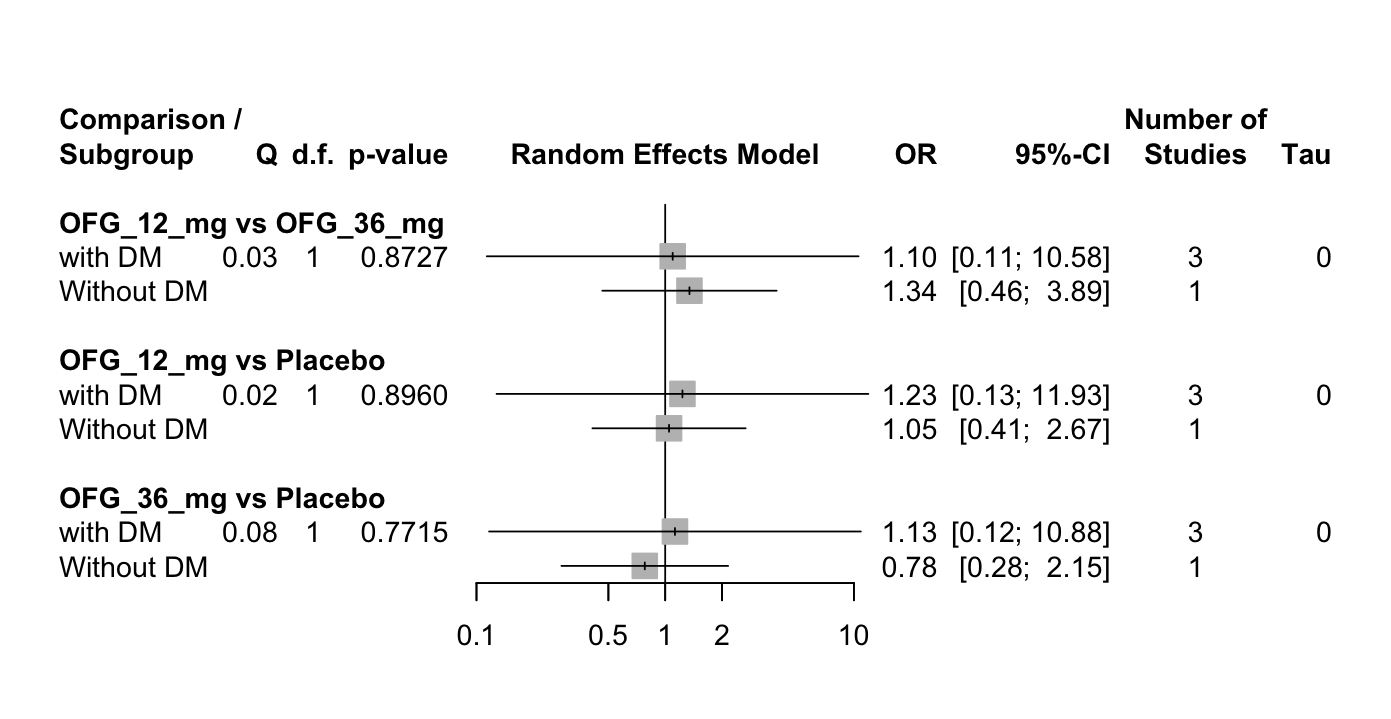


**Figure S20:** Side-splitting analysis for body weight change from baseline at week 12 (Kg).


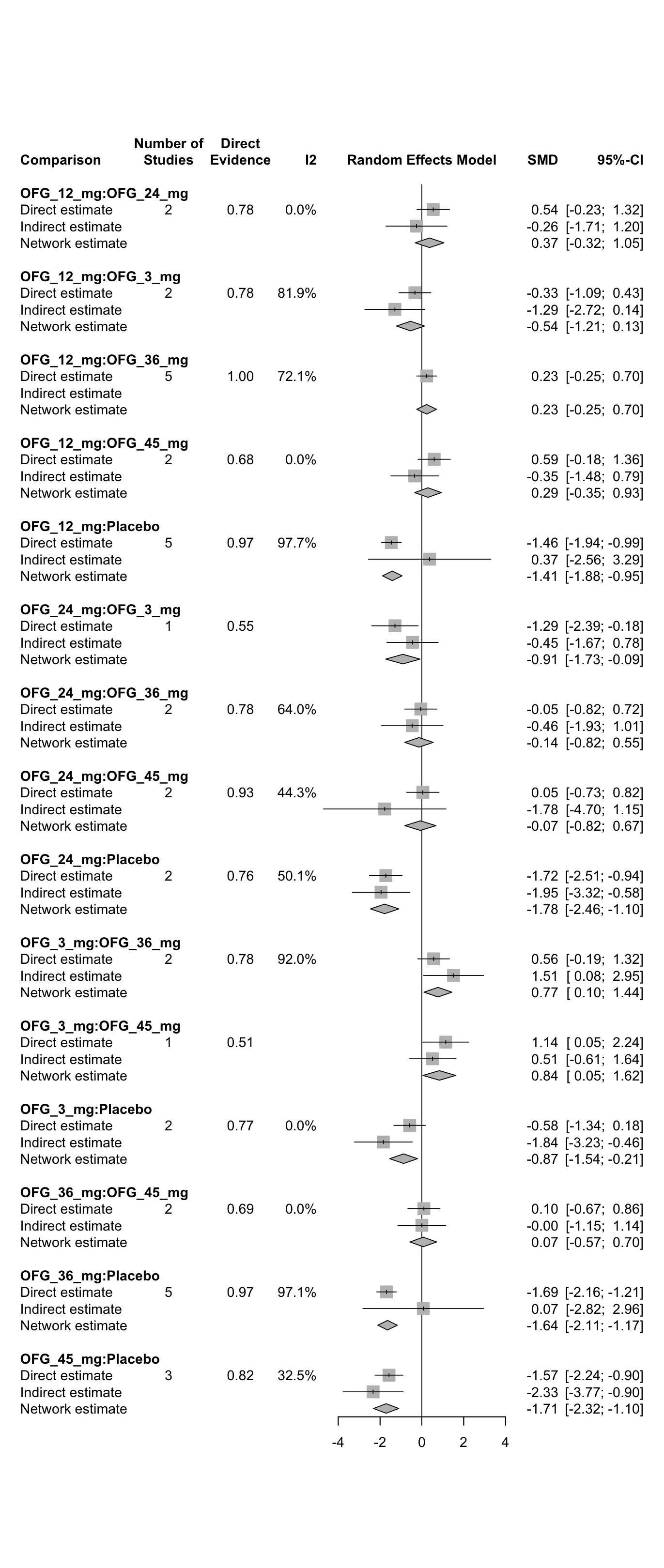


**Figure S21:** Side-splitting analysis for body weight change from baseline at week 26 (Kg).


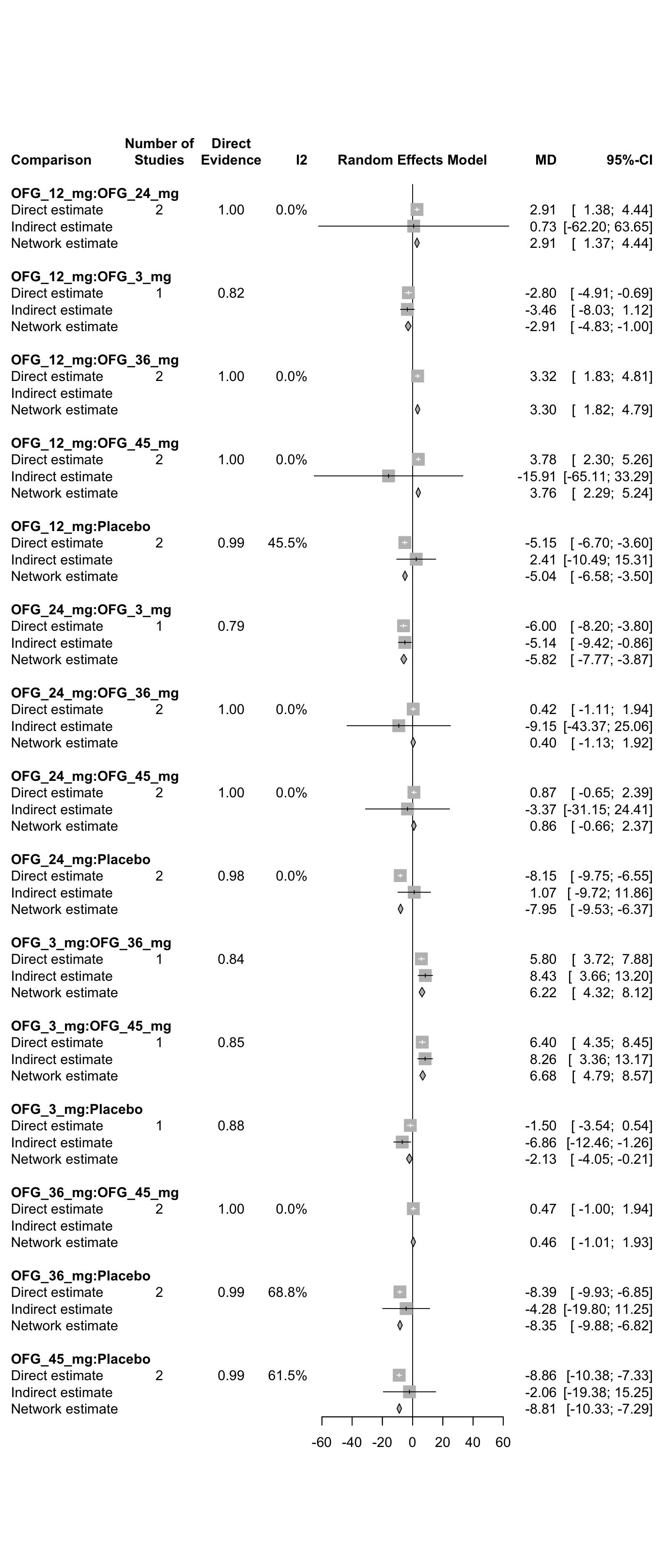


**Figure S22:** Side-splitting analysis for body weight change from baseline at week 36 (Kg).


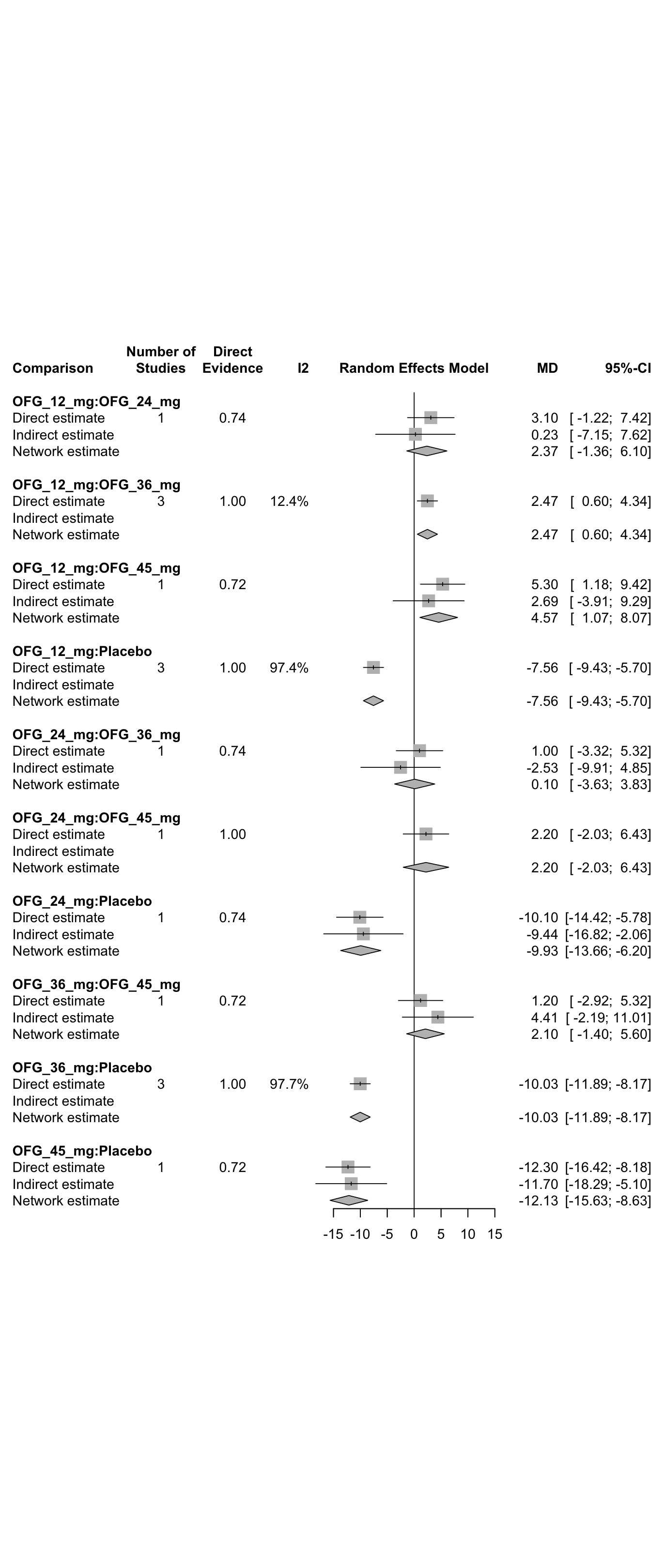


**Figure S23:** Side-splitting analysis for change from baseline in body mass index (kg/m²) at week 12.


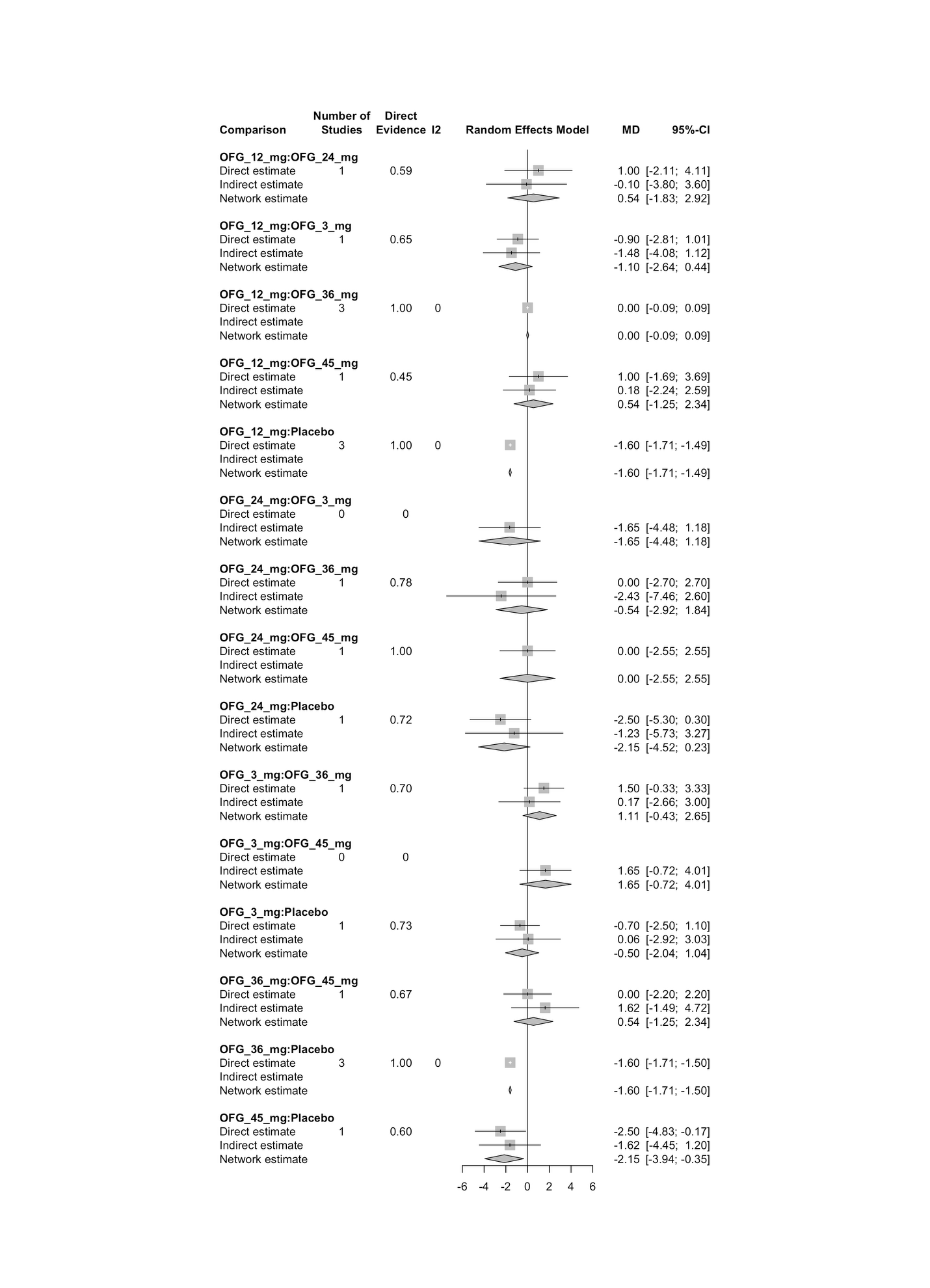


**Figure S24:** Side-splitting analysis for change from baseline in body mass index (kg/m²) at week 26.


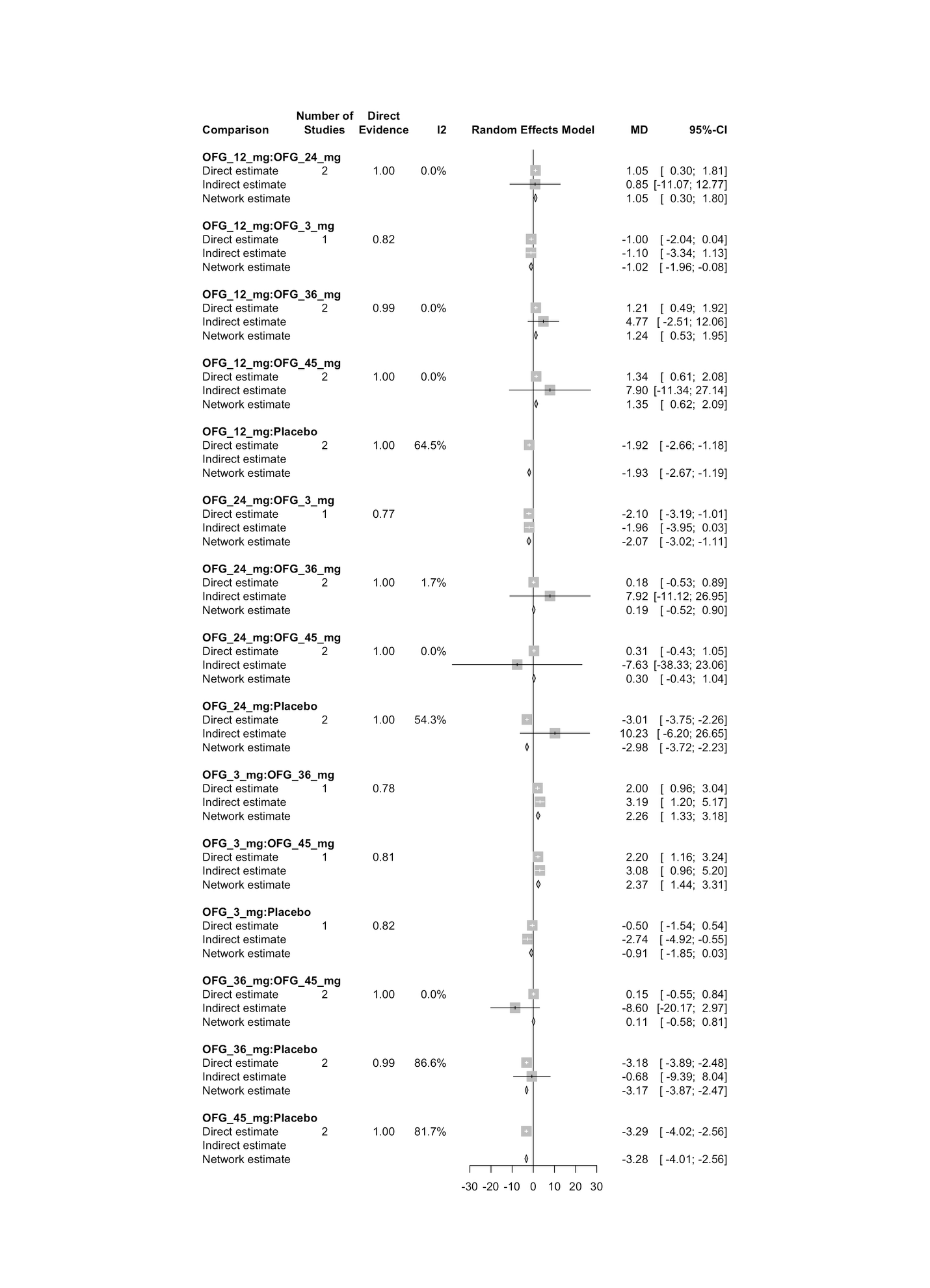


**Figure S25:** Side-splitting analysis for change from baseline in body mass index (kg/m²) at week 36.


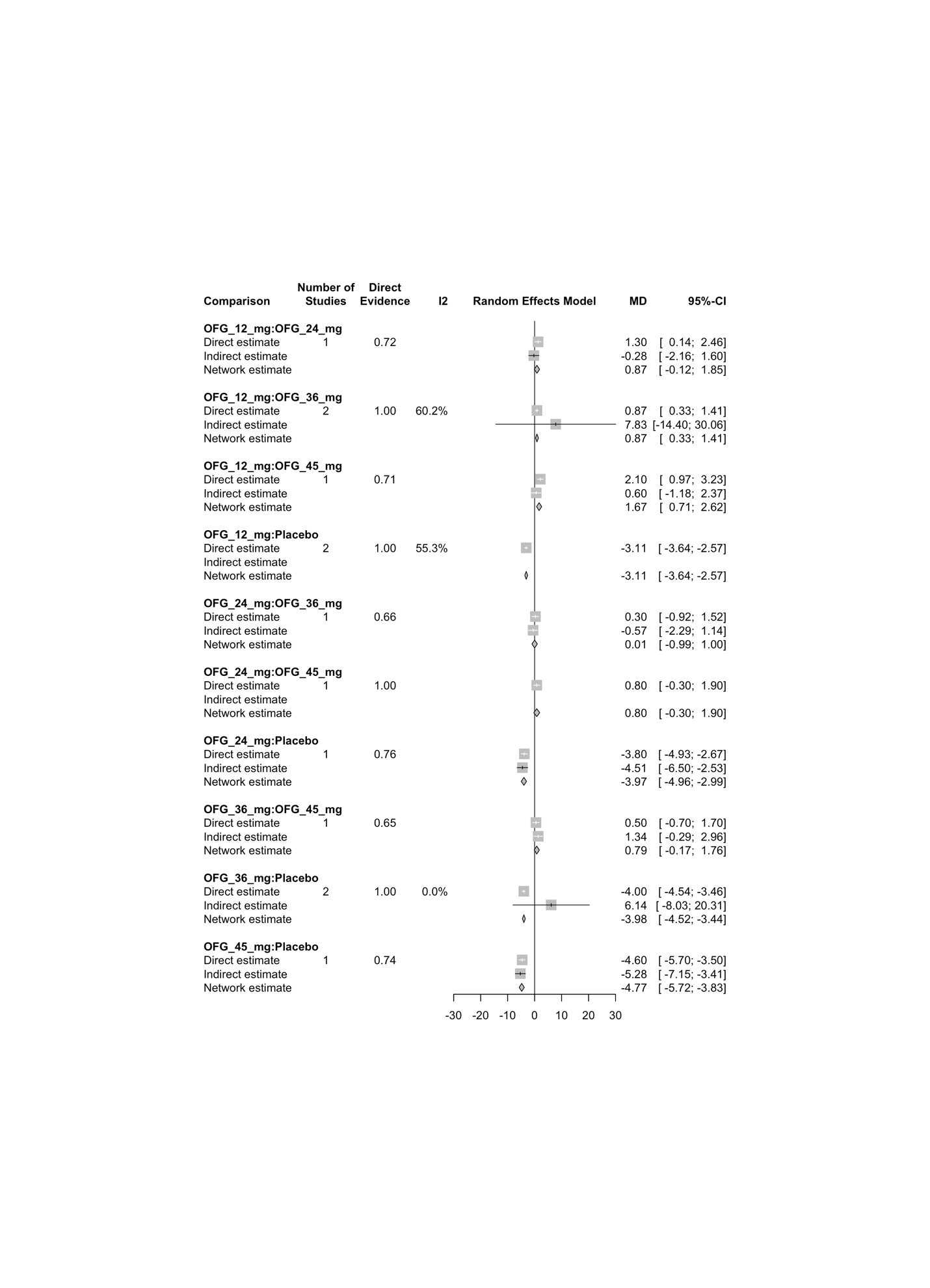


**Figure S26:** Side-splitting analysis for change from baseline in waist circumference (cm) at week 12.


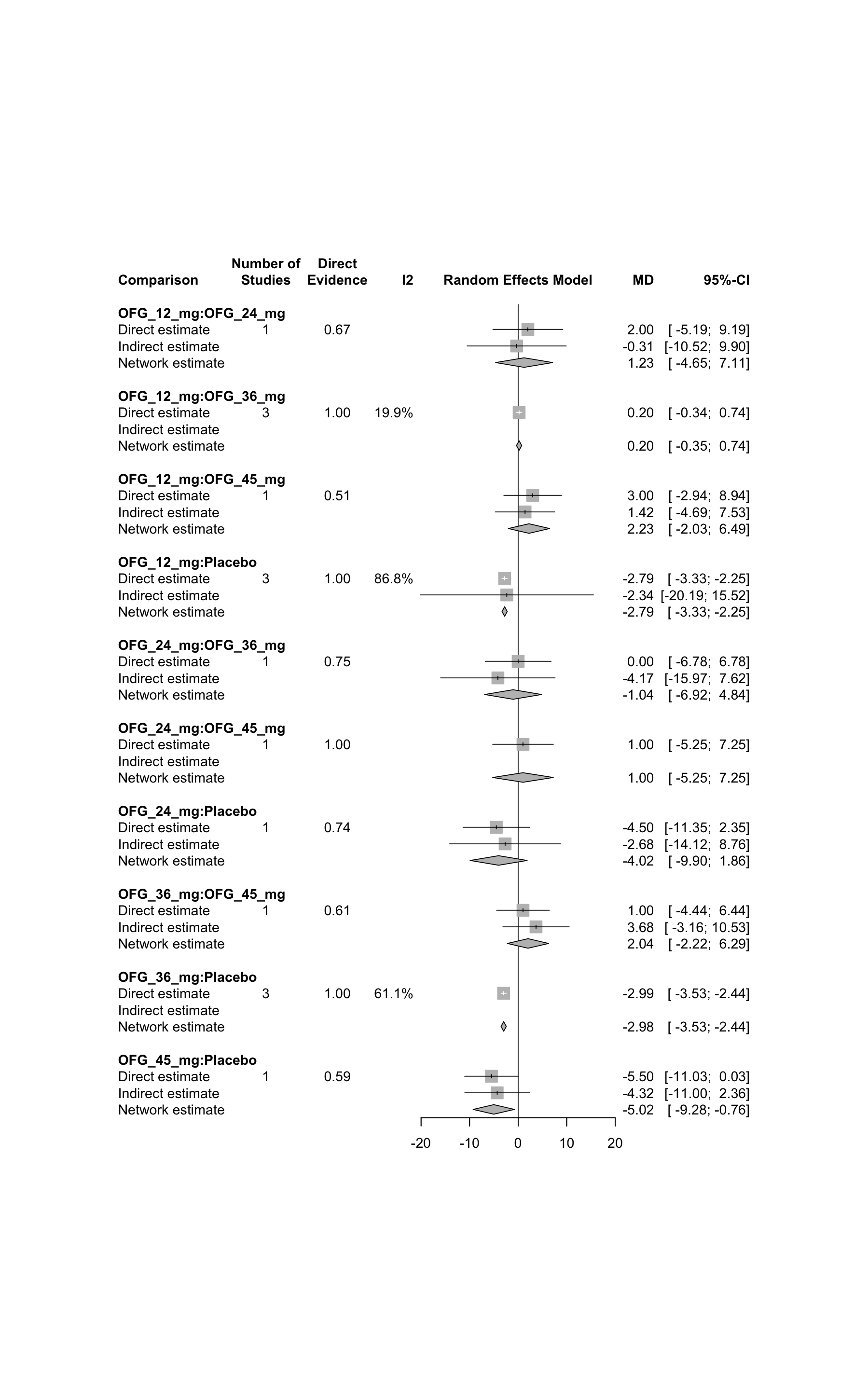


**Figure S27:** Side-splitting analysis for change from baseline in waist circumference (cm) at week 26.


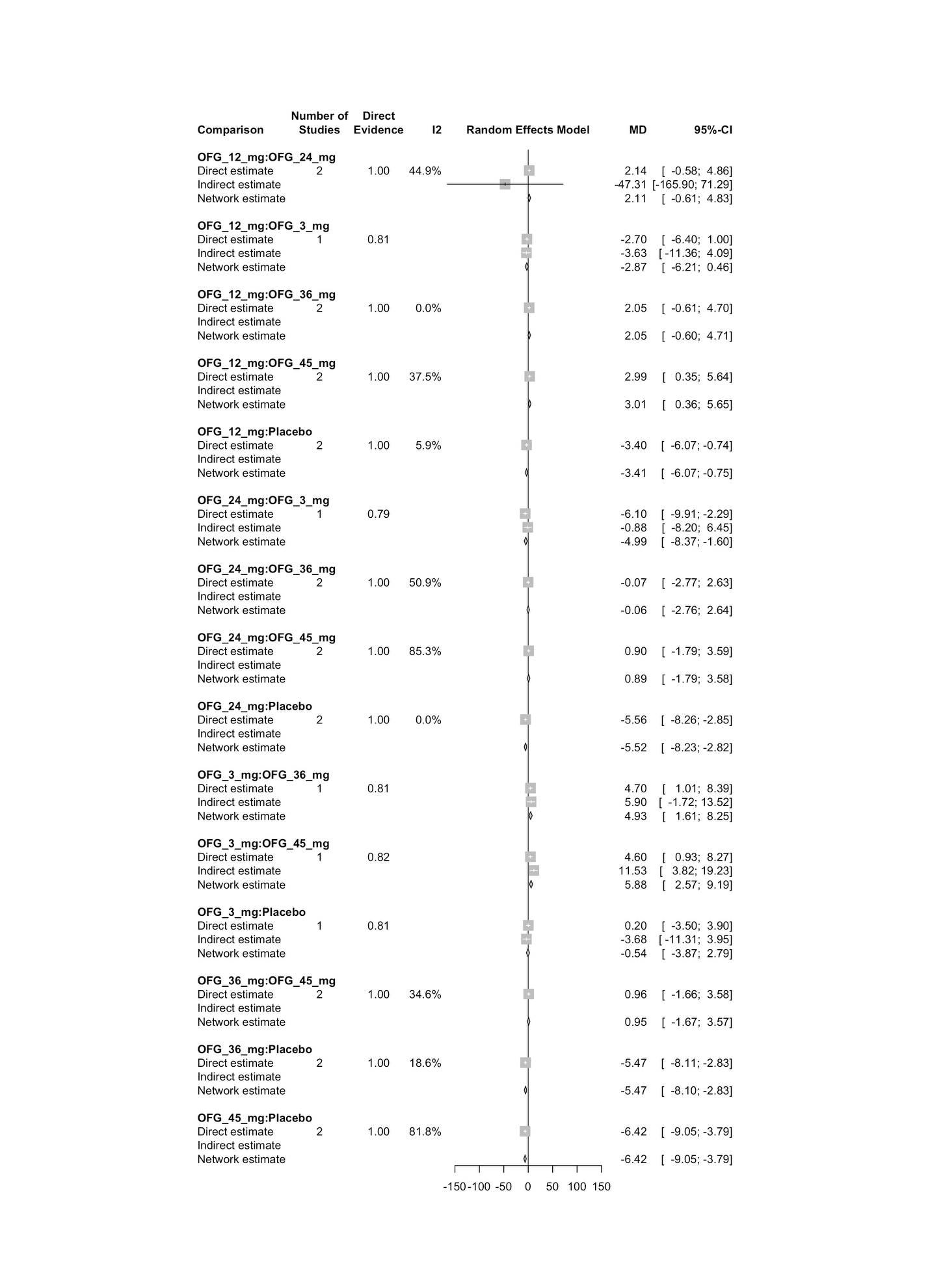


**Figure S28:** Side-splitting analysis for change from baseline in waist circumference (cm) at week 36.


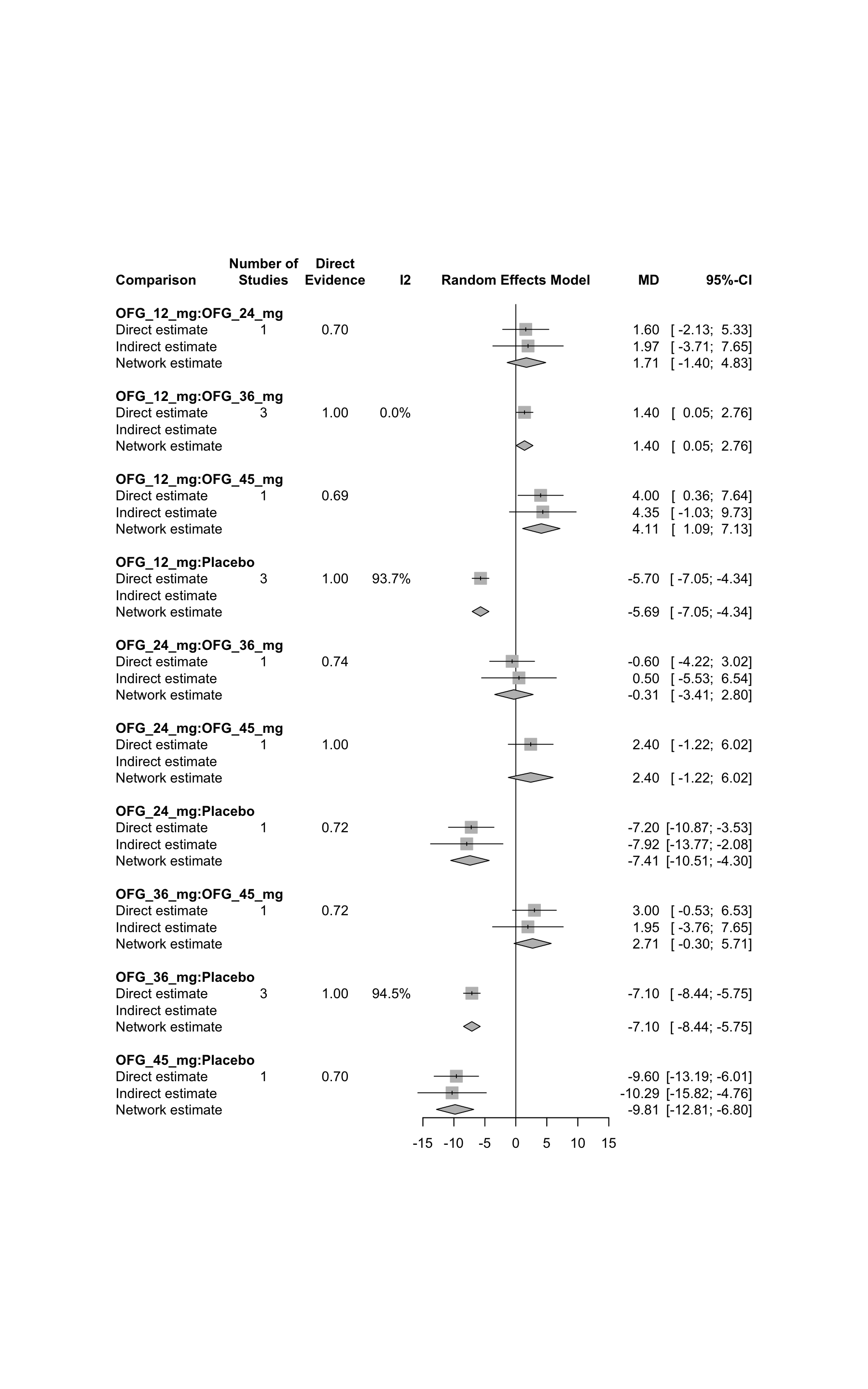


**Figure S29:** Side-splitting analysis for participants achieving ≥5% weight loss.


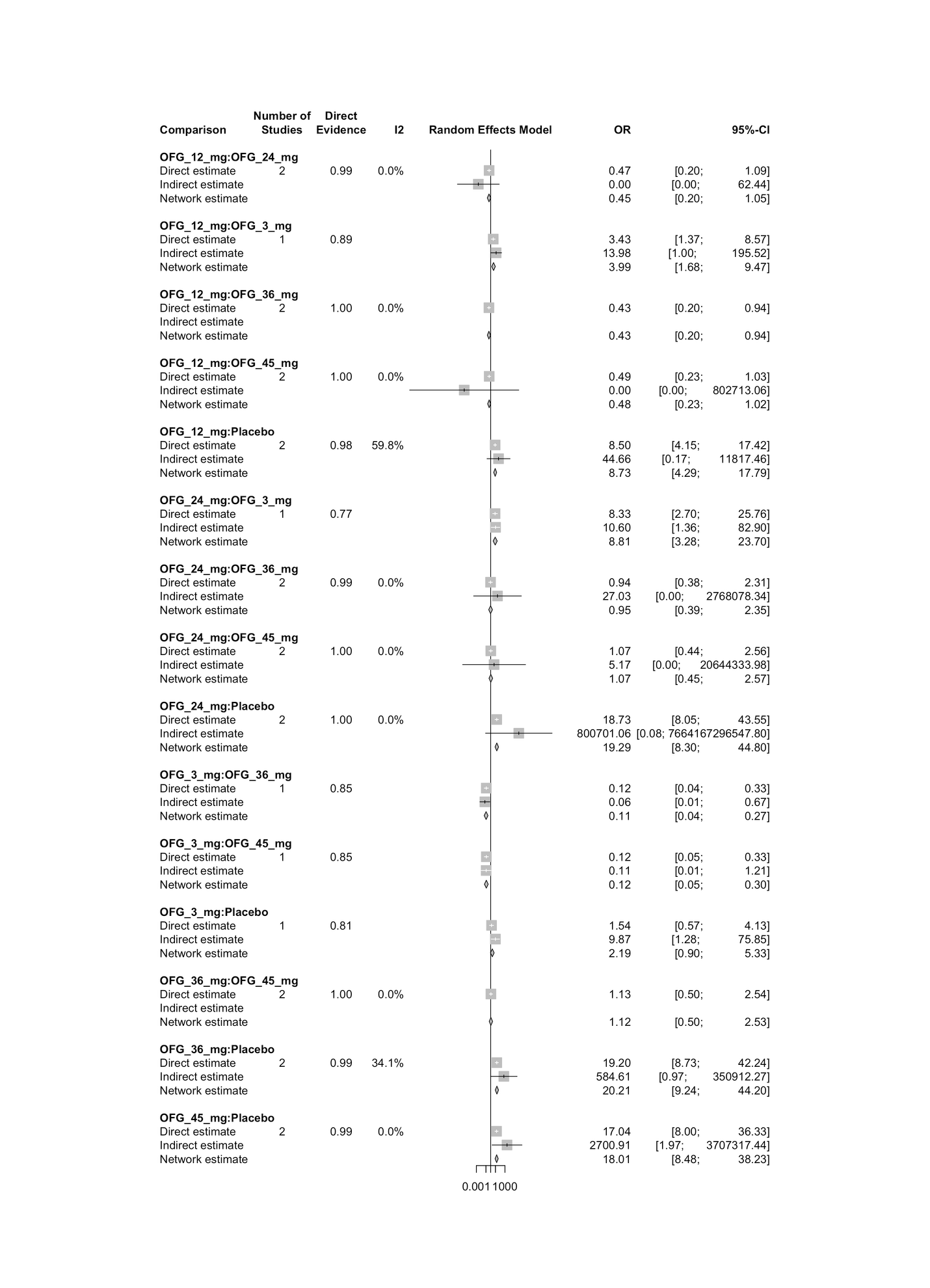


**Figure S30:** Side-splitting analysis for participants achieving ≥10% weight loss.


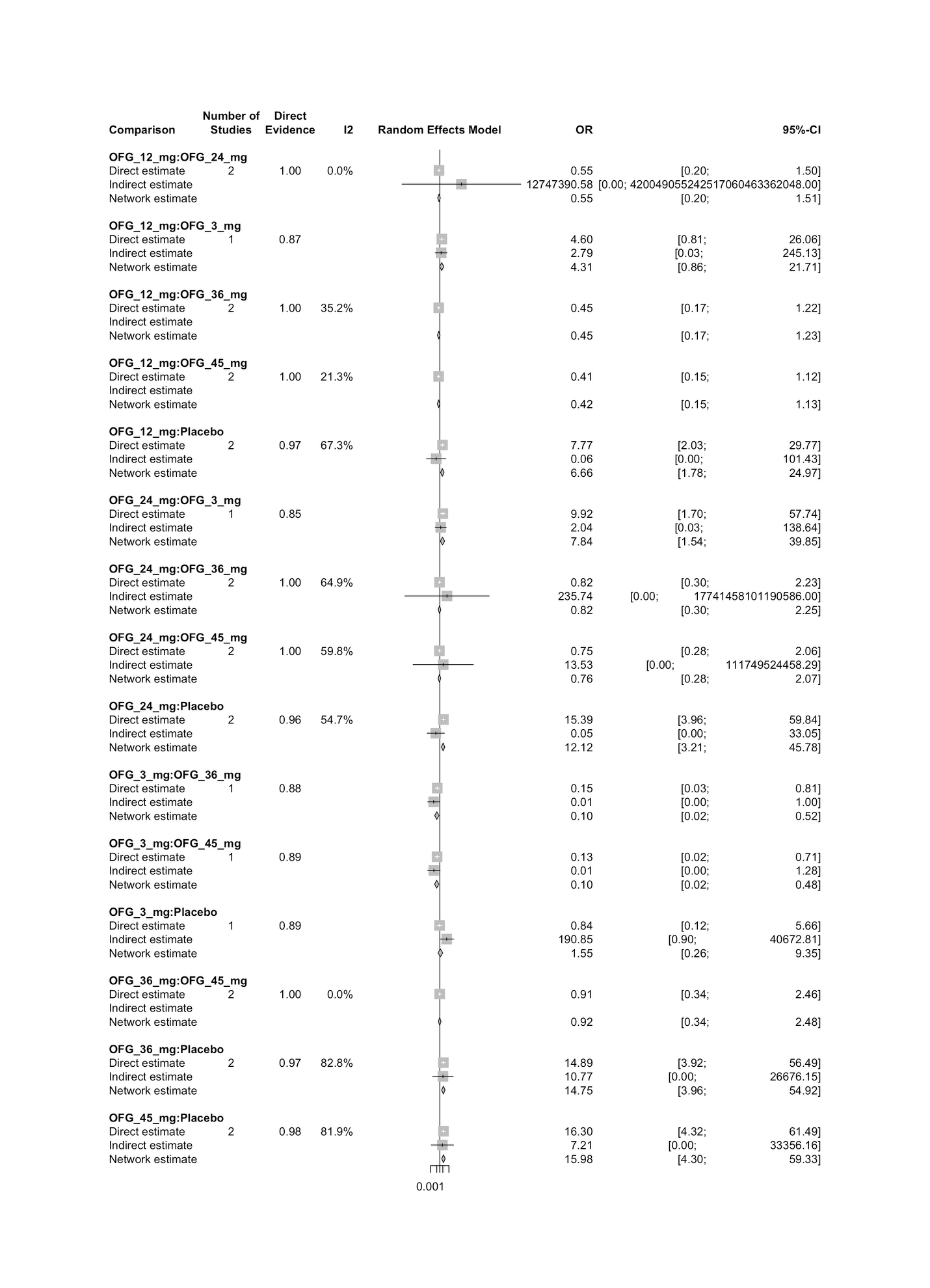


**Figure S31:** Side-splitting analysis for participants achieving ≥15% weight loss.


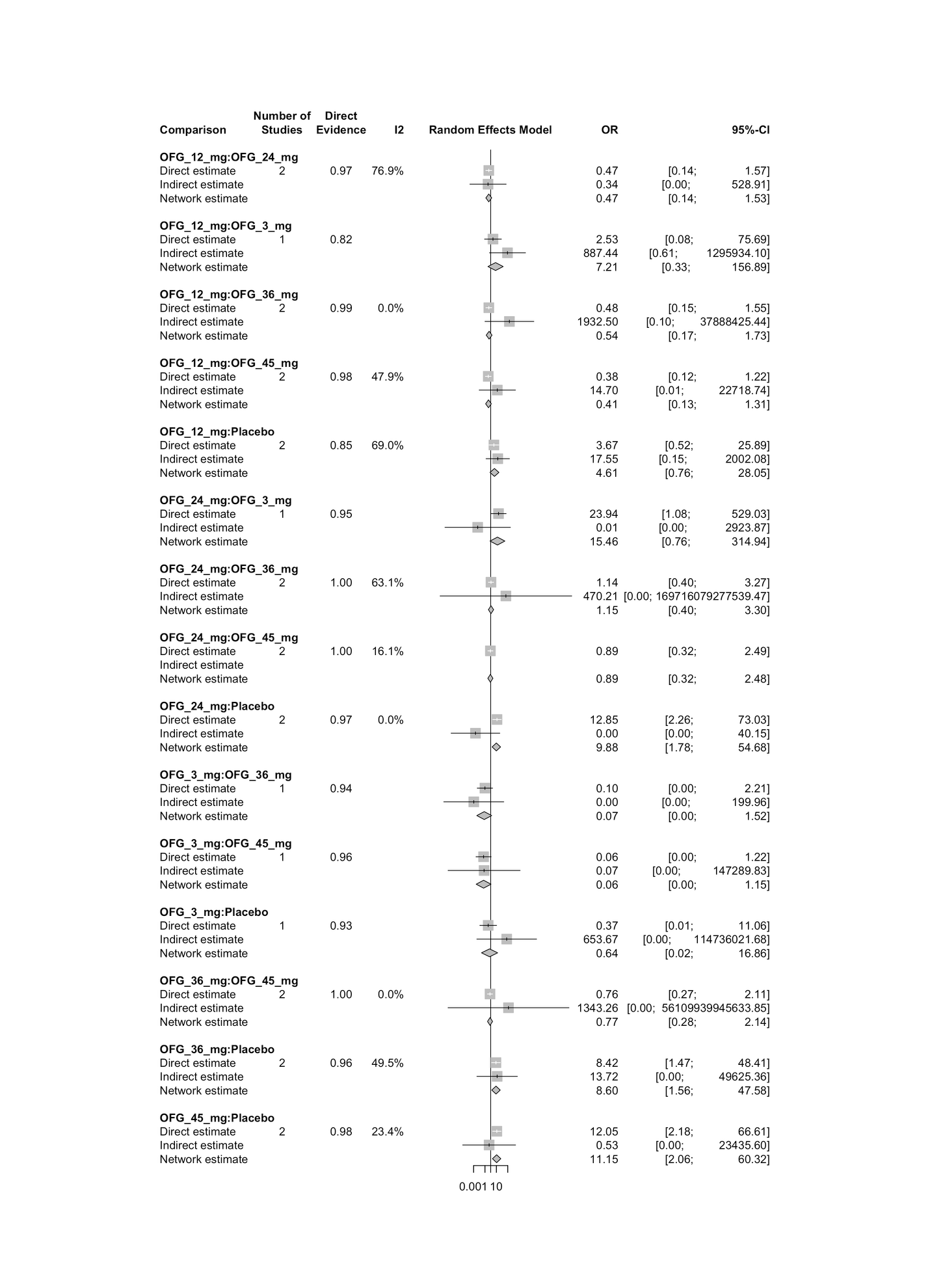


**Figure S32:** Side-splitting analysis for any TEAE.


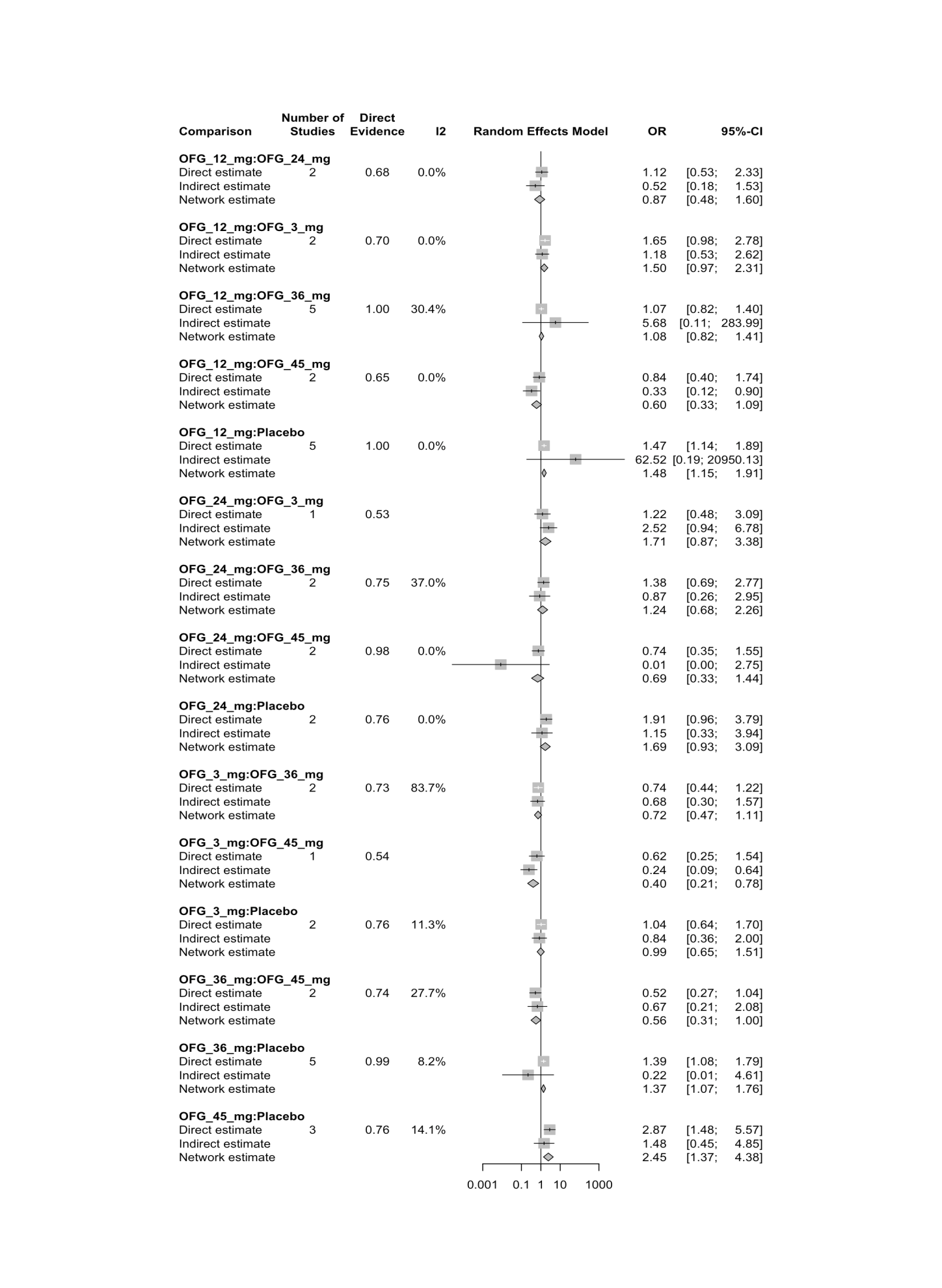


**Figure S33:** Side-splitting analysis for hypoglycaemia with plasma glucose <54 mg/dL.


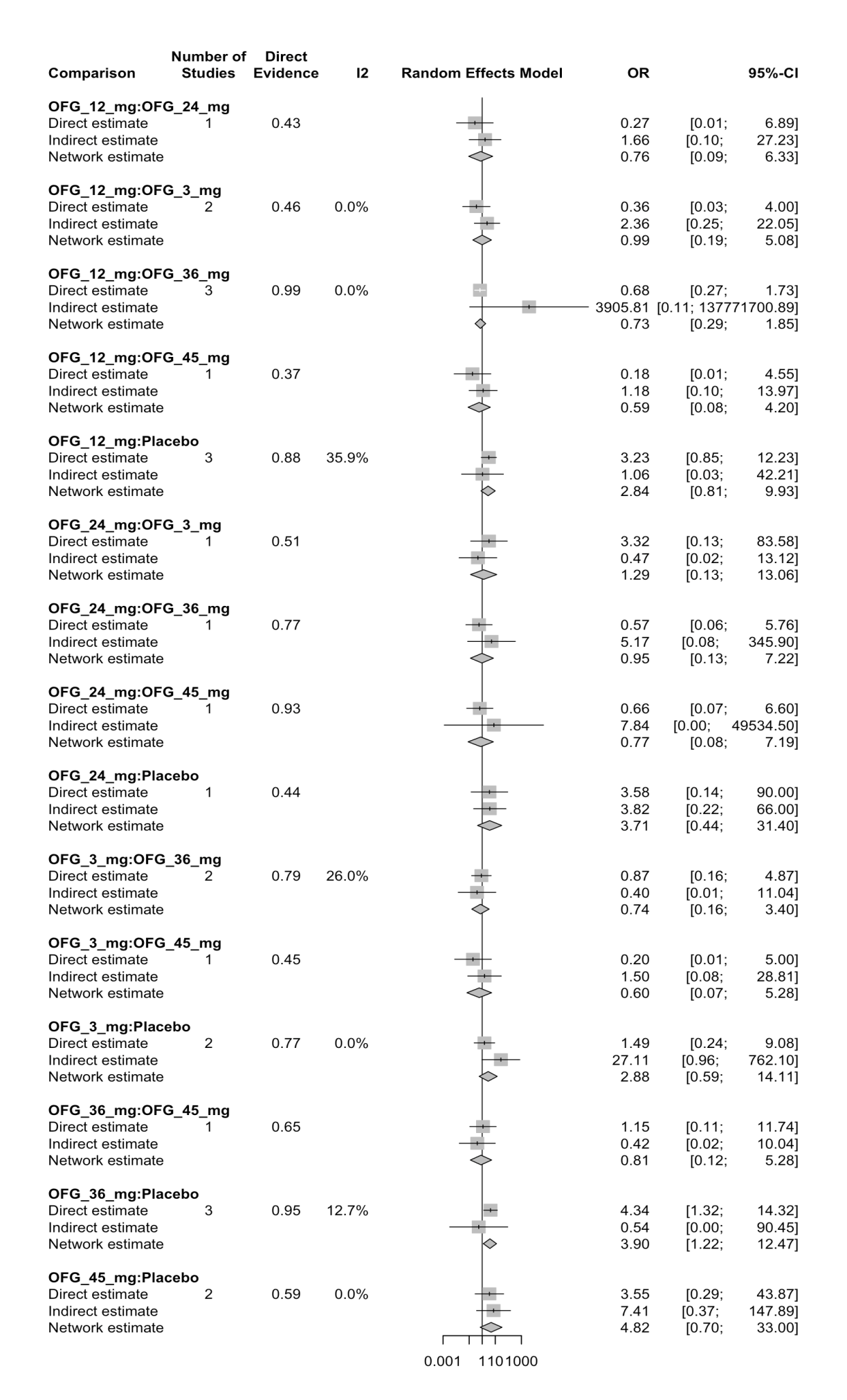


**Figure S34:** Side-splitting analysis for headache.


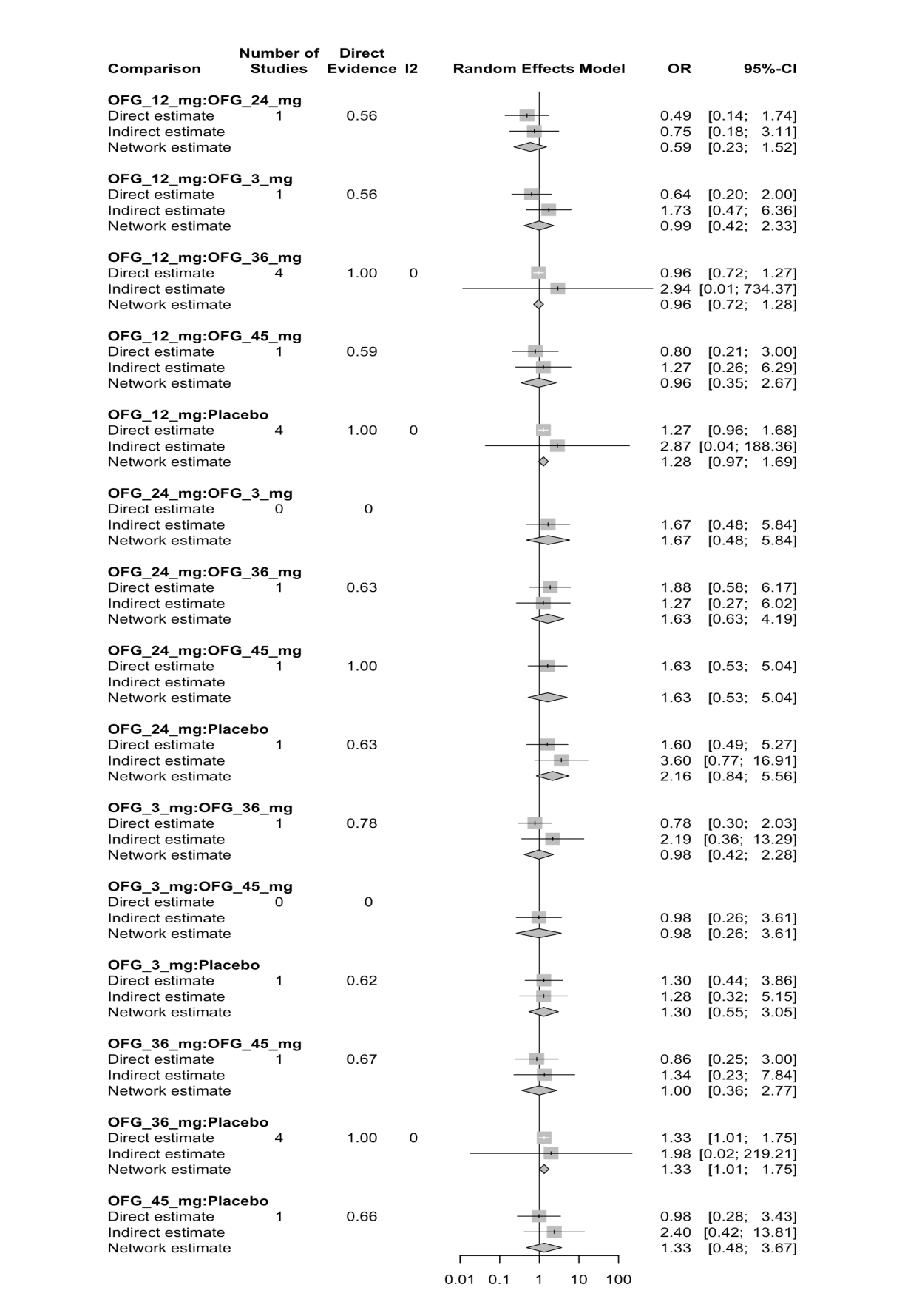


**Figure S35:** Side-splitting analysis for thyroid cancer.


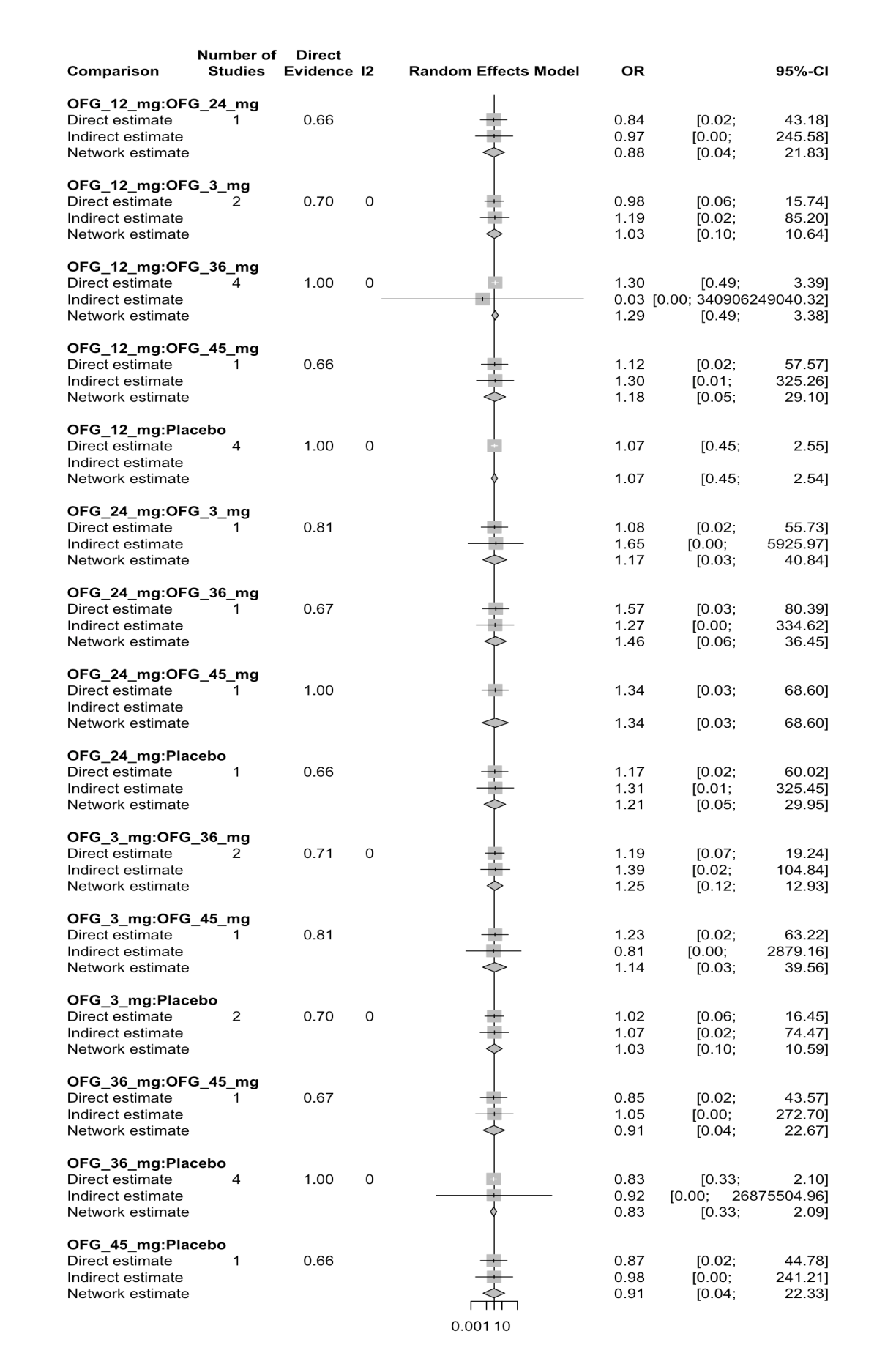


**Figure S36:** Side-splitting analysis for change in fasting glucose from baseline at week 12 (mg/dL).


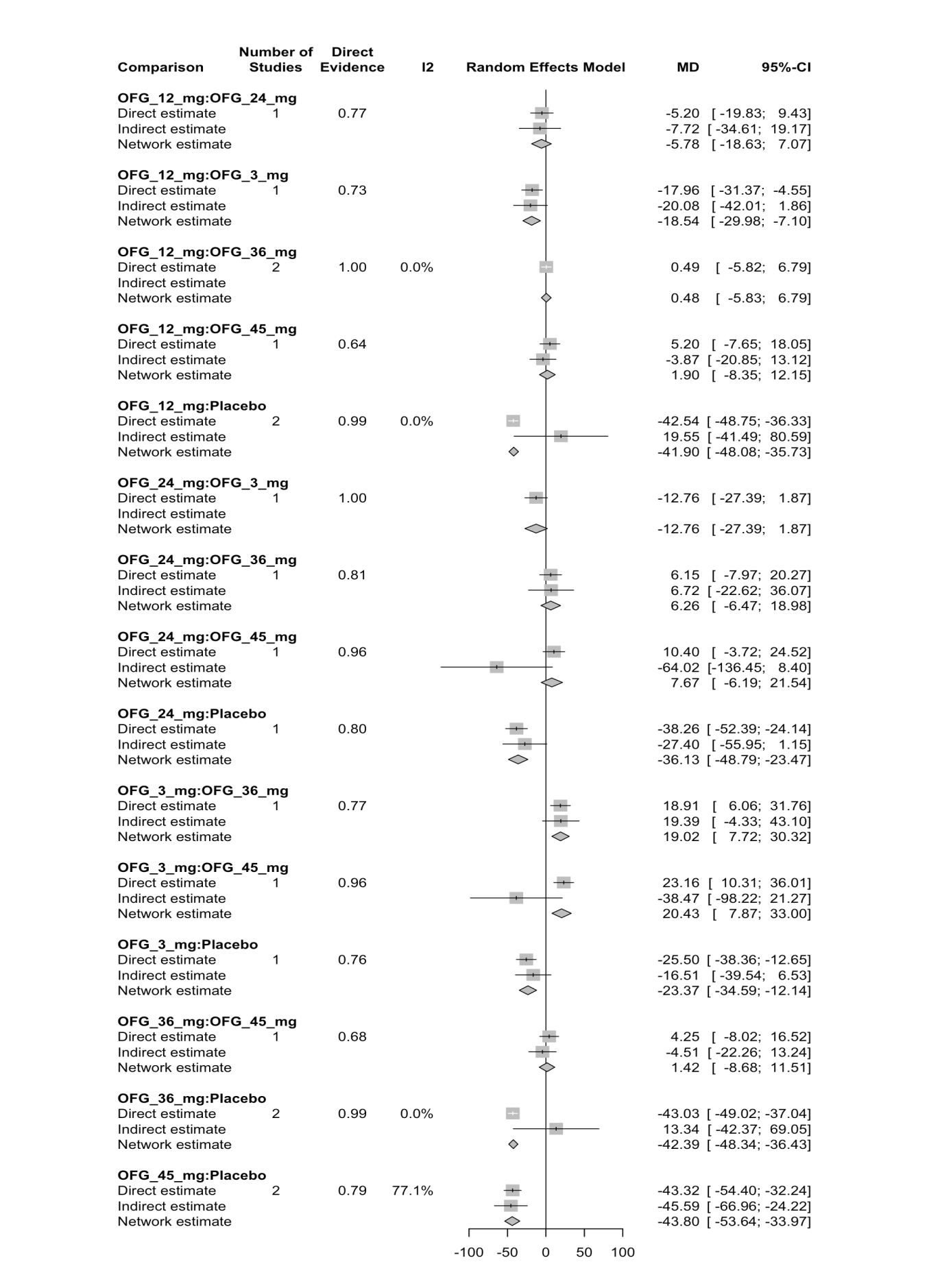


**Figure S37:** Side-splitting analysis for mean change in HbA1c (%) from baseline to week 12.


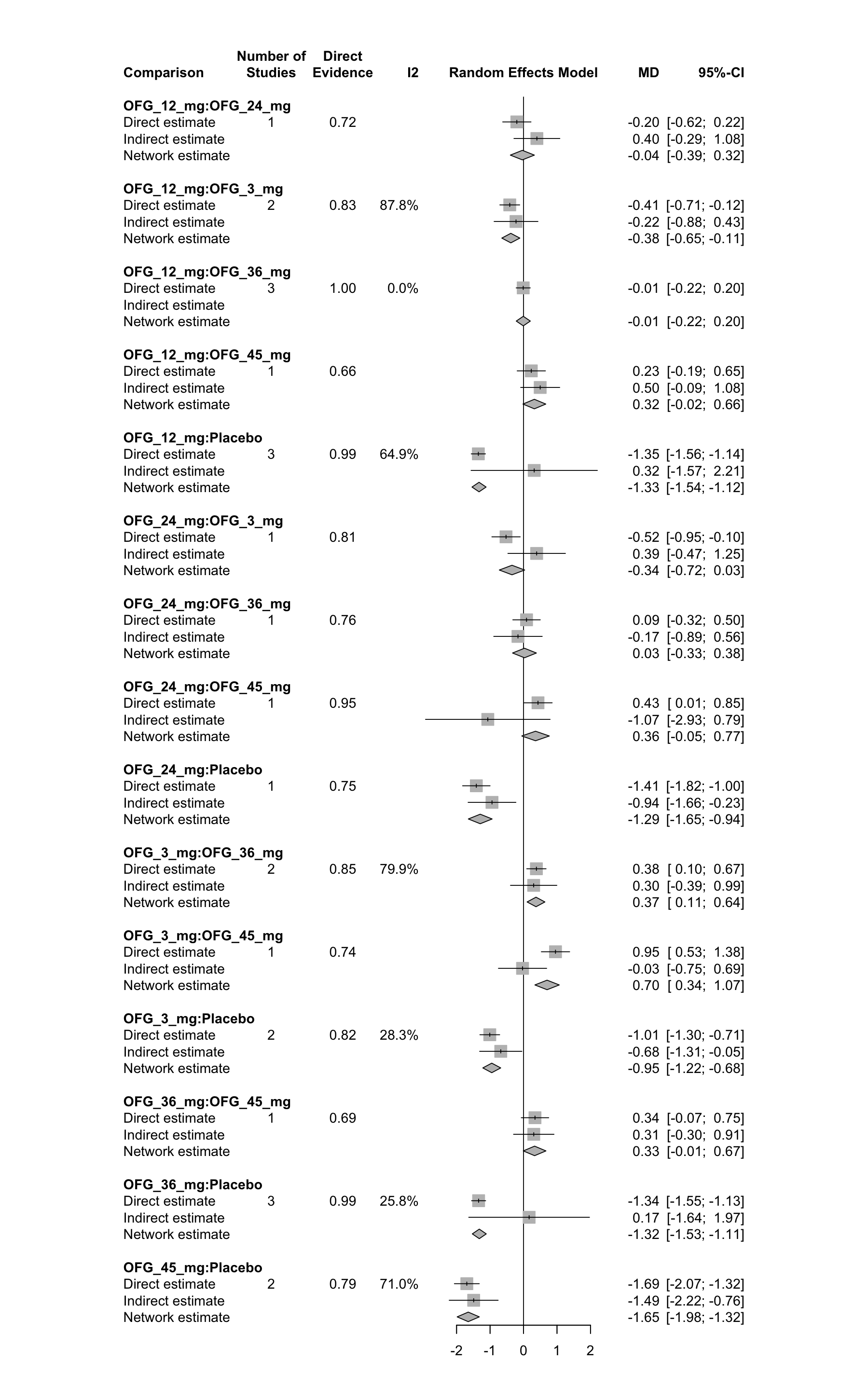


**Figure S38:** Side-splitting analysis for rescue therapy for severe, persistent hyperglycemia.


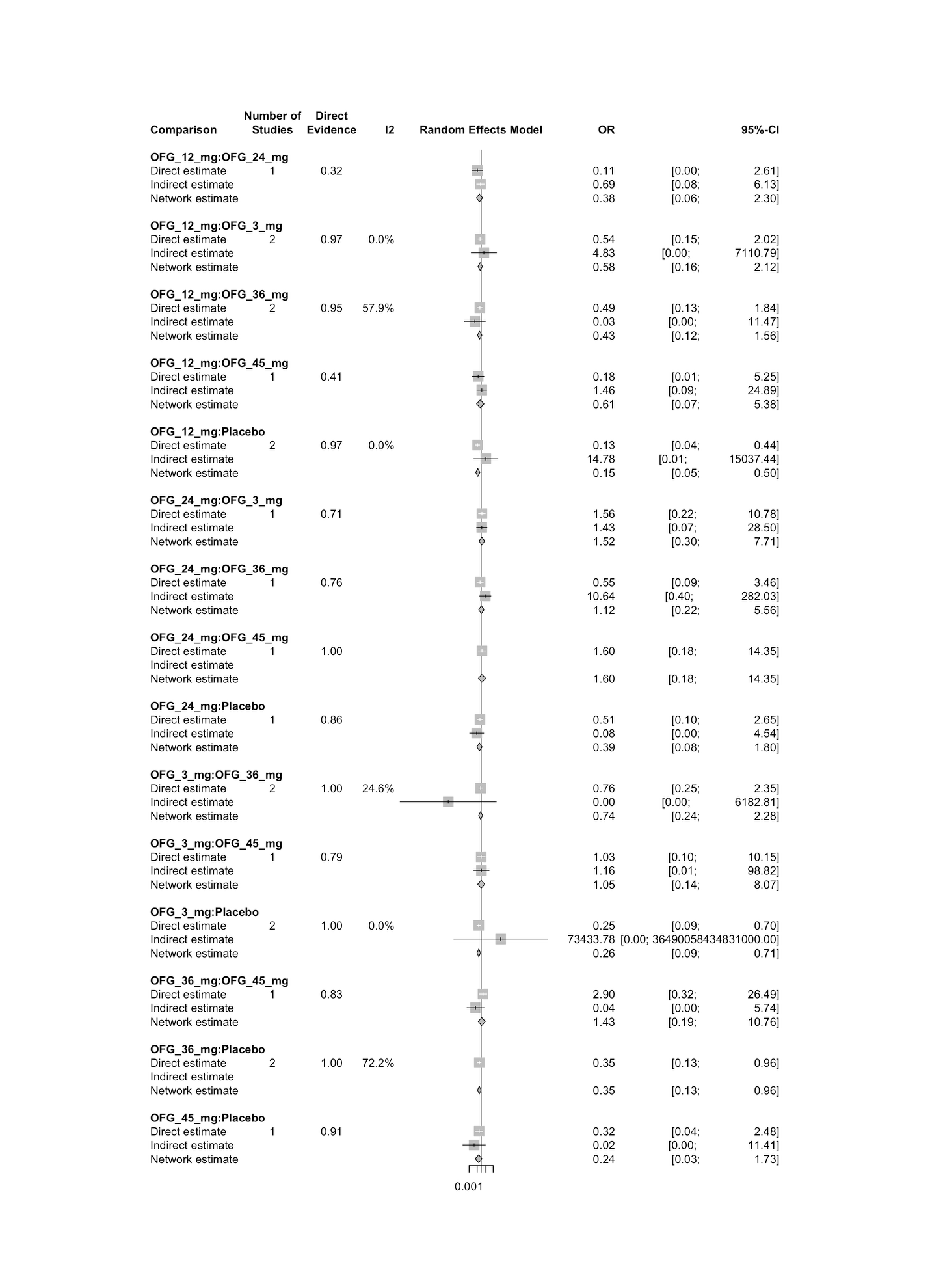


**Figure S39.** Sensitivity analysis for body weight (kg) change from baseline at week 12 after excluding small-sample studies.


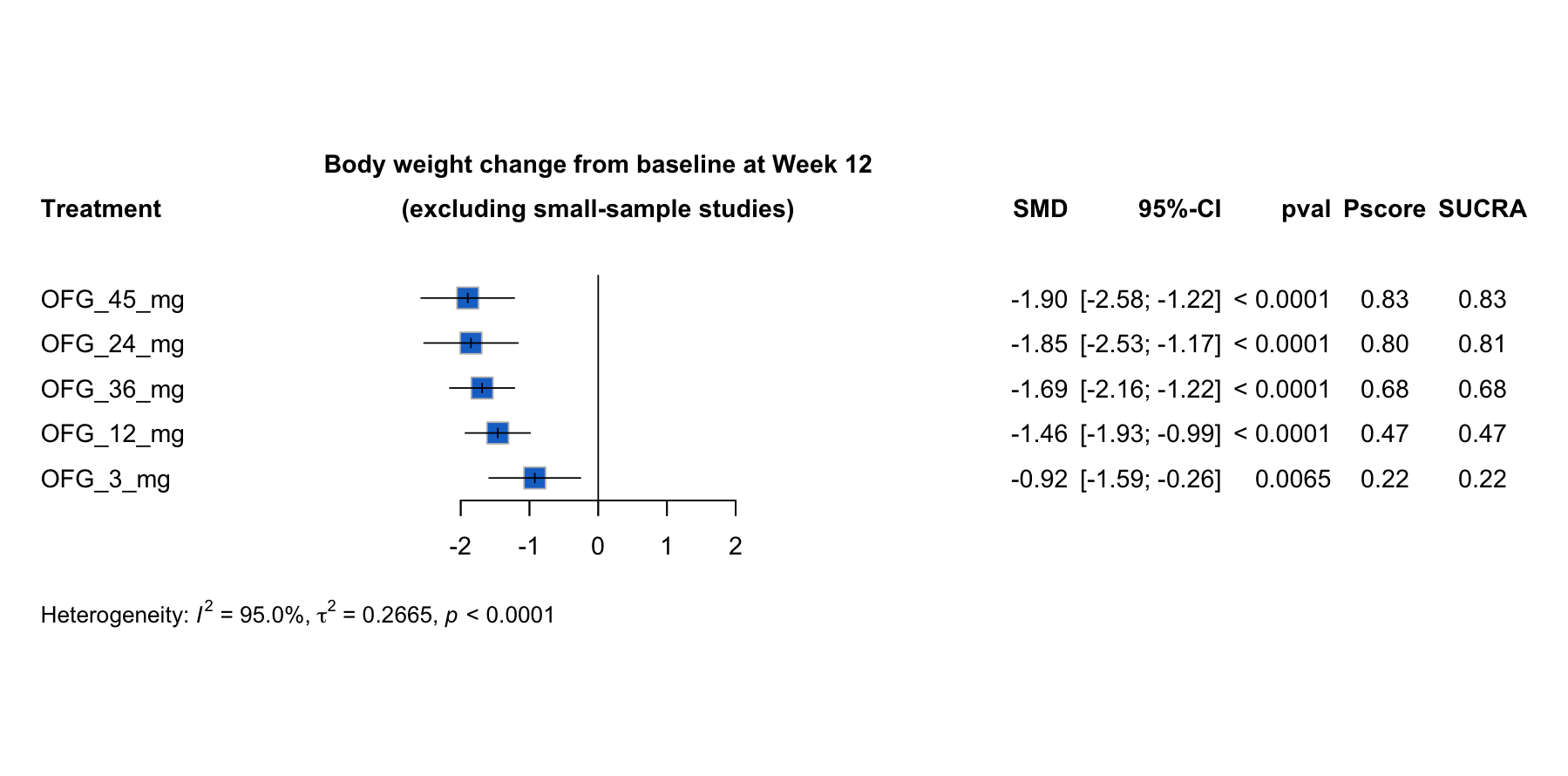


**Figure S40.** Sensitivity analysis for body weight (kg) change from baseline at week 12 after excluding phase I and II studies.


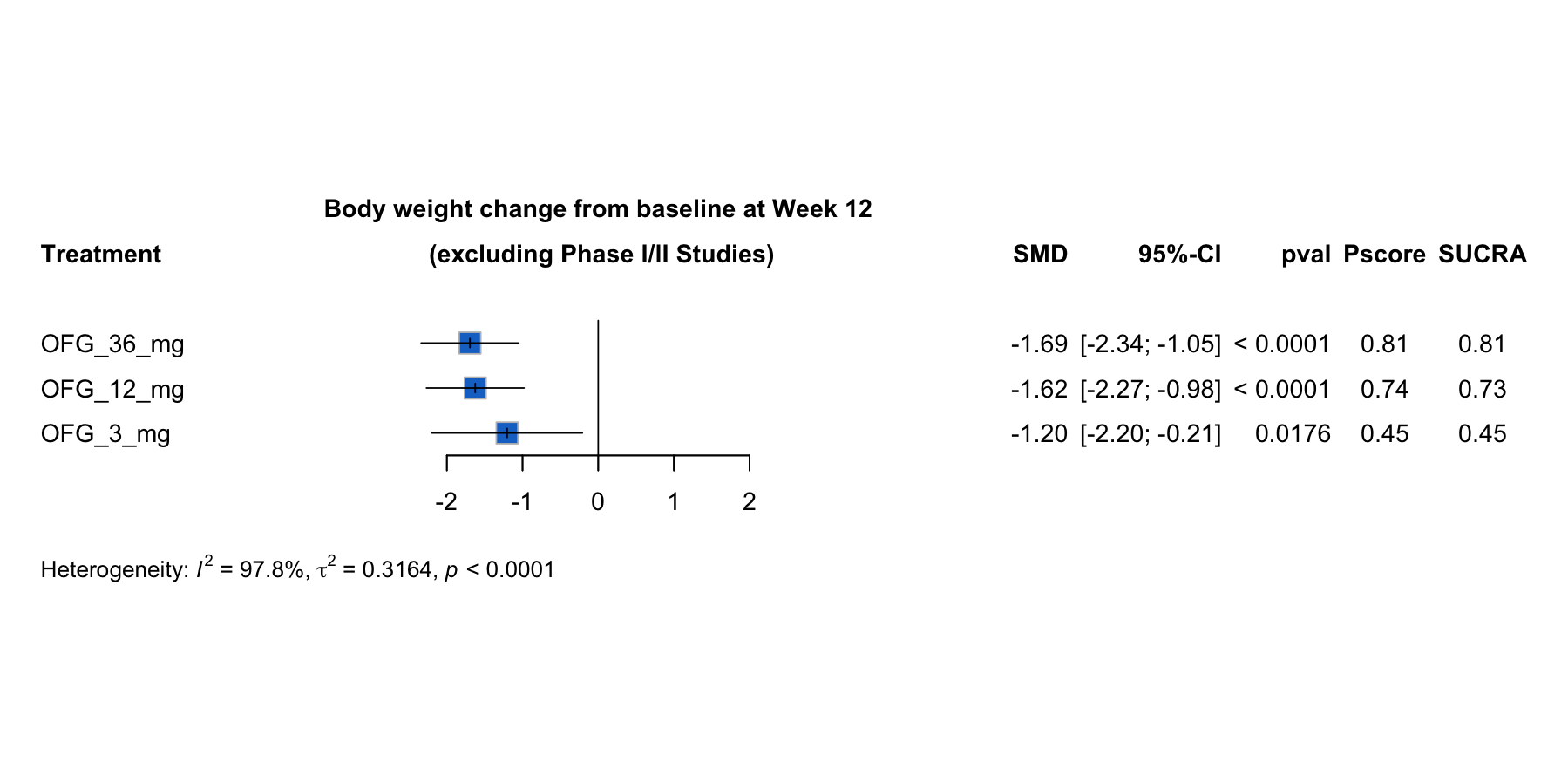


**Figure S41.** Sensitivity analysis for BMI change from baseline at week 12 after excluding phase I and II studies.


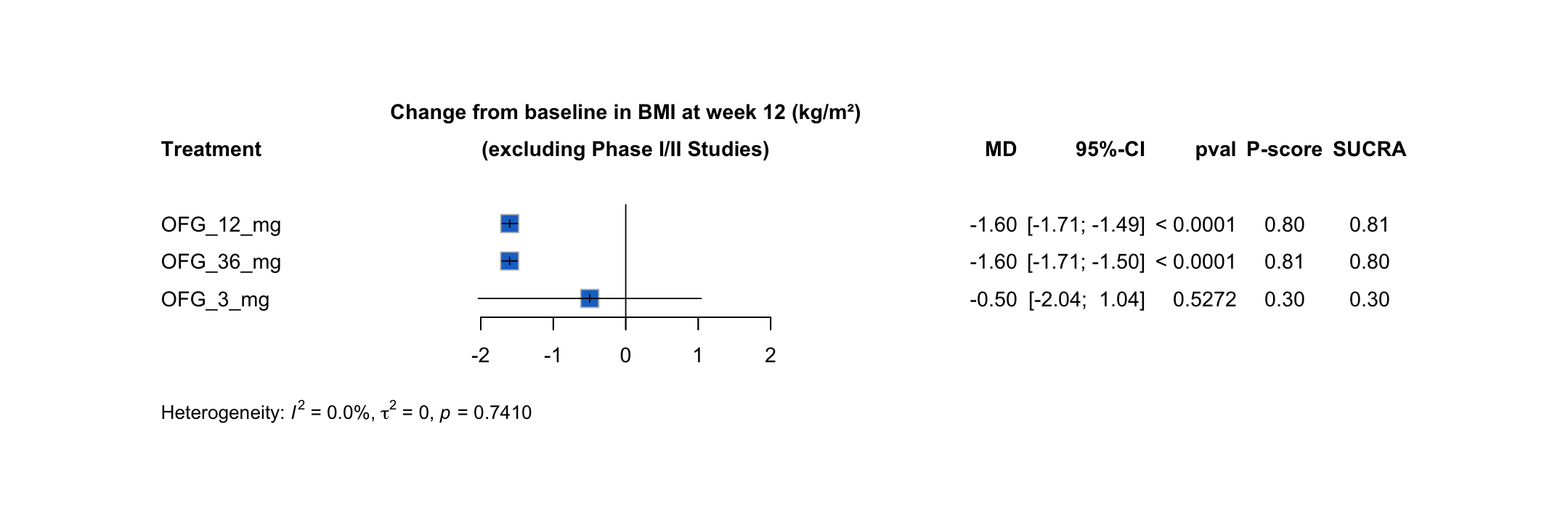


**Figure S42.** Sensitivity analysis for mean HbA1c (%) change from baseline to week 12 after exclusion of a small-sample study.


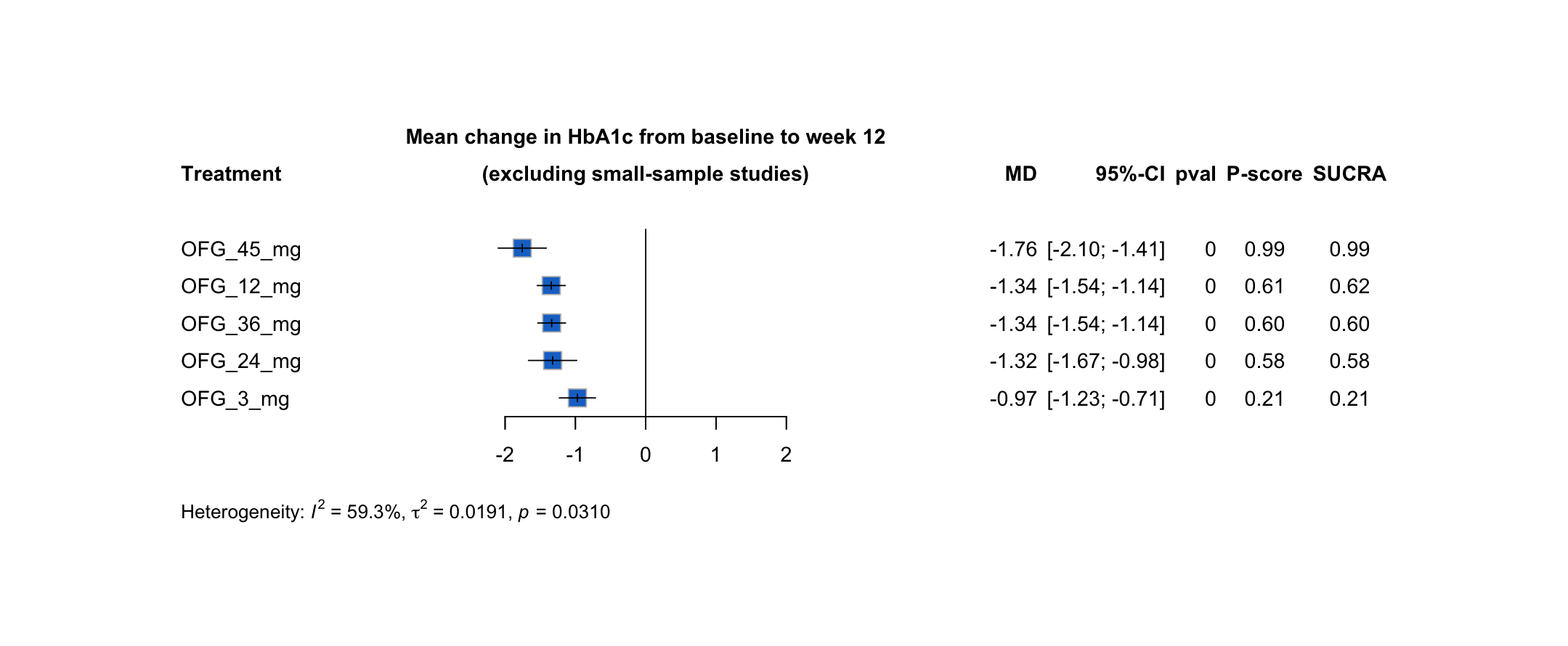


**Figure S43.** Sensitivity analysis for any TEAE, the findings after excluding small-sample studies.


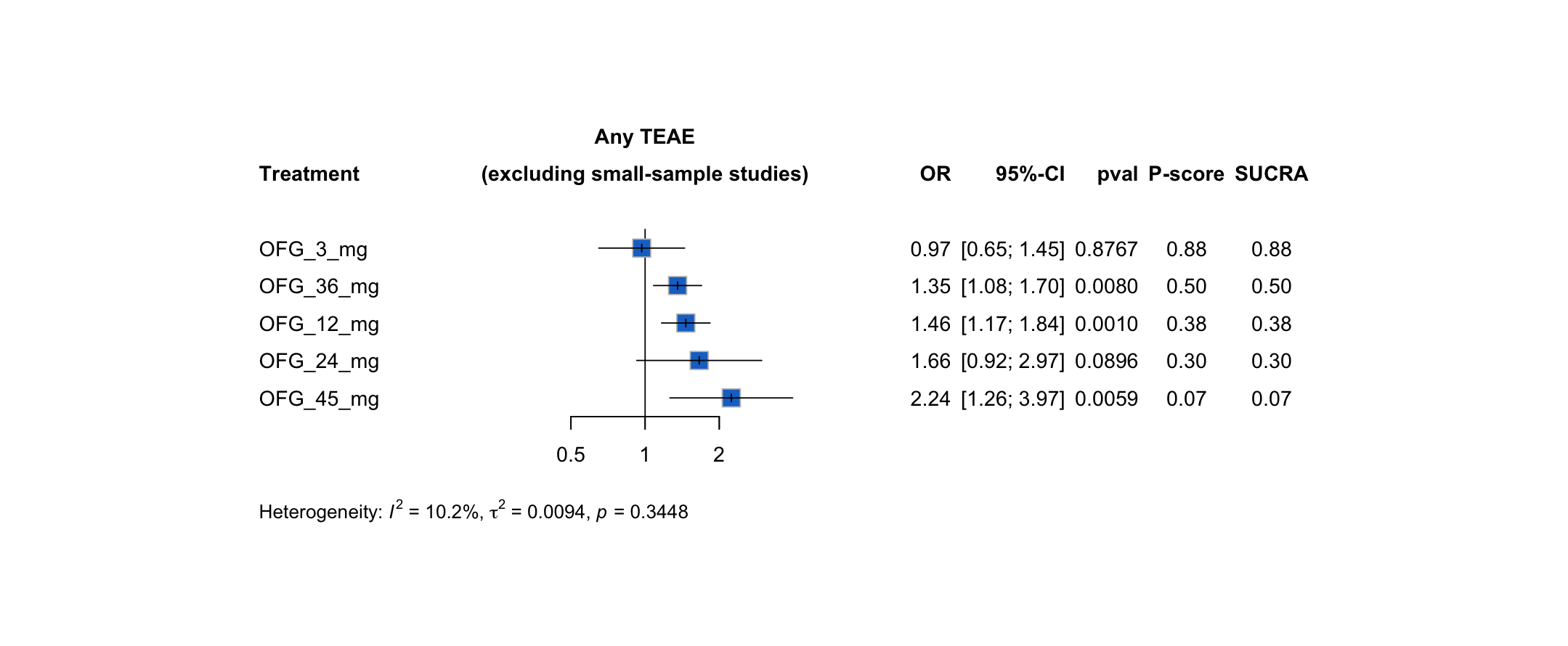


**Figure S44.** Sensitivity analysis for any TEAE after excluding phase I and II studies.


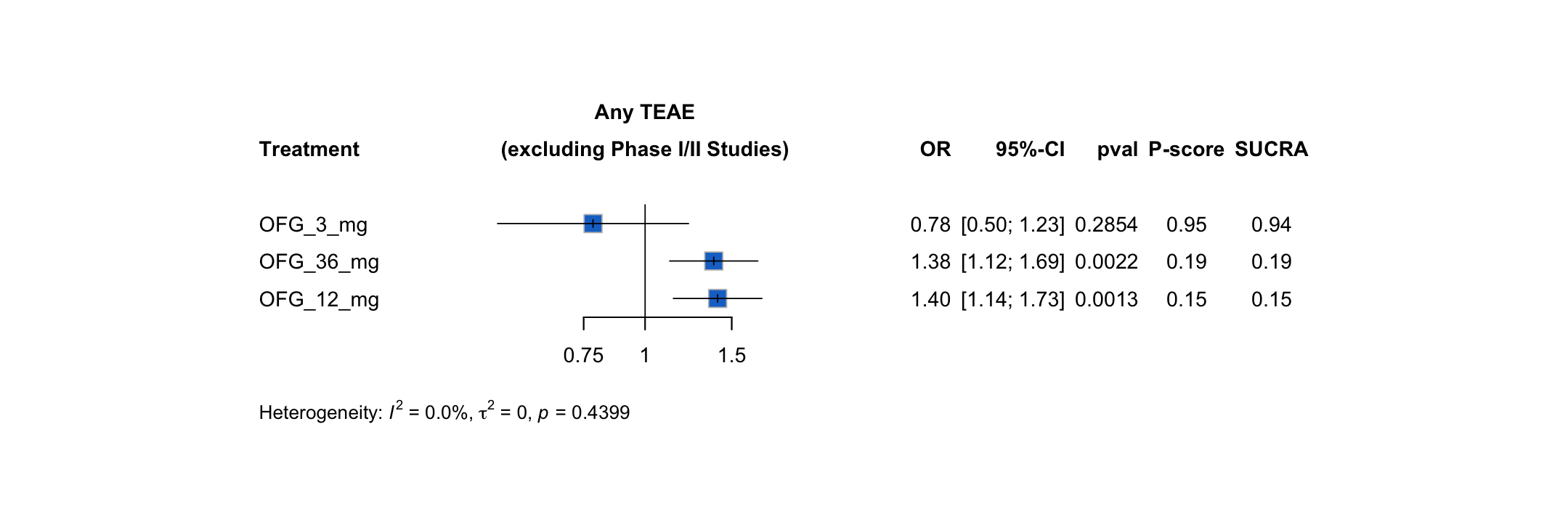


**Figure S45.** Sensitivity analysis for thyroid cancer after exclusion of phase I and II studies.


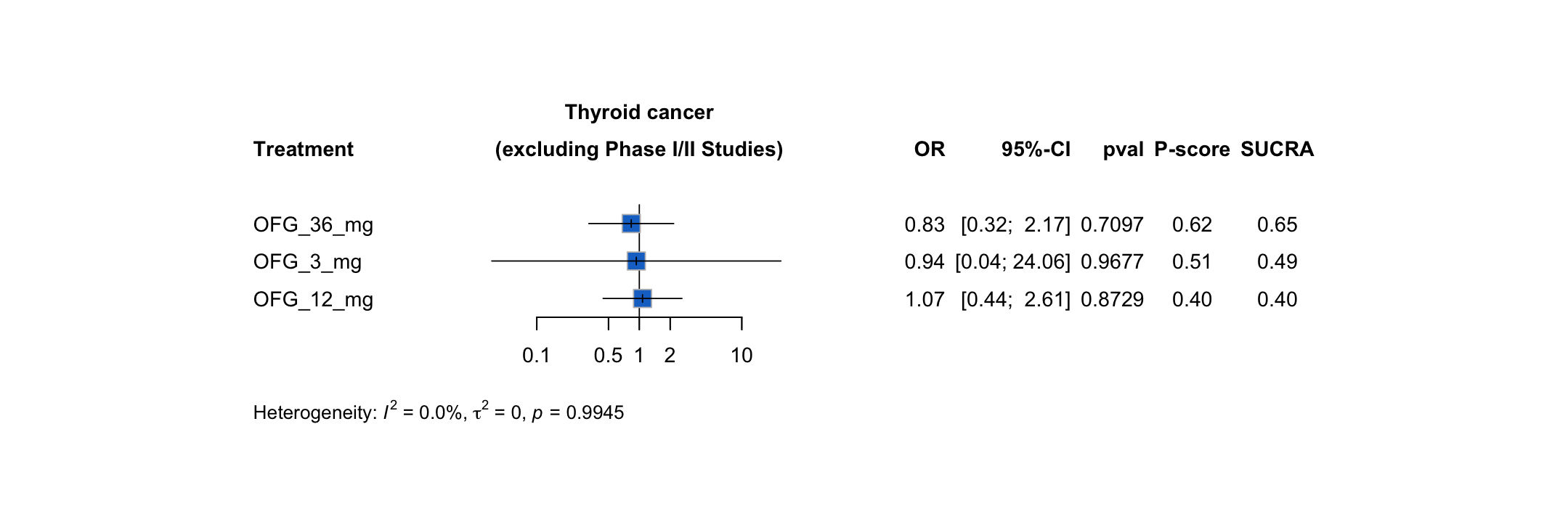


**Figure S46.** Sensitivity analysis for headache outcomes after exclusion of phase I and II studies.


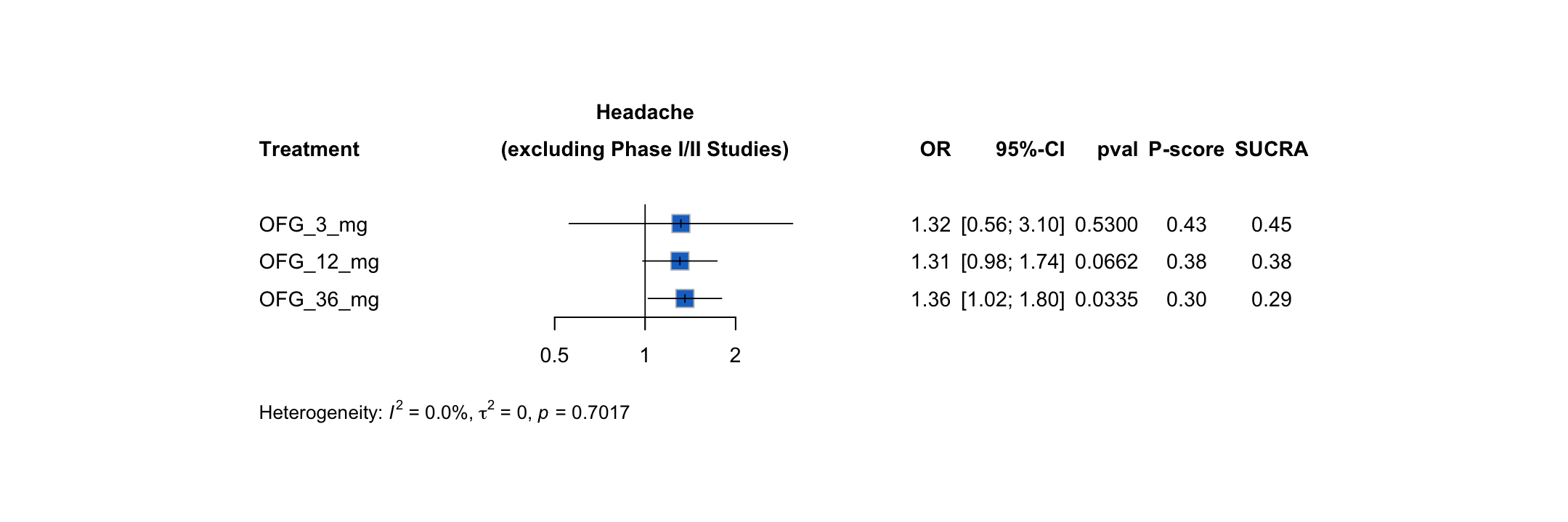

Supplement: Supplementary file 1 — Table S1: Detailed search strategy for each database. Table S2: Preferred Reporting Items for Systematic Reviews and Meta‐Analysis (PRISMA) checklist. Figure S1: Forest plots of categorical weight‐loss thresholds at week 26: (A) participants achieving ≥ 5% weight loss; (B) participants achieving ≥ 10% weight loss; (C) participants achieving ≥ 15% weight loss. Odds ratios (ORs) with 95% confidence intervals (CIs) are shown. Figure S2: Network graphs for categorical weight‐loss thresholds at week 26: (A) participants achieving ≥ 5% weight loss; (B) participants achieving ≥ 10% weight loss; (C) participants achieving ≥ 15% weight loss. Figure S3: Network graphs for glycemic outcomes: (A) mean change in HbA1c from baseline to week 12; (B) change in fasting glucose from baseline at week 12 (mg/dL); (C) rescue therapy for severe, persistent hyperglycemia. Figure S4: Forest plot for safety outcomes: (A) any TEAE; (B) hypoglycaemia with plasma glucose < 54 mg/dL; (C) headache; (D) thyroid cancer. Odds ratios (ORs) with 95% confidence intervals (CIs) are shown. Figure S5: Network graphs for safety outcomes: (A) any TEAE; (B) hypoglycaemia with plasma glucose < 54 mg/dL; (C) headache; (D) thyroid cancer. Figure S6: Subgroup analysis by diabetes status for total body weight change from baseline at week 12. Figure S7: Subgroup analysis by diabetes status for total body weight change from baseline at week 26. Figure S8: Subgroup analysis by diabetes status for total body weight change from baseline at week 36. Figure S9: Subgroup analysis by diabetes status for change from baseline in body mass index (kg/m2) at week 12. Figure S10: Subgroup analysis by diabetes status for change from baseline in body mass index (kg/m2) at week 26. Figure S11: Subgroup analysis by diabetes status for change from baseline in waist circumference (cm) at week 12. Figure S12: Subgroup analysis by diabetes status for change from baseline in waist circumference (cm) at week 26. Figure S13: Subgroup ana [file EDM2-9-e70275-s001.docx]
